# Coordination‐Driven Orthogonal Ligand Pairings through Dual Hydrogen‐Bonding/π‐π Interaction Complementarity

**DOI:** 10.1002/chem.202502411

**Published:** 2025-09-10

**Authors:** Jordan N. Smith, Yolanda Yau, Nina R. Lawson, Rosemary J. Goodwin, Dan Preston

**Affiliations:** ^1^ Research School of Chemistry Australian National University Canberra ACT 2610 Australia

**Keywords:** hydrogen bonding, molecular recognition, palladium(ii), self‐assembly, π‐π interactions

## Abstract

Multi‐layered and orthogonal recognition is an excellent route to controlled molecular complexity. Here we report a series of heteroleptic complexes where two ligands pair together at a palladium(II) metal centre in complementary fashion and with orthogonality to others pairs. This complementarity is driven in part through hydrogen‐bonding acceptor or donor sites proximal to the coordination domain (either DD:AA or AD:DA). These interactions alone are insufficient to control ligand pairing identity and/or orientation—secondary π‐π interactions between electron‐rich and electron‐poor aromatic groups in the recognition domain combine with the hydrogen bonding to give high levels of complementarity. Importantly, the different heteroleptic complexes can be combined without significant scrambling or mismatching.

## Graphical abstract

A series of bidentate ligands for square‐planar Pd(II) complexes were developed, each with hydrogen‐bonding and π‐π‐stacking domains that are complementary to only one other ligand. The bis‐bidentate complexes form orthogonally to one another with high fidelity, but only when both interaction types are exploited in tandem.

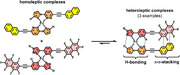

## 1 Introduction

**Figure 1:**
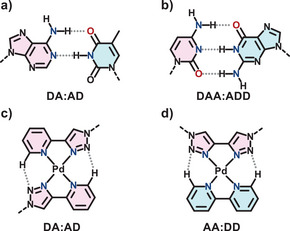
Orthogonal pairings. *Top*: between base pairings a) AT and b) CG, *Bottom*: previously reported^[^ ^44^ ^]^ coordination‐facilitated pairings c) DA:AD and d) AA:DD.

Specific and paired interactions or bonds lie at the heart of supramolecular self‐assembly. For example, the formation of coordination bonds relies on suitable combinations of ligands and metal ions, including the use of complementary denticity (i.e., 3:1 or 2:2 denticity with square planar metal ions).^[^ ^1^, ^2^, ^3^, ^4^, ^5^, ^6^, ^7^, ^8^ ^]^ Likewise, dynamic organic covalent bonds rely upon suitable pairings between, for example, aldehydes and amines or hydrazides to form imines and hydrazones, respectively.^[^ ^9^, ^10^, ^11^, ^12^ ^]^ To generate more complex assemblies, a key characteristic can be the presence of different and orthogonal pairings within the system. Nowhere is this better demonstrated than in DNA, where base pairs derive their complementarity to one another—and orthogonality to other pairings—through the number and identity of hydrogen bonding sites that each base possesses (Figure 1a and b). Scientists are interested in generating enhanced complexity and to this end have created “expanded” DNA alphabets.^[^ ^13^ ^]^ Synthetic complementary systems have also been generated, with work from the Hunter group focussing on complementary strands generated through both organic covalent complementarity and hydrogen bonding.^[^ ^14^, ^15^, ^16^ ^]^

Metallo‐supramolecular^[^ ^17^ ^]^ chemists are investing significant effort into increasing structural complexity,^[^ ^18^, ^19^, ^20^, ^21^, ^22^, ^23^, ^24^, ^25^, ^26^, ^27^, ^28^, ^29^, ^30^, ^31^, ^32^, ^33^, ^34^ ^]^ and so have also investigated orthogonal pairs of ligands that combine reliably when coordinated to metal ions. For example, Lehn and co‐workers have developed orthogonal metal‐ligand pairings where different metal ions with particular coordination preferences combine with different ligands of different denticity to create multiple orthogonal [**M**(L)(L′)] complexes,^[^ ^35^, ^36^ ^]^ and Schmittel and co‐workers have used similar approaches to drive the formation of complex low‐symmetry systems. In Schmittel's work, a key component is the use of steric bulk to force certain ligand combinations.^[^ ^37^, ^38^, ^39^ ^]^

Another way to enforce certain ligand combinations is through inter‐ligand hydrogen bonding. Here two different factors both lend themselves to the formation of a specific pairing: the denticity of the ligands in combination with the coordination geometry of the metal ion, and their hydrogen bonding capability. For example, numerous studies have exploited the capacity of bidentate 2‐pyridyl‐1,2,3‐triazole ligands to coordinate to square planar metal ions such as palladium(II) or platinum(II), where two bidentate ligands combine to coordinatively saturate the metal ion.^[^ ^40^, ^41^ ^]^ The ligands arrange themselves in a head‐to‐tail fashion to maximize hydrogen bonding between the N2 triazole nitrogen as an acceptor and the CH unit *ortho* to the coordinating pyridyl nitrogen as a donor, forming an AD:DA hydrogen bonding system (Figure 1c).^[^ ^42^, ^43^ ^]^ Key to this approach is the square‐planar geometry of the metal ions, which keeps hydrogen bond acceptors and donors in a single plane and in close proximity. Orthogonality can then be introduced through other bidentate combinations, for example between a (D)onor‐(D)onor ligand with two CH units and an (A)cceptor‐(A)cceptor ligand with two nitrogen atoms adjacent to the coordinative environment, giving an AA:DD pairing (Figure 1d). We have used this approach to demonstrate orthogonality in a general sense,^[^ ^44^ ^]^ and also as part of strategies to develop structurally complex multicomponent systems.^[^ ^45^, ^46^, ^47^ ^]^

When using complementarity to control connectivity, higher complexity will arise through increasing the overall number of complementary pairs. We considered that we might expand our pairing system through appending our hydrogen‐bonding ligands with secondary aromatic units that were either electron rich (i.e., naphthalene) or electron poor (i.e., heptafluoronaphthalene) in character. We report here three new pairings based upon hydrogen bonding and aromatic complementarity, together with model studies carried out on model ligands and homoleptic ligand complexes in order to fully investigate their behavior and tease out contributing factors to our molecular recognition system.

## 2 Results and Discussion

### 2.1 Ligand Design

**Scheme 1:**
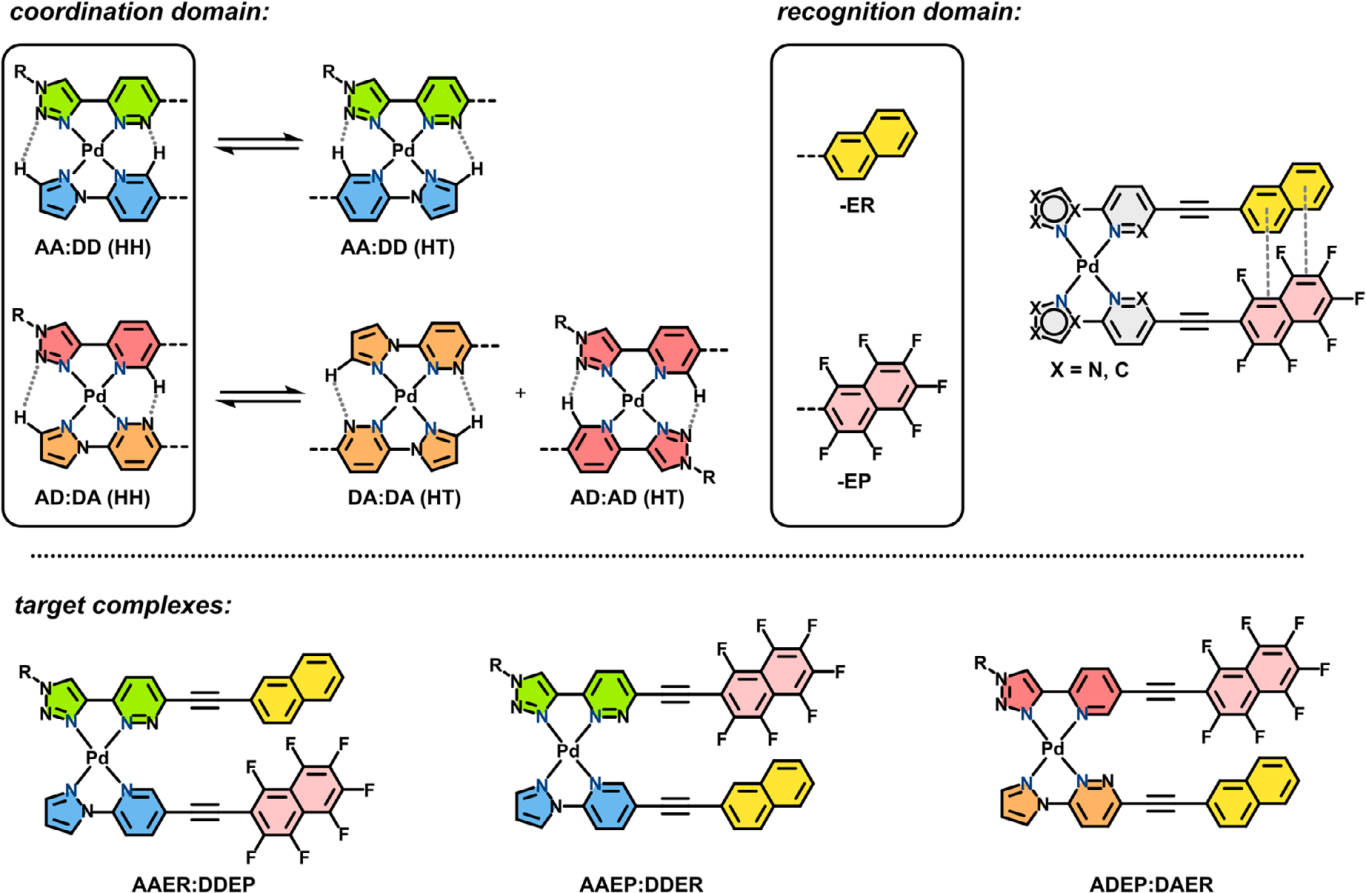
General structures of the coordination and recognition domains, and the three target heteroleptic complexes.

We designed two heteroleptic ligand pairings for bis‐bidentate, square‐planar Pd(II) complexes: **AD:DA** and **AA:DD** (Scheme 1). The pairing **AD:DA** comprises triazole‐pyridine (**AD**) and pyrazole‐pyridazine (**DA**) ligands, where the H‐donor and acceptor positions may interact across the coordination sphere to stabilize the complex. However, this system is imperfect, as both ligands are also self‐complementary for homoleptic complex formation that may result in homo‐ and heteroleptic scrambling. The pairing **AA:DD** combines triazole‐pyridazine (**AA**) and pyrazole‐pyridine (**DD**) ligands, but again is complicated by head‐to‐head (HH) and head‐to‐tail (HT) isomerism as H‐bonding is complementary in both orientations. In both cases, the bidentate *coordination domain* alone is insufficient for high selectivity toward a single isomer.

We reasoned that the addition of a further point of interaction—the *recognition domain*—could provide a means of control over HH:HT and homoleptic:heteroleptic scrambling. The complementary aromatic units 2‐naphthalene (electron‐rich; **ER**) and 2‐heptafluoronaphthalene (electron‐poor; **EP**) were selected, as favorable face‐to‐face π‐π stacking interactions could stabilize a desired complex. Work from us^[^ ^48^, ^49^, ^50^ ^]^ and many others^[^ ^51^, ^52^, ^53^, ^54^, ^55^, ^56^, ^57^, ^58^, ^59^, ^60^, ^61^, ^62^ ^]^ has demonstrated that π‐π interactions are enhanced when between aromatic systems of opposing electrostatic distributions, including work from Dougherty, Grubbs and co‐workers using aromatic rings with either hydrogen or fluorine atoms on the carbon atoms.^[^ ^63^ ^]^ Further, the combination of four coordination‐ and two recognition‐domains gives eight distinct ligands and four orthogonal pairings—a suitably complex system for data encoding. Seven of these ligands were accessible synthetically (**AAER**, **AAEP**, **ADER**, **ADEP**, **DAER**, **DDER** and **DDEP**); **DAEP** could not be isolated despite attempting multiple synthetic pathways. Hence, Pd(II) complexes of the three orthogonal pairings **AAER:DDEP**, **AAEP:DDER** and **ADEP:DAER** were studied in DMSO. While we recognise this solvent system may limit the scope of future applications in self‐assembly, the R‐group of the triazole fragment is easily modified to increase the solubility of these ligands in other desirable solvents.

### 2.2 The Coordination Domain: Model Complexes

Before assessing the orthogonality of our system, we needed to understand the behavior of the coordination domains in isolation, including the preference toward homo‐ or heteroleptic complex formation. We prepared the series of model ligands bearing trimethylsilane (TMS) groups that cannot form complementary interactions in the recognition domain: **AA‐TMS**, **AD‐TMS**, **DA‐TMS**, and **DD‐TMS**.

For complex formation, each ligand was combined in [D_6_]DMSO with tetrakis(acetonitrile)palladium(II)tetrafluoroborate in a 2:1 L:M ratio, and the mixture analyzed by ^1^H nuclear magnetic resonance (NMR) spectroscopy and high‐resolution electrospray ionization (ESI) or Nanospray mass spectrometry. Pd(II) was selected for its preference to four‐coordinate square‐planar complexes and hemi‐lability: the thermodynamic product is formed rapidly at room temperature. To exclude any influence of the TMS group on the isomer formed, complexes were also formed using the deprotected, terminal‐alkyne ligands. These showed equivalent behaviors, but were prone to decomposition and not suitable for extensive studies (see SI; Figures S46‐48). Where multiple species exist in equilibrium, the ratio was determined by ^1^H NMR integration, and the results are summarized in Table 1.

**Table 1:** Summary of Pd(II) complexes formed with model ligands.

	mole fraction		
**ligand**	**M_1_L_1_^[^** ^a^ ^]^	**M_1_L_2_^[^** ^b^ ^]^	**isomer**
**AD‐TMS**	N.D.	>0.95	HT
**DA‐TMS**	N.D.	>0.95	HT
**AA‐TMS**	0.17	0.83	HT
**DD‐TMS**	0.60	0.40	HT

N.D.: not detected.

^[a]^ Species [Pd(L)(DMSO)_2_]^2+^.

^[b]^ Species [Pd(L)_2_]^2+^.

**Figure 2:**
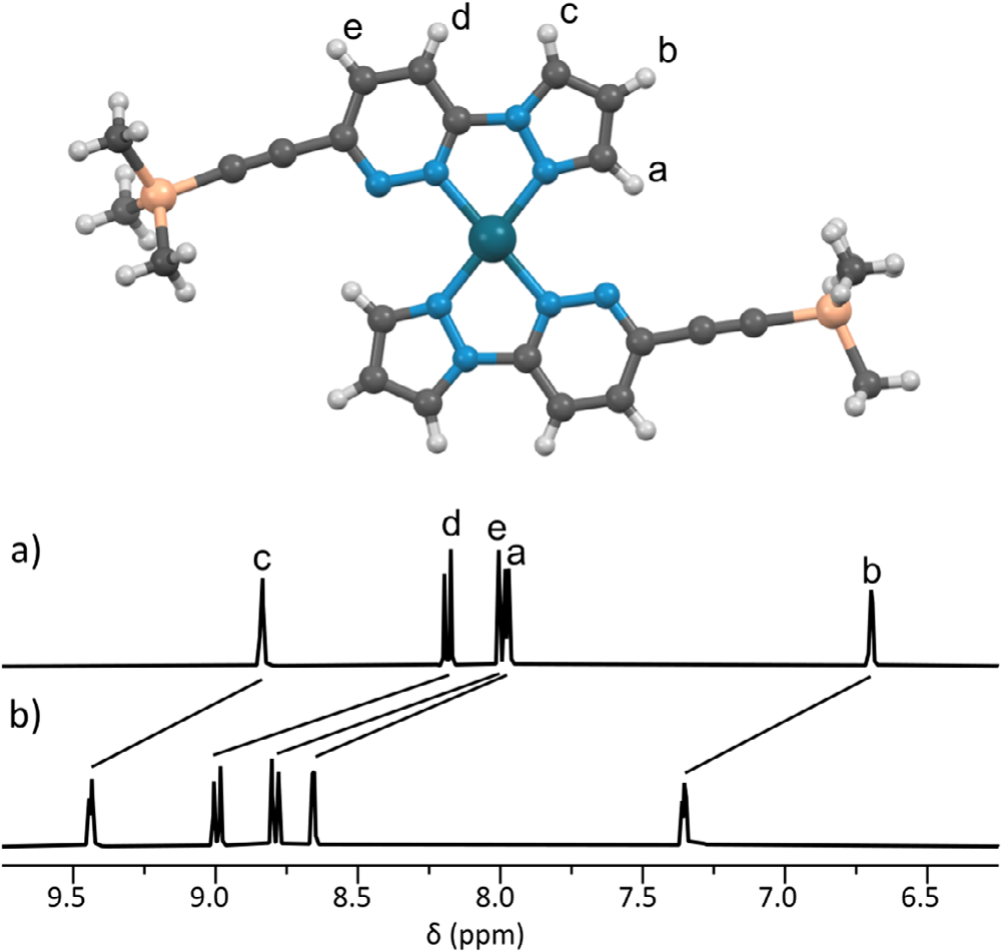
X‐ray crystal structure of [Pd(DA‐TMS)_2_](BF_4_)_2_ (counterions omitted for clarity), and partial stacked ^1^H NMR spectra ([D_6_]DMSO, 400 MHz, 298 K) of a) ligand DA‐TMS; and b) DA‐TMS with 0.5 eq. Pd(II).

The homoleptic complexes **[Pd(DA‐TMS)_2_]^2+^** and **[Pd(AD‐TMS)_2_]^2+^** formed quantitatively, showing significant downfield shifts of key resonances in the ^1^H NMR spectrum upon complexation, and evidence of the M_1_L_2_ species by HR‐MS. The ^1^H NMR spectra of **DA‐TMS** and **[Pd(DA‐TMS)_2_]^2+^** are shown as an example in Figure 2. Both complexes were assigned as head‐to‐tail, as only in this orientation are the H‐bonding donors and acceptors oriented to maximize favorable interactions.^[^ ^40^, ^41^, ^44^ ^]^ Single‐crystal X‐ray diffraction (SCXRD) structures^[^ ^64^ ^]^ for both **[Pd(AD‐TMS)_2_]^2+^** and **[Pd(DA‐TMS)_2_]^2+^** support these assignments (Figure 2), demonstrating short H‐bonding contacts (81–92% of VDW radii)^[^ ^65^ ^]^ between donor protons and acceptor nitrogen atoms.

**Figure 3:**
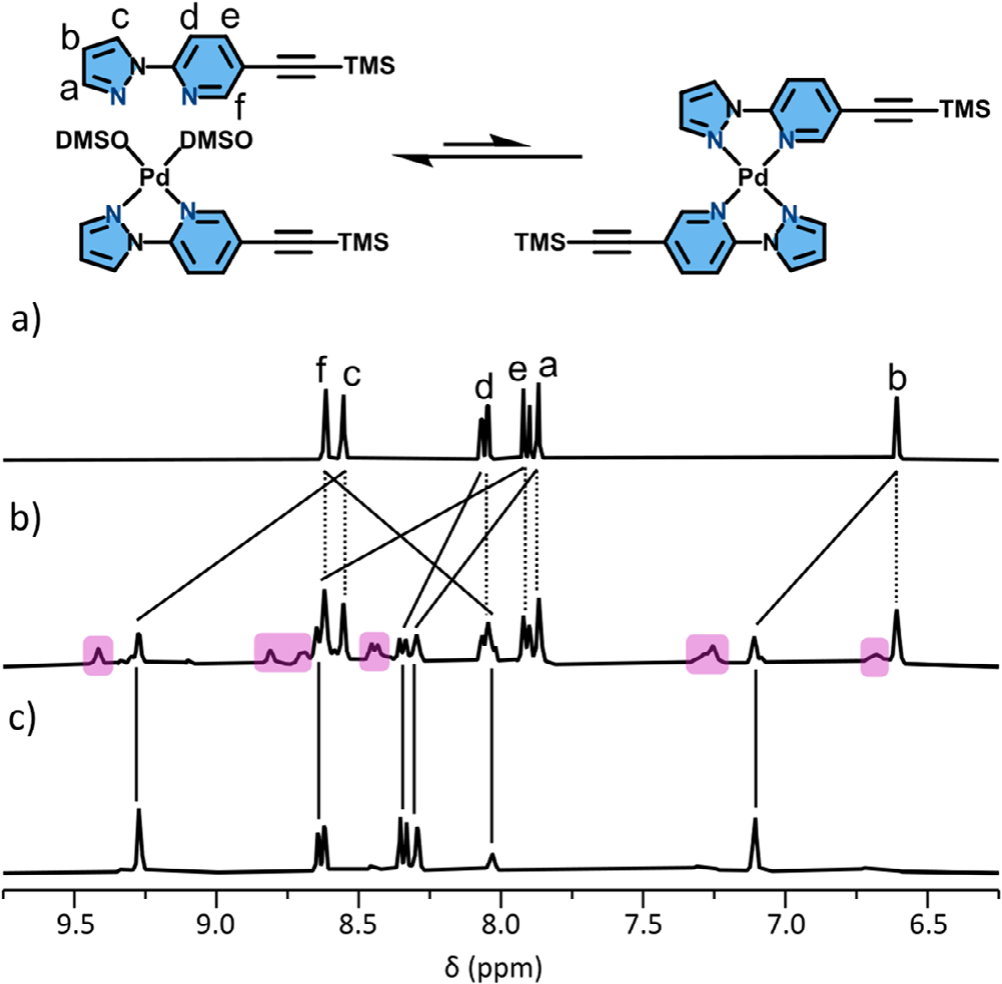
Reaction of DD‐TMS with Pd(II), and partial stacked ^1^H NMR spectra ([D_6_]DMSO, 400 MHz, 298 K) of a) ligand DD‐TMS; b) DD‐TMS with 0.5 eq. Pd(II); and c) DD‐TMS with 1.0 eq. Pd(II). Pink highlights a likely [Pd(DD‐TMS)_2_]^2+^ species.

In contrast, the ligands **DD‐TMS** and **AA‐TMS** possess no H‐bonding **D‐A** complementarity in their homoleptic complexes across the coordination domain, and the outcome of complexation is not readily predicted. Combining Pd(II) with 2 equiv. of **DD‐TMS** gives the free ligand and two new complexes in a 5:3:2 ratio, determined by ^1^H NMR spectroscopy (Figure 3b). Adding further Pd(II) to 1.0 eq. sees full conversion to a single complex that is consistent with **[Pd(DD‐TMS)(DMSO)_2_]^2+^** (Figure 3c). From these data it appears some amount of the M_1_L_2_ complex forms at a M:L ratio of < 1:1 (assigned as HT from calculations, vide infra), which is lost in favor of **[Pd(DD‐TMS)(DMSO)_2_]^2^** ^+^ when Pd(II) is increased to 1.0 equivalent.

**Figure 4:**
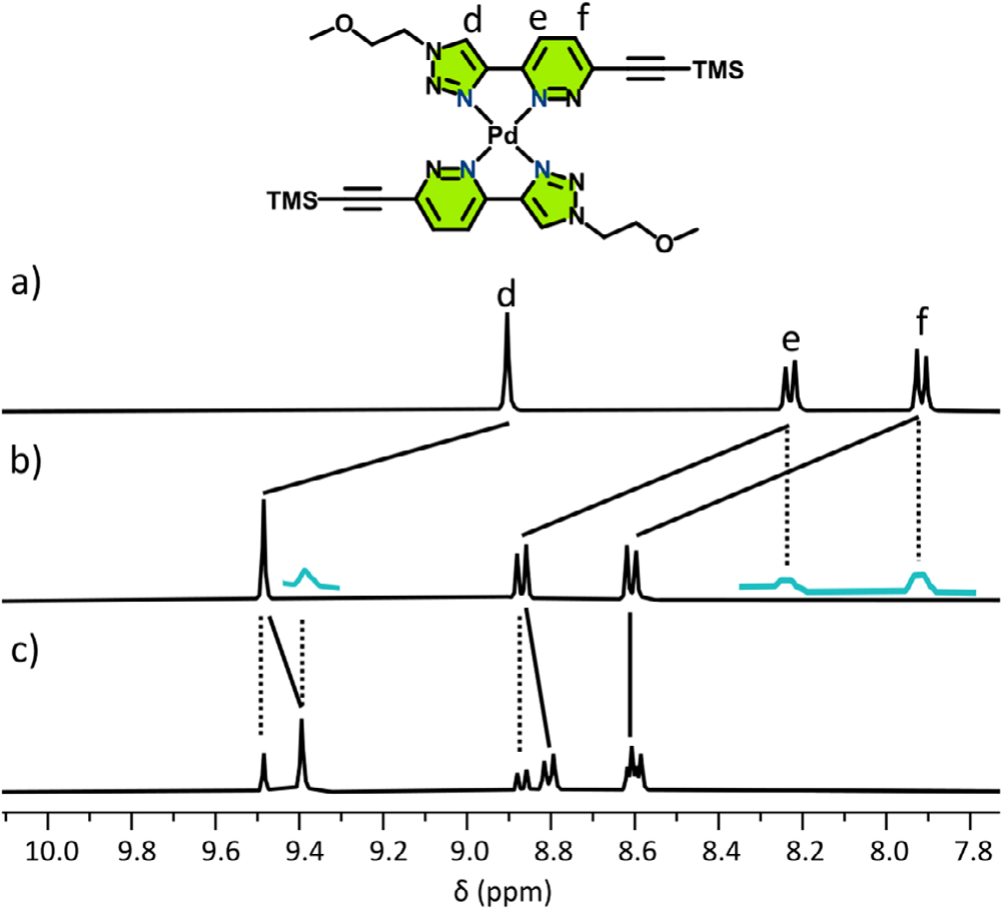
Partial stacked ^1^H NMR spectra ([D_6_]DMSO, 400 MHz, 298 K) of a) ligand AA‐TMS; b) AA‐TMS with 0.5 eq. Pd(II); and c) AA‐TMS with 1.0 eq. Pd(II). Blue inset spectra show expansion of the baseline.

The ligand **AA‐TMS** forms the M_1_L_2_ complex **[Pd(AA‐TMS)_2_]^2+^** assigned as the HT isomer (vide infra for details) in equilibrium with **[Pd(AA‐TMS)(DMSO)_2_]^2+^** (1:0.2 ratio; Figure 4). The further addition of Pd(II) to 1.0 eq. sees **[Pd(AA‐TMS)(DMSO)_2_]^2+^** become the dominant species (ratio 3.3:1; cf. statistical 2:1), indicating there is an enthalpic penalty to M_1_L_2_‐complex formation, although not to the extent of **[Pd(DD‐TMS)(DMSO)_2_]^2^** ^+^, which is formed quantitatively.

**Scheme 2:**
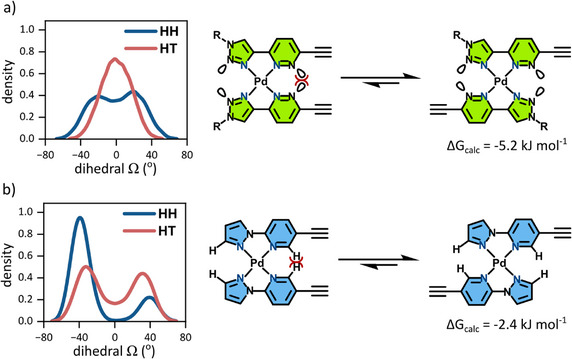
Simulated density of dihedral angles between ligands for HH and HT complexes of a) [Pd(AA‐H)_2_]^2+^ and b) [Pd(AA‐H)_2_]^2+^. High‐energy steric interactions are highlight in red.

For complexes of the **AA** and **DD** ligands, molecular‐dynamics simulations (GFN2‐xTB)^[^ ^66^ ^]^ monitoring the distribution of dihedral angles (Ω) across the metal centre—and hence the degree of distortion on the square‐planar geometry of the metal ion—explain the isomer preferences observed (Scheme 2). The HH complex of **[Pd(AA‐H)_2_]^2+^** demonstrates a bimodal distribution of angles with maxima at ca −20 and 20°, whereas the HT isomer is monomodal with a maximum at 0° (Scheme 2a). These behaviors are rationalized by electrostatic repulsion between N‐lone pairs across the coordination domain; the pyridazinyl‐N lone pairs of the HH isomer are close enough to clash, favoring the HT isomer by *ΔG*_calc_ = −5.2 kJ mol.^−^1 Similarly, both the HH and HT isomers of **[Pd(DD‐H)_2_]^2+^** are destabilized by steric interactions between protons across the coordination domain. Both isomers demonstrate bimodal distributions of dihedral angles (Scheme 2b), with *ΔG*_calc_ favoring the HT isomer by −2.4 kJ mol^−1^. Our conclusions from these calculations and the experimental evidence is that any general **[Pd(DD)_2_]^2+^** system resists both HH and HT complexes in favor of M_1_L_1_, as neither configuration can exist without distortion of ligand dihedral angles. In contrast, complexes of the type **[Pd(AA)_2_]^2+^** prefer the HT configuration. Both behaviors predicted by the calculations are manifest in this study.

**Figure 5:**
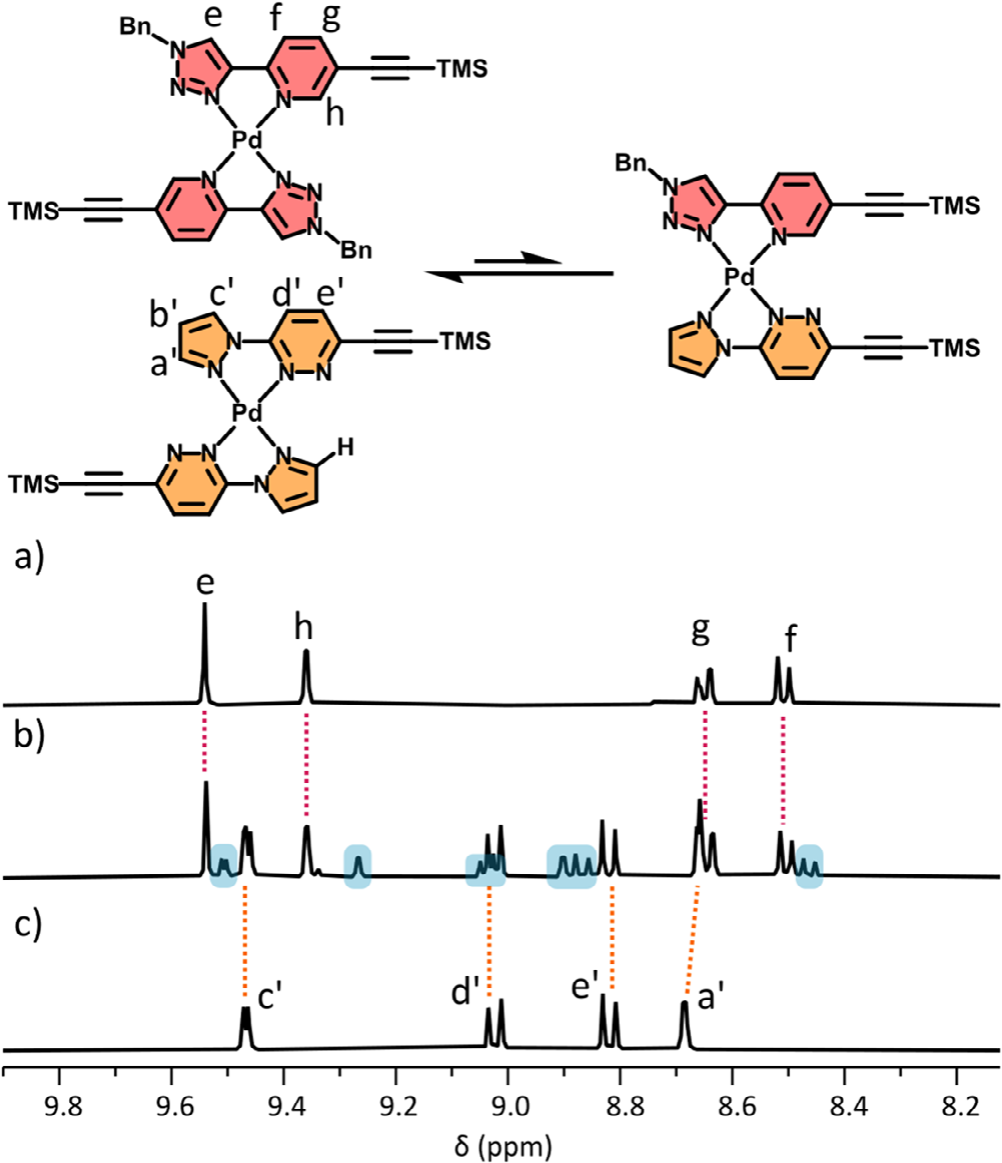
Equilibrium mixture formed when ligands AD‐TMS and DA‐TMS are combined with Pd(II), and partial stacked ^1^H NMR spectra ([D_6_]DMSO, 400 MHz, 298 K) of a) [Pd(AD‐TMS)_2_]^2+^; b) equimolar mixture of [Pd(AD‐TMS)_2_]^2+^ and [Pd(DA‐TMS)_2_]^2+^; and c) [Pd(DA‐TMS)_2_]^2+^. Blue highlights new heteroleptic species [Pd(AD‐TMS)(DA‐TMS)]^2+^.

To assess heteroleptic complex formation in our model system, Pd(II) and the relevant ligands were combined in a 1:1:1 ratio in [D_6_]DMSO. Ligands **AD‐TMS** and **DA‐TMS** gave the complexes **[Pd(AD‐TMS)(DA‐TMS)]^2+^**, **[Pd(AD‐TMS)_2_]^2+^** and **[Pd(DA‐TMS)_2_]^2+^** in a 0.8:1:1 ratio, determined by ^1^H NMR spectroscopy (cf. statistical ratio 2:1:1), and observed by HR‐MS (Figure 5). The difference between the observed and statistical ratio of products suggests the heteroleptic complex **[Pd(AD‐TMS)(DA‐TMS)]^2+^** is slightly disfavored. Computational analysis indicates this species is likely the HH isomer (*ΔG*_calc_ = −6.0 kJ mol^−1^; *K*_calc _= 11.3).

Combining **AA‐TMS** and **DD‐TMS** gave two new **[Pd(AA‐TMS)(DD‐TMS)]^2+^** species in a 4:1 ratio, that could not be assigned as HH or HT experimentally; however, calculated energies for the complexes showed a preference toward HT (*ΔG*_calc_ = −9.5 kJ mol^−1^; *K*_calc _= 47). HR‐MS further supported heteroleptic‐complex formation, although minor amounts of the homoleptic species were seen due to the sensitivity of this technique. The apparent absence of homoleptic species is driven by the formation of enthalpically favorable AA:DD interactions, that are not possible for the homoleptic complexes and are inherently less stable.

These model complexes demonstrate how complementary H‐bonding interactions in the coordination domain of M_1_L_2_ complexes have limited control over the geometric outcome. Of the six complexes assessed, only **[Pd(AD‐TMS)_2_]^2+^** and **[Pd(DA‐TMS)_2_]^2+^** form cleanly due to unambiguous HT donor‐acceptor complementarity in the ligand pairings. Ligands **AA‐TMS** and **DD‐TMS** each form some amount of M_1_L_2_ homoleptic complexes, but due to enthalpically costly LP···LP or H···H steric clashing across the coordination sphere, the **[Pd(L)(DMSO)_2_]^2+^** complexes are favored when excess Pd(II) is added. Likewise, the heteroleptic complexes **[Pd(AD‐TMS)(DA‐TMS)]^2+^** and **[Pd(AA‐TMS)(DD‐TMS)]^2+^** are only formed as mixtures of isomers and/or homoleptic complexes, as there is insufficient enthalpic driving force to favor a single compound.

### 2.3 The Recognition Domain: Homoleptic Complexes

With an understanding of how the complexation domain influences isomer formation, we next studied the effect of the recognition domains ER and EP on homoleptic complex formation. The species were analyzed as above described earlier, and using ^19^F NMR spectroscopy where appropriate.

The homoleptic complexes **[Pd(ADER)_2_]^2+^**, **[Pd(DAER)_2_]^2+^**, and **[Pd(ADEP)_2_]^2+^** were each formed as the sole major species in the ^1^H NMR spectra. The complex **[Pd(ADEP)_2_]^2+^** was successfully crystallized as the HT isomer; this behavior was expected as only this orientation sees complementary H‐bonding across the coordination domain. The complexes **[Pd(ADER)_2_]^2+^** and **[Pd(DAER)_2_]^2+^** were similarly assigned as HT. When further Pd(II) was added to each sample (to 1.0 eq.), the new species **[Pd(L)(DMSO)_2_]^2+^** were formed in equilibrium with the M_1_L_2_ complexes as minor components (<50%), compared to the AA and DD ligands which strongly favor M_1_L_1_ complexes (>78%).

Similar to **DD‐TMS**, the ligands **DDER** and **DDEP** did not form significant amounts of their homoleptic M_1_L_2_ complexes, due to the steric demand of the donor protons in the coordination domain. Small amounts of **[Pd(DDER)_2_]^2+^** are formed, while the species **[Pd(DDEP)_2_]^2+^** is not observed, instead giving only free ligand and **[Pd(DDEP)(DMSO)_2_]^2+^**, which forms quantitatively when 1.0 eq. of Pd(II) is added.

**Figure 6:**
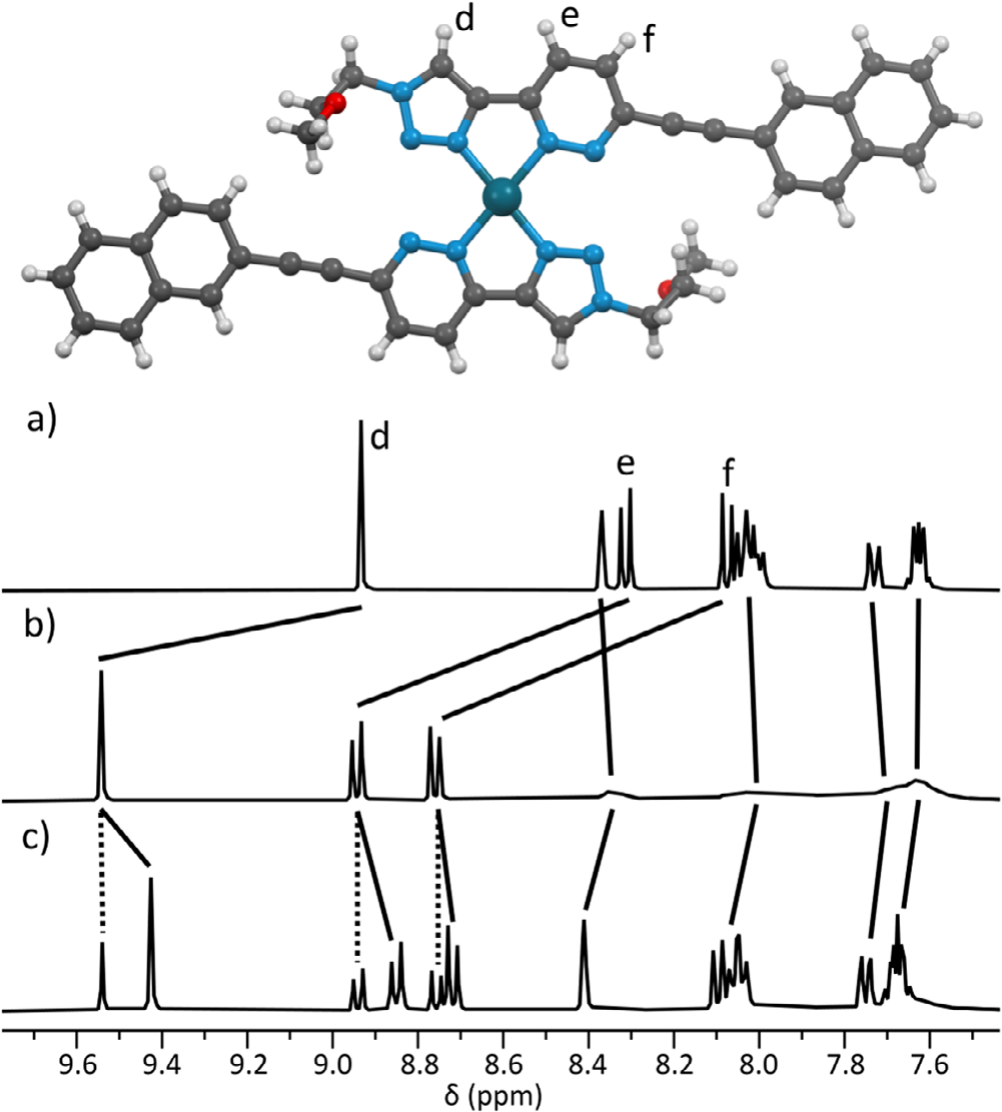
X‐ray crystal structure of [Pd(AAER)_2_](BF_4_)_2_ (counterions omitted for clarity), and partial ^1^H NMR spectra ([D_6_]DMSO, 400 MHz, 298 K) showing a) ligand AAER; b) with 0.5 eq. Pd(II) and c) with 1.0 eq. Pd(II).

The major species **[Pd(AAER)_2_]^2+^** and **[Pd(AAEP)_2_]^2+^** formed from **AAER** and **AAEP**, respectively, with trace amounts of **[Pd(L)(DMSO)_2_]^2+^** and free ligand in each case observed in the ^1^H NMR spectra (see SI; Figures S55, S60). The ^1^H NMR spectrum of **[Pd(AAER)_2_]^2+^** shows significant broadening of the naphthalene resonances, that at first was attributed to intramolecular π···π interactions that are slow on the NMR timescale, and only possible in the HH configuration (Figure 6b). However, the chemical shifts of these signals were unchanged which is inconsistent with π‐π‐stacking, and the addition of further Pd(II) to 1.0 eq. shifted the ratio to 4.3:1 favoring **[Pd(AAER)(DMSO)_2_]^2+^**; a similar ratio as for the **AA‐TMS** (3.4:1) and **AAEP** (3.4:1) ligands. These data signal there is little enthalpic stabilization arising from π‐π interactions between the ER naphthalene fragments, consistent with the HT isomer. We eventually obtained a SCXRD structure of **[Pd(AAER)_2_]^2+^** revealing the HT isomer (Figure 6), and no meaningful dihedral distortion between adjacent ligands, consistent with the computational analysis in Scheme 2a. The cause of the broadening of the naphthalene signals in the ^1^H NMR spectrum appears to be due to intermolecular π‐π‐stacking, as dilution experiments showed the broadening is reduced at low concentrations (see SI; Figure S59). On the basis of similarity we assigned the HT isomer for the other AA_2_ complexes **[Pd(AAER)_2_]^2+^**, **[Pd(AAEP)_2_]^2+^** and **[Pd(AA‐TMS)_2_]^2+^**.

With the exception of **[Pd(AAER)_2_]^2+^**, calculated energies (GFN2‐xTB)^[^ ^66^ ^]^ for configurational isomers of a selection of complexes were consistent with the experimental assignments (Table 2). The species **[Pd(ADER)_2_]^2+^** and **[Pd(DAER)_2_]^2+^** strongly favor the HT isomers by *ΔG*_calc_ = ‒25.1 and ‒10.2 kJ mol^−1^, respectively, as do the model complexes **[Pd(AA‐TMS)_2_]^2+^** and **[Pd(AA‐H)_2_]^2+^** (*ΔG*_calc_ = ‒15.2 and ‒5.2 kJ mol^−1^, respectively). However, xTB calculations show **[Pd(AAER)_2_]^2+^** prefers the HH isomer by a significant margin (*ΔG*_calc_ = ‒10.1 kJ mol^−1^); an amount that increases using DFT methods (r^2^SCAN‐3c/CPCM(DMSO);^[^ ^67^, ^68^ ^]^ *ΔG*_calc_ = ‒16.7 kJ mol^−1^). It appears these methods overstate the effect of the intermolecular, electron‐rich π‐π stacking interactions in this complex. Notably, xTB calculations for the analogous electron‐poor species **[Pd(AAEP)_2_]^2+^** predict the HT isomer is favored, as is expected based on the crystal structure of **[Pd(AAER)_2_]^2+^**.

**Table 2:** Summary of calculated energies for selected complex isomers. For [Pd(AAER)_2_]^2+^ these diverge from experimental results.

complex	method	major isomer	*ΔG*_calc_ (kJ mol^−1^)	HT;HH ratio
**[Pd(ADER)_2_]^2+^**	xTB	HT	‒25.1	>99:1
**[Pd(DAER)_2_]^2+^**	xTB	HT	‒10.2	2:98
**[Pd(AA‐TMS)_2_]^2+^**	xTB	HT	‒15.2	>99:1
**[Pd(AA‐H)_2_]^2+^**	xTB	HT	‒5.2	89:11
	DFT	HT	‒16.7	80:20
**[Pd(AAER)_2_]^2+^**	xTB	HH	‒10.1	2:98
	DFT	HH	‒16.7	<1:99
**[Pd(AAEP)_2_]^2+^**	xTB	HT	−6.1	92:8

Broadly, trends in the preceding data follow those of the homoleptic model complexes, with HT isomers being the dominant species. Importantly, there is little to suggest that the **ER** or **EP** recognition domains meaningfully influence the ratios or geometries of homoleptic species—a key role of these functional groups if they are to promote orthogonal heteroleptic complex formation.

### 2.4 The Recognition Domain: Heteroleptic Complexes

From the seven ligands, three orthogonal heteroleptic complexes are possible: **[Pd(AAEP)(DDER)]^2+^**, **[Pd(AAER)(DDEP)]^2+^** and **[Pd(ADEP)(DAER)]^2+^**. Their formation was assessed by combining [D_6_]DMSO solutions of the respective homoleptic complexes in an equimolar ratio, and the mixtures analyzed as previously discussed. Due to the sensitivity of the technique, in some cases HR‐MS showed small amounts of the homoleptic species that were not detected by ^1^H NMR spectroscopy.

**Figure 7:**
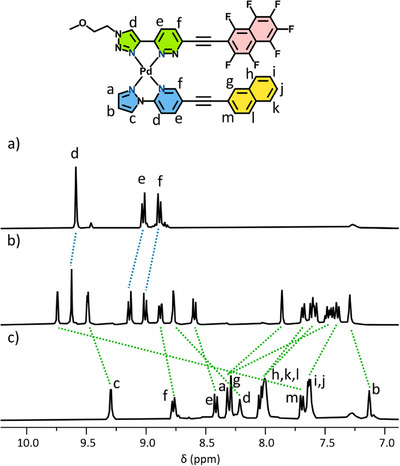
The structure of [Pd(AAEP)(DDER)]^2+^, and Partial ^1^H NMR spectra ([D_6_]DMSO, 400 MHz, 298 K) showing a) [Pd(AAEP)_2_]^2+^; b) [Pd(AAEP)(DDER)]^2+^; and c) [Pd(DDEP)(DMSO)_2_]^2+^.

The ^1^H NMR spectra of **[Pd(AAEP)(DDER)]^2+^** and **[Pd(AAER)(DDEP)]^2+^** were remarkably clean, each revealing only a single species with no evidence of their homoleptic components or HH/HT isomerism. The ^1^H NMR spectra of **[Pd(AAEP)(DDER)]^2+^** and the homoleptic complexes are shown in Figure 7. In both cases, the heteroleptic complexes were the major species in the HR‐MS spectra (see SI; Figures S94, S99). The formation of single heteroleptic species contrasts with the model complex **[Pd(AA‐TMS)(DD‐TMS)_2_]^2+^**, where a mixture of HH and HT complexes were observed. Both **[Pd(AAEP)(DDER)]^2+^** and **[Pd(AAER)(DDEP)]^2+^** were assigned as HH due to the marked upfield shift of the naphthalene proton resonances, consistent with the shielding effect afforded by π‐π‐stacking interactions. The ^19^F NMR resonances are largely unshifted between ligands, homoleptic (HT) and heteroleptic (HH) complexes, and so could not be used diagnostically in this study.

A single heteroleptic complex of **[Pd(ADEP)(DAER)]^2+^** was formed in equilibrium with the homoleptic complexes in a 9:1:1 ratio (cf. statistical ratio 2:1:1), and the heteroleptic complex was the major component of the HR‐MS spectrum. While the heteroleptic selectivity is not total, this outcome compares favorably with the model complex **[Pd(AD‐TMS)(DA‐TMS)]^2+^,** where the ratio was 5:6:6. Again, the complex was assigned as HH due to significant upfield shifts (0.3–0.5 ppm) of the naphthalene resonances in the ^1^H NMR spectrum. When compared to **Pd(AAEP)(DDER)]^2+^** and **[Pd(AAER)(DDEP)]^2+^,** the reduced selectivity toward **[Pd(ADEP)(DAER)]^2+^** likely arises from the favorable H‐bonding interactions present in both homoleptic and heteroleptic complexes.

To further probe the influence on orthogonality of the recognition domain, the ER ligands **ADER** and **DAER** were combined with Pd(II) in a 1:1:1 ratio. With no EP domain to pair with, a mixture of **[Pd(ADER)(DAER)]^2+^, [Pd(ADER)_2_]^2+^** and **[Pd(DAER)_2_]^2+^** was formed in a 0.8:1:1 ratio (cf. statistical ratio 2:1:1). In principle, the two ER domains can form favorable off‐set face‐to‐face π‐π‐stacking interactions; however, from these data it is apparent that the interactions are insufficient to drive complete heteroleptic complexation, due to weak enthalpic stabilization and/or the inability of the units to attain a favorable π‐π‐stacking geometry. Similarly, the complex **[Pd(AAER)(AAEP)]^2+^**—which has complementary recognition fragments but lacks complementary H‐bonding in the coordination domain—fails to form cleanly. From the HR‐MS and ^1^H NMR data (see SI; Figures S103‐104), it appears the heteroleptic complex is favored over the homoleptic complexes **[Pd(AAER)_2_]^2+^** and **[Pd(AAEP)_2_]^2+^** by a small margin (ratio 4:1:1; cf. statistical 2:1:1).

These experiments demonstrate the importance of favorable interactions in both domains for controlling isomer distribution in this system. The heteroleptic complexes **[Pd(AAEP)(DDER)]^2+^**, **[Pd(AAER)(DDEP)]^2+^** and **[Pd(ADEP)(DAER)]^2+^** are formed with high selectivity (>90%), compared to species lacking favorable interactions in the coordination or recognition domains.

### 2.5 Combinatorial Studies

Discounting HH/HT isomerism, there are 28 unique homoleptic and heteroleptic complexes that can be formed from the seven ligands in this study. To assess the orthogonality of the targeted complexes, pairs of the heteroleptic complexes were combined in a 1:1 mol ratio in [D_6_]DMSO and the mixture analyzed by ^1^H NMR spectroscopy. HR‐MS spectra were also collected; however, this technique is unable to distinguish mixed species (for example, **[Pd(AAER)(DDEP)]^2+^** and **[Pd(DDER)(AAEP)]^2+^**) and so was used with caution.

Of the three pairs, only **[Pd(AAER)(DDEP)]^2+^** and **[Pd(ADEP)(DAER)]^2+^** showed measurable ligand exchange, and only as a minor component (ca 10%; see SI). The new species is likely **[Pd(AAER)(ADEP)]^2+^**, which is found in the HR‐MS spectrum. Further, only this combination of ligands can form some amount of favorable interactions in both the coordination and recognition domains—**[Pd(DDEP)(DAER)]^2+^** would result in steric clashing between donor protons and therefore dihedral‐angle distortion (vide supra).

The other combinations **[Pd(AAEP)(DDER)]^2+^** and **[Pd(AAER)(DDEP)]^2+^**, and **[Pd(AAEP)(DDER)]^2+^** and **[Pd(ADEP)(DAER)]^2+^** showed no evidence of scrambling in the ^1^H NMR spectra. HR‐MS did show scrambled complexes as minor components, demonstrating the sensitivity of this technique compared to NMR.

**Figure 8:**
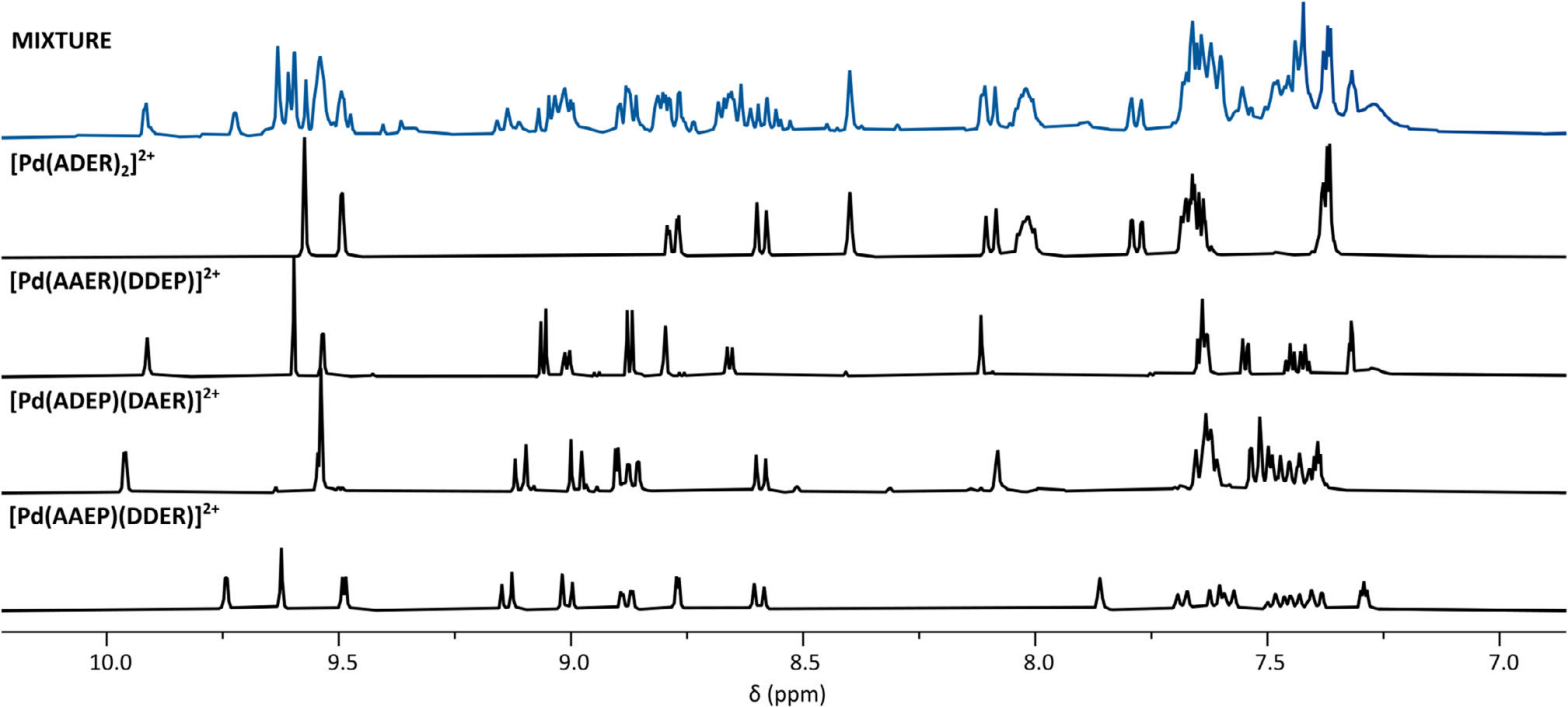
Partial ^1^H NMR spectra ([D_6_]DMSO, 400 MHz, 298 K) of the combination of all seven homoleptic complexes (top), and the four species present.

When all seven homoleptic species were combined in an equimolar ratio, the resulting ^1^H NMR spectrum revealed the three heteroleptic complexes **[Pd(AAEP)(DDER)]^2+^**, **[Pd(AAER)(DDEP)]^2+^** and **[Pd(ADEP)(DAER)]^2+^** were formed, with **[Pd(ADER)_2_]^2+^**—which has no partner for heteroleptic complexation—unchanged (Figure 8). The addition of further aliquots of Pd(II) up to 0.5 eq. saw only the formation of **Pd(ADER)(DMSO)_2_]^2+^,** as **[Pd(ADER)_2_]^2+^** lacks a ligand partner and is not stabilized by the recognition domain. This experiment demonstrates the stability of the heteroleptic complexes toward perturbation, as all homoleptic complexes formed some amount of **[Pd(L)(DMSO)_2_]^2+^** when additional Pd(II) was added.

## 3 Conclusion

In summary, we designed and synthesized a series of ligands bearing two sites of interaction: the coordination and recognition domains. Studies forming homo‐ and heteroleptic complexes from model ligands with no recognition domain showed that the coordination domain alone is insufficient for isomer selectivity with high fidelity. Similarly, **ER**:**EP** ligand pairings that cannot interact in the coordination domain (such as **[Pd(AAER)(AAEP)]^2+^**) formed mixtures of isomers. Only when both domains are exploited in tandem does a single species form, with each of the target heteroleptic complexes **[Pd(AAEP)(DDER)]^2+^**, **[Pd(AAER)(DDEP)]^2+^** and **[Pd(ADEP)(DAER)]^2+^** formed with > 90% selectivity. Further, combinatorial studies show the three heteroleptic complexes themselves may exist orthogonally, paving the way for use in the preparation of larger and more complex assemblies.

## Supporting Information

The authors have cited additional references within the Supporting Information.^[^ ^69^, ^70^, ^71^, ^72^, ^73^, ^74^, ^75^, ^76^, ^77^, ^78^, ^79^, ^80^, ^81^, ^82^, ^83^ ^]^

## Conflict of Interest

The authors declare no conflict of interest.

## Supporting information



Supporting Information

## Data Availability

The data that support the findings of this study are available in the supplementary material of this article.
