# Supplementary material for: Coordination‐Driven Orthogonal Ligand Pairings through Dual Hydrogen‐Bonding/π‐π Interaction Complementarity
Source: Chemistry. 2025 Sep 10;31(64):e02411. doi: 10.1002/chem.202502411 (PMC12624320; doi:10.1002/chem.202502411)
Supplement: Supplementary file 1 — Supporting Information [file CHEM-31-e02411-s001.pdf]

<u>Supporting Information</u>

*Jordan N. Smith, Yolanda Yau, Nina R. Lawson, Rosemary J. Goodwin, & Dan Preston**

Research School of Chemistry, The Australian National University, Canberra, ACT 2600, Australia.

***daniel.preston@anu.edu.au**

Contents

1. Synthesis & Characterisation 4
1.1. General 4
1.2. Ligand Synthesis 5
1.2.1. AA-TMS 5
1.2.2. AD-TMS 6
1.2.3. DD-TMS 7
1.2.4. DA-TMS 8
1.2.5. DD-H 9
1.2.6. AD-H 10
1.2.7. DA-H 11
1.2.8. AA-H 12
1.2.9. AAER 13
1.2.10. AAEP 14
1.2.11. ADER 15
1.2.12. ADEP 16
1.2.13. DDER 17
1.2.14. DDEP 18
1.2.15. DAER 19
2. Complexations 21
2.1. General 21
2.2. Equilibria 21
2.3. Model Complexes 23
2.3.1. $[Pd(DA\text{-}TMS)_2](BF_4)_2$ 23
2.3.2. $[Pd(AD\text{-}TMS)_2](BF_4)_2$ 24
2.3.3. $[Pd(AA\text{-}TMS)_2](BF_4)_2$ 25
2.3.4. $[Pd(DD\text{-}TMS)(solvent)_2](BF_4)_2$ 27
2.3.5. Comparison of TMS-protected and terminal-alkyne complexes 29
2.3.6. Combining $[Pd(DA\text{-}TMS)_2](BF_4)_2$ and $[Pd(AD\text{-}TMS)_2](BF_4)_2$ 30
2.3.7. Combining '$[Pd(DD\text{-}TMS)_2](BF_4)_2$' and $[Pd(AA\text{-}TMS)_2](BF_4)_2$ 31
2.4. Homoleptic Complexes 33
2.4.1. $[Pd(AAER)_2](BF_4)_2$ 33
2.4.2. $[Pd(AAEP)_2](BF_4)_2$ 35
2.4.3. $[Pd(ADER)_2](BF_4)_2$ 37

2.4.4. $[Pd(DAER)_2](BF_4)_2$ 40
2.4.5. $[Pd(DDER)_2](BF_4)_2$ 42
2.4.6. $[Pd(DDEP)_2](BF_4)_2$ 44
2.5. Heteroleptic Complexes 46
2.5.1. Combining $[Pd(ADEP)_2](BF_4)_2$ and $[Pd(DAER)_2](BF_4)_2$ 46
2.5.2. Combining $[Pd(AAEP)_2](BF_4)_2$ and $[Pd(DDER)_2](BF_4)_2$ 48
2.5.3. Combining $[Pd(AAER)_2](BF_4)_2$ and $[Pd(DDEP)](BF_4)_2$ 51
2.5.4. Combining $[Pd(DAER)_2](BF_4)_2$ and $[Pd(ADER)_2](BF_4)_2$ 53
2.5.5. Combining $[Pd(AAER)_2](BF_4)_2$ and $[Pd(AAEP)_2](BF_4)_2$ 55
2.6. Scrambling Experiments 57
2.6.1. General 57
2.6.2. Combining $[Pd(AAER)(DDEP)](BF_4)_2$ and $[Pd(AAEP)(DDER)](BF_4)_2$ 57
2.6.3. Combining $[Pd(AAER)(DDEP)](BF_4)_2$ and $[Pd(ADEP)(DAER)](BF_4)_2$ 59
2.6.4. Combining $[Pd(AAEP)(DDER)](BF_4)_2$ and $[Pd(ADEP)(DAER)](BF_4)_2$ 60
2.6.5. Combining all homoleptic species 62
3. Calculations 64
3.1. General 64
3.2. Calculated energies 64
3.3. Molecular dynamics simulations 65
3.3.1. $[Pd(ADER)_2]^{2+}$ and $[Pd(DAER)_2]^{2+}$ 65
3.3.2. $[Pd(AAER)_2]^{2+}$ 68
3.3.3. $[Pd(AAEP)_2]^{2+}$ 68
3.3.4. $[Pd(AA\text{-}H)_2]^{2+}$ and $[Pd(DD\text{-}H)_2]^{2+}$ 69
3.3.5. $[Pd(AD\text{-}TMS)(DA\text{-}TMS)]^{2+}$ 71
4. Crystallography 73
4.1. Experimental data 73
4.2. ORTEP diagrams 75
5. References 77

1. Synthesis & Characterisation

1.1. General

All reagents were purchased from commercial sources and used without further purification. 3,6-Bis((trimethylsilyl)ethynyl)pyridazine,[69] 5-bromo-2-(trimethylsilyl)ethynyl)pyridine,[70] and 2-(1-benzyl-1*H*-1,2,3-triazol-4-yl)-5-bromopyridine[71] were synthesised according the literature procedures with minor modifications. Solvents were laboratory reagent grade. Abbreviations: dichloromethane (DCM), ethylenediaminetetraacetate (EDTA), tetrahydrofuran (THF), dimethyl sulfoxide (DMSO), dimethylformamide (DMF). ^{1}H, ^{13}C, ^{19}F and DOSY NMR spectra were recorded on a Bruker Avance 400 MHz spectrometer. Chemical shifts are reported in parts per million and referenced to residual solvent peaks ($CDCl_3$: ^{1}H δ 7.26 ppm, ^{13}C δ 77.16 ppm; [D_6]DMSO: ^{1}H δ 2.50 ppm; ^{13}C δ 39.52 ppm). Coupling constants (*J*) are reported in Hertz (Hz). ^{19}F spectra are unreferenced. Standard abbreviations indicating multiplicity were used as follows: m = multiplet, q = quartet, quin = quintet, t = triplet, dt = double triplet, d = doublet, dd = double doublet, s = singlet, br = broad. High Resolution Electrospray Mass Spectra (HR ESI-MS) were collected on a Waters Synapt G2-S1 HDMS spectrometer or an Orbitrap Elite spectrometer, and High Resolution Nanospray Mass Spectra (Nanospray MS) were collected on an Orbitrap Elite spectrometer.

CAUTION: WHILE NO PROBLEMS WERE ENCOUNTERED DURING THIS WORK, AZIDES ARE EXPLOSIVE AND CARE SHOULD BE TAKEN WHEN DEALING WITH THEM.

1.2. Ligand Synthesis

1.2.1. AA-TMS

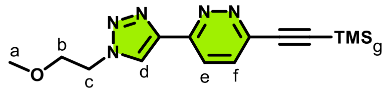

1-Iodo-2-methoxyethane (51 mg, 0.28 mmol) and sodium azide (18 mg, 0.28 mmol) were heated for 1.5 hours at 110 °C in DMF (20 mL). After cooling to rt, sodium carbonate (19 mg, 0.18 mmol) and 3,6-bis((trimethylsilyl)ethynyl)pyridazine (154 mg, 0.57 mmol) were added to the reaction mixture. Then, copper(II) sulfate pentahydrate (15 mg, 0.092 mmol) and sodium ascorbate (36 mg, 0.18 mmol) were added. The reaction mixture was left to stir for 17 hours under a nitrogen atmosphere at rt. Afterwards, 0.1 M EDTA/NH_4OH (aq.; 20 mL) and DCM (20 mL) were added, and the mixture was stirred vigorously for 5 min. The organic layer was then washed with distilled water (5 × 20 mL) and the volatiles removed in vacuo. The residue was purified via column chromatography (SiO_2, 5% acetone/DCM) and the volatiles removed in vacuo affording **AA-TMS** as a brown solid (42 mg, 0.14 mmol, 50%). ^1H NMR (400 MHz, $CDCl_3$, 298 K) *δ*: 8.54 (s, 1H, H_d), 8.24 (d, *J* = 8.8 Hz, 1H, H_e), 7.64 (d, *J* = 8.8 Hz, 1H, H_f), 4.63 (t, *J* = 5.0 Hz, 2H, H_c), 3.81 (t, *J* = 5.0 Hz, 2H, H_b), 3.36 (s, 3H, H_a), 0.29 (s, 9H, H_g). ^{13}C NMR (100 MHz, $CDCl_3$, 298 K) *δ*: 151.6, 146.7, 145.0, 130.5, 124.5, 122.7, 100.9, 100.8, 70.6, 59.2, 50.7, -0.3. HR ESI-MS ($CHCl_3$/MeOH) *m/z* = 302.1440 $[M+H]^+$ (calcd for $C_{14}H_{20}N_5O_{28}Si$, 302.1432).

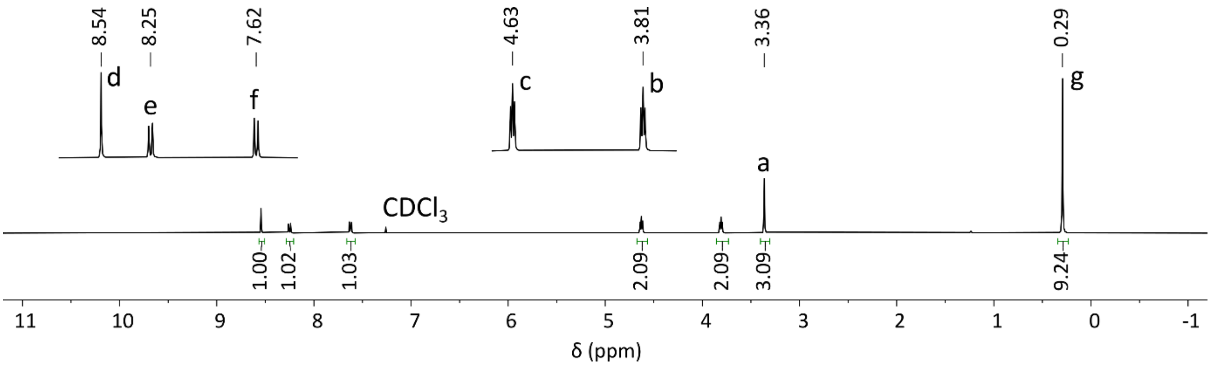

Figure S1. ^1H NMR ($CDCl_3$, 400 MHz, 298K) spectrum of **AA-TMS**.

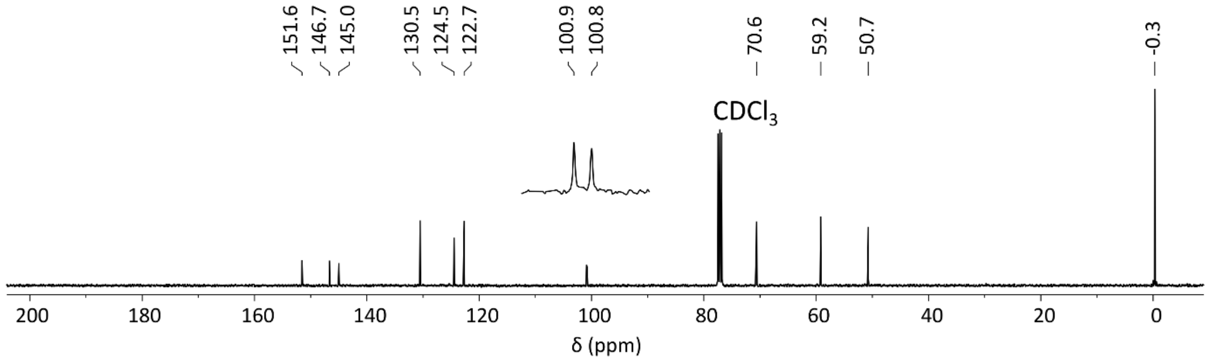

Figure S2. ^{13}C NMR ($CDCl_3$, 100 MHz, 298K) spectrum of **AA-TMS**.

1.2.2. AD-TMS

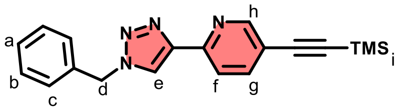

Trimethylsilylacetylene (662 mg, 6.74 mmol), dichlorobis(triphenylphospine)palladium(II) (114 mg, 0.16 mmol), and copper iodide (35 mg, 0.18 mmol) were added to a deoxygenated mixture of compound 2-(1-benzyl-1*H*-1,2,3-triazol-4-yl)-5-bromopyridine (569 mg, 1.80 mmol) in 3:5 triethylamine/dioxane (4 mL) in a heavy-walled pressure tube. The mixture was then stirred at 80 °C for 17 h. 0.1 M EDTA/NH_4OH (aq.; 20 mL) and DCM (20 mL) were added, and the mixture was stirred vigorously for 5 min. The organic layer was then washed with distilled water (2 × 20 mL) and the volatiles removed in vacuo. The residue was purified via column chromatography (SiO_2; 10% acetone/DCM) and the volatiles removed in vacuo affording **AD-TMS** as a light brown solid (551 mg, 1.66 mmol, 92%). ^{1}H NMR (400 MHz, $CDCl_3$, 298 K) δ: 8.59 (dd, *J* = 2.1, 1.0 Hz, 1H, H_h), 8.11 (dd, *J* = 8.2, 0.9 Hz, 1H, H_f), 8.04 (s, 1H, H_e), 7.69 (dd, *J* = 8.2, 2.1 Hz 1H, H_g), 7.37 (m, 5H $H_{a,b,c}$), 5.58 (s, 1H, H_d), 0.27 (s, 9H, H_i). ^{13}C NMR (100 MHz, $CDCl_3$, 298 K) δ: 152.3, 149.2, 148.4, 139.9, 134.4, 129.3, 129.0, 128.4, 122.5, 119.4, 119.3, 101.8, 98.7, 54.5, 0.0. HR ESI-MS ($CHCl_3$/MeOH) *m/z* = 355.1347 [M + Na]$^+$ (calcd for $C_{19}H_{20}N_4SiNa$, 355.1349).

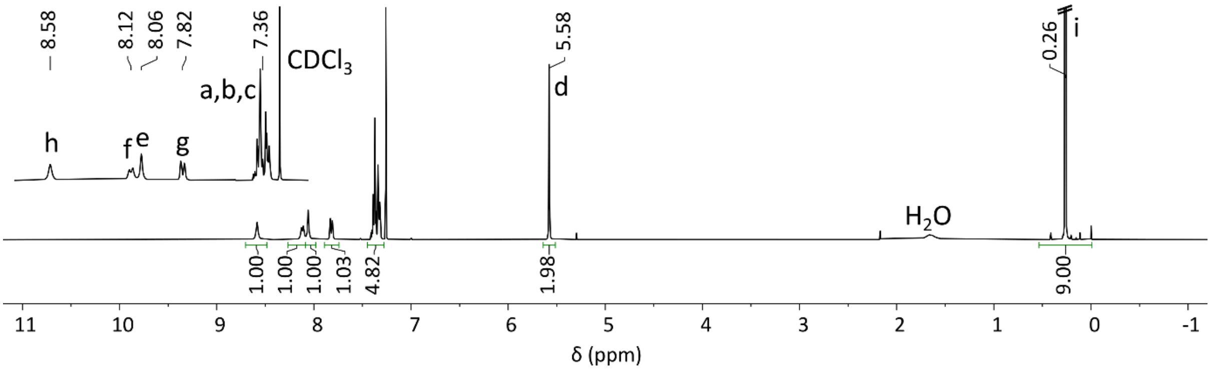

Figure S3. ^{1}H NMR ($CDCl_3$, 400 MHz, 298K) spectrum of **AD-TMS**.

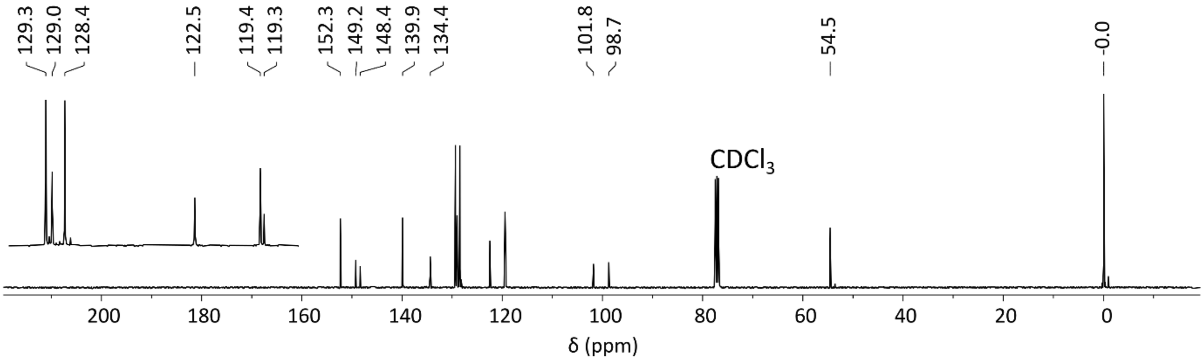

Figure S4. ^{13}C NMR ($CDCl_3$, 100 MHz, 298K) spectrum of **AD-TMS**.

1.2.3. DD-TMS

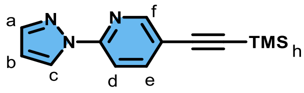

Trimethylsilylacetylene (2.03 g, 20.7 mmol), dichlorobis(triphenylphospine)palladium(II) (326 mg, 0.47 mmol), and copper iodide (178 mg, 0.94 mmol) were added to a deoxygenated mixture of 5-bromo-2-(1*H*-pyrazol-1-yl)pyridine (2.28 g, 10.2 mmol) in 1:1 triethylamine/THF (100 mL). The mixture was then stirred at 50 °C for 17 hours under a nitrogen atmosphere. 0.1 M EDTA/NH_4OH (aq.; 20 mL) and DCM (20 mL) were added and the mixture was stirred vigorously for 30 min. The organic layer was then washed with distilled water (3 × 20 mL) and the volatiles removed in vacuo. The residue was purified via column chromatography (SiO_2, 1:1 DCM/PET) and the volatiles removed in vacuo affording **DD-TMS** as a white solid (1.90 g, 9.32 mmol, 91%). ^1H NMR (400 MHz, $CDCl_3$, 298 K) δ: 8.54 (d, J = 2.5 Hz, 1H, H_f), 8.47 (s, 1H, H_c), 7.94 (d, J = 8.6 Hz, 1H, H_d), 7.85 (dd, J = 8.6, 2.5 Hz 1H, H_e), 7.74 (s, 1H H_a), 6.48 (s, 1H, H_b), 0.27 (s, 9H, H_h). ^{13}C NMR (100 MHz, $CDCl_3$, 298 K) δ: 151.3, 150.4, 142.6, 141.8, 127.5, 117.9, 111.8, 108.3, 101.3, 98.3, 0.0. HR ESI-MS ($CHCl_3$/CH_3CN) m/z = 242.1120 $[M+H]^+$ (calcd for $C_{13}N_3SiH_{15}$, 242.1113).

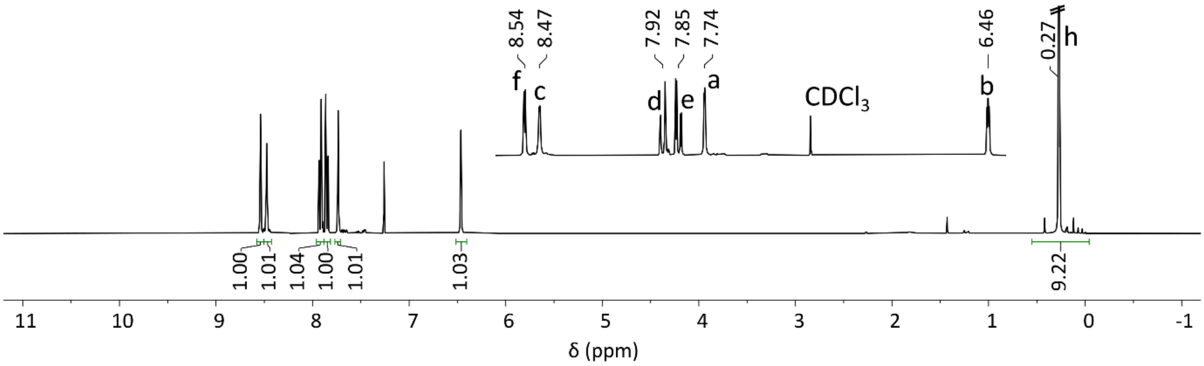

Figure S5. ^1H NMR ($CDCl_3$, 400 MHz, 298K) spectrum of **DD-TMS**.

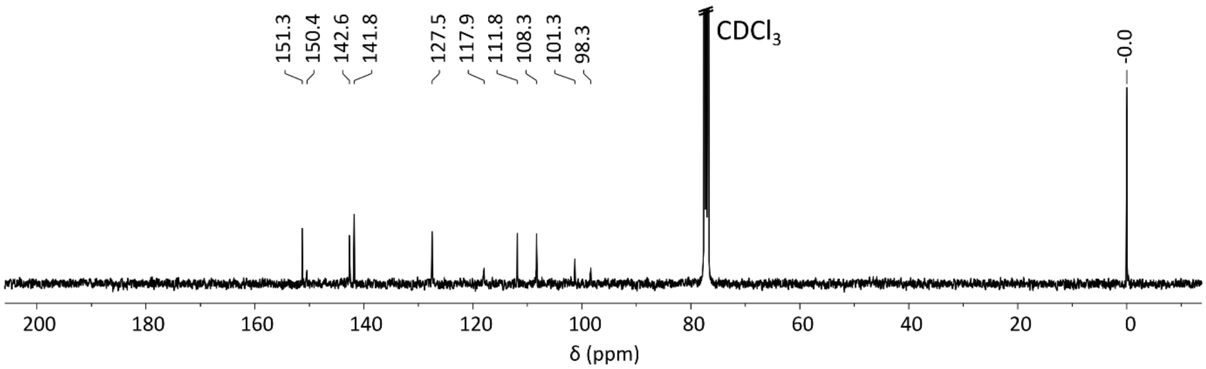

Figure S6. ^{13}C NMR ($CDCl_3$, 100 MHz, 298K) spectrum of **DD-TMS**.

1.2.4. DA-TMS

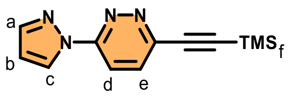

Trimethylsilylacetylene (288 mg, 2.93 mmol), dichlorobis(triphenylphospine)palladium(II) (64 mg, 0.091 mmol), and copper iodide (40 mg, 0.21 mmol) were added to a deoxygenated mixture of 3-bromo-6-(1*H*-pyrazol-1-yl)pyridazine (410 mg, 1.82 mmol) in 1:1 triethylamine/THF (20 mL). The mixture was then stirred at 50 °C for 40 hours under a nitrogen atmosphere. 0.1 M EDTA/NH_4OH (aq.; 20 mL) and DCM (20 mL) were added, and the mixture was stirred vigorously for 5 min. The organic layer was then washed with distilled water (2 × 20 mL) and the volatiles removed in vacuo. The residue was purified via column chromatography (SiO_2, DCM) and the volatiles removed in vacuo affording **DA-TMS** as a light brown solid (295 mg, 1.22 mmol, 67%). ^1H NMR (400 MHz, $CDCl_3$, 298 K) *δ*: 8.79 (d, *J* = 2.7 Hz, 1H, H_c), 8.14 (d, *J* = 9.0 Hz, 1H, H_d), 7.81 (m, 1H, H_a), 7.69 (d, *J* = 9.0 Hz, 1H, H_e), 6.54 (t, *J* = 1.6 Hz, 1H, H_b), 0.31 (s, 1H, Hf). ^{13}C NMR (100 MHz, $CDCl_3$, 298 K) *δ*: 152.7, 146.2, 143.6, 132.4, 127.9, 116.7, 109.3, 100.7, 100.3, 0.3. HR ESI-MS ($CHCl_3$/CH_3CN) *m/z* = 243.1063 $[M+H]^+$ (calcd for $C_{12}H_{14}N_4Si$, 243.1066).

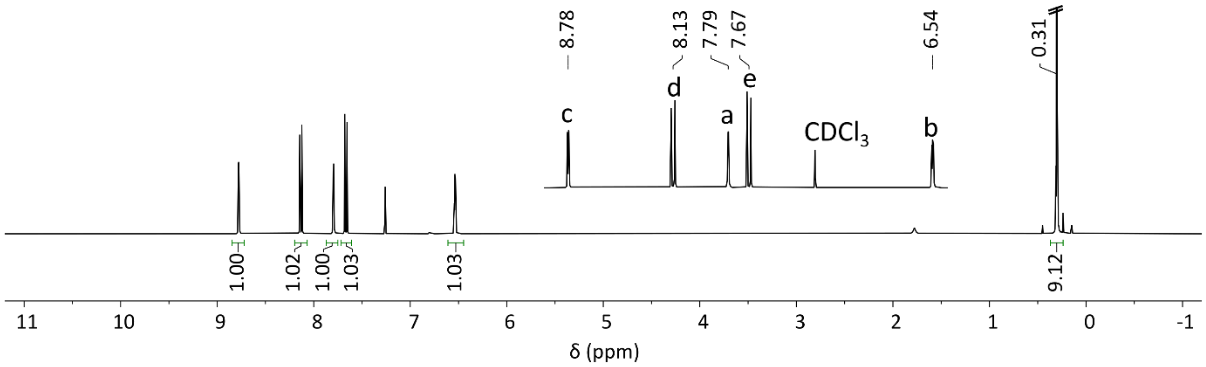

Figure S7. ^1H NMR ($CDCl_3$, 400 MHz, 298K) spectrum of **DA-TMS**.

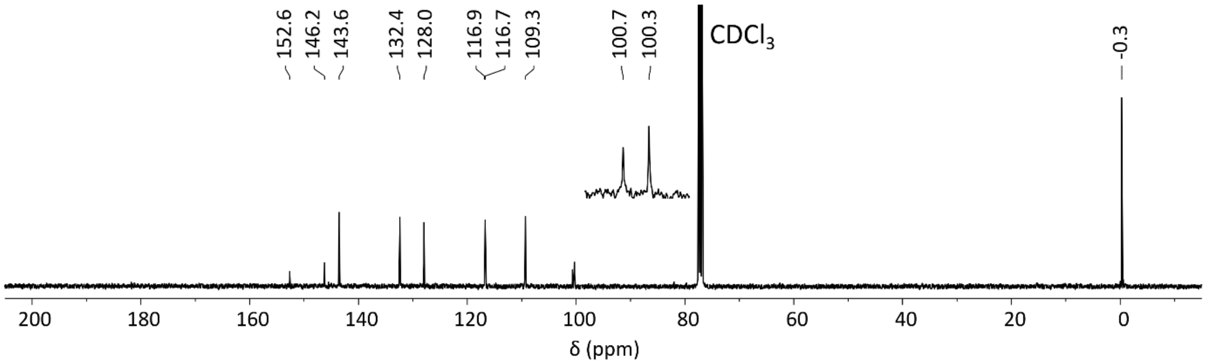

Figure S8. ^{13}C NMR ($CDCl_3$, 100 MHz, 298K) spectrum of **DA-TMS**.

1.2.5. DD-H

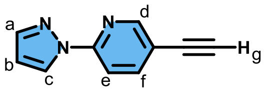

TMS-acetylene **DD-TMS** (50 mg, 0.21 mmol) was dissolved in DCM (30 mL) and MeOH (15 mL), K_2CO_3 (143 mg, 1.04 mmol) added and the suspension stirred at rt for 90 min. The mixture was diluted with water (100 mL) and DCM (50 mL), the organic phase separated, dried over $MgSO_4$, filtered, and the volatiles removed in vacuo to afford acetylene **DD-H** as an off-white solid (30 mg, 0.18 mmol, 86%). ^{1}H NMR (400 MHz, [D$_6$]DMSO): δ 8.62 (dd, J = 2.7, 0.7 Hz, 1H, H_c), 8.59 (dd, J = 2.2, 0.8 Hz, 1H, H_d), 8.08 (dd, J = 8.5, 2.2 Hz, 1H, H_f). 7.93 (dd, J = 8.5, 0.8 Hz, 1H, H_e), 7.87 (dd, J = 1.7, 0.7 Hz, 1H, H_a), 6.61 (dd, J = 2.7, 1.7 Hz, 1H, H_b), 4.48 (s, 1H, H_g). ^{13}C NMR (100 MHz, [D$_6$]DMSO): δ 151.1, 150.0, 142.9, 142.4, 127.4, 116.5, 111.7, 108.8, 84.3, 80.0. HR MS (ESI) m/z: [M+Na]$^+$ Calcd for $C_{10}H_7N_3Na$ 169.0640; found 169.0611.

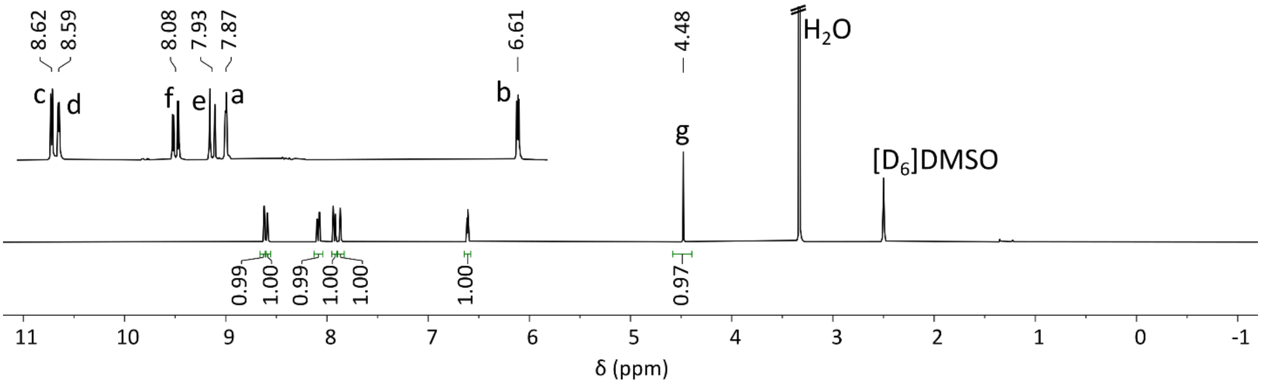

Figure S9. ^{1}H NMR ([D$_6$]DMSO, 400 MHz, 298K) spectrum of **DD-H**.

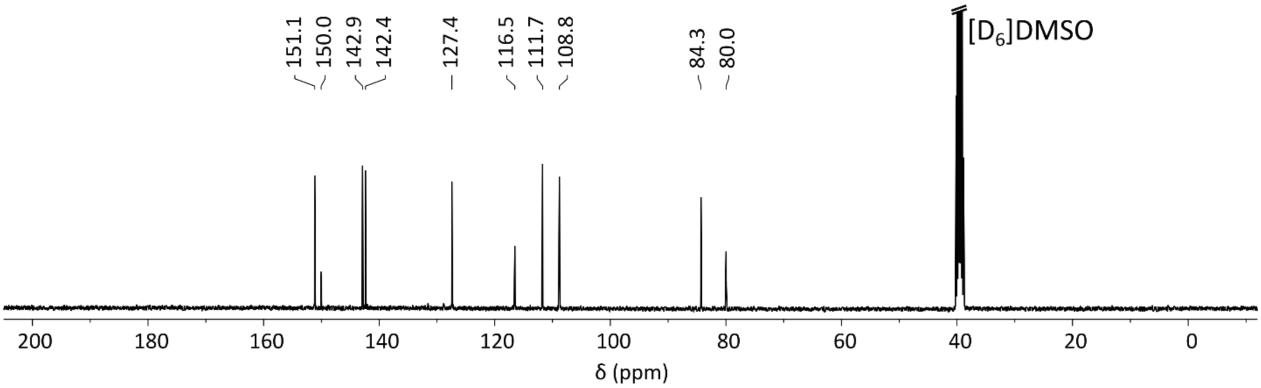

Figure S10. ^{13}C NMR ([D$_6$]DMSO, 100 MHz, 298K) spectrum of **DD-H**.

1.2.6. AD-H

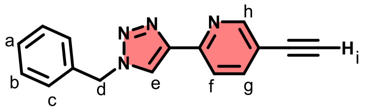

TMS-acetylene **AD-TMS** (50 mg, 0.15 mmol) was dissolved in DCM (30 mL) and MeOH (15 mL), K_2CO_3 (103 mg, 0.75 mmol) added and the suspension stirred at rt for 90 min. The mixture was diluted with water (100 mL) and DCM (50 mL), the organic phase separated, dried over $MgSO_4$, filtered, and the volatiles removed in vacuo to afford acetylene **AD-H** as an off-white solid (32 mg, 0.12 mmol, 82%). ^{1}H NMR (400 MHz, [D$_6$]DMSO): δ 8.74 (s, 1H, H$_e$), 8.68 (dd, *J* = 2.1, 1.0 Hz, 1H, H$_h$), 8.03 (dd, *J* = 8.2, 1.0 Hz, 1H, H$_f$), 7.99 (dd, *J* = 8.2, 2.1 Hz, 1H, H$_g$), 7.39–7.34 (m, 5H, H$_{a,b,c}$), 5.68 (s, 2H, H$_d$), 4.49 (s, 1H, H$_i$). ^{13}C NMR (100 MHz, [D$_6$]DMSO): δ 152.2, 149.3, 146.8, 140.1, 135.9, 128.8, 128.2, 128.0, 124.1, 119.0, 117.6, 84.5, 80.5, 53.1. HR MS (ESI) *m/z*: [M+Na]$^+$ Calcd for $C_{16}H_{12}N_4Na$ 283.0954; found 283.0974.

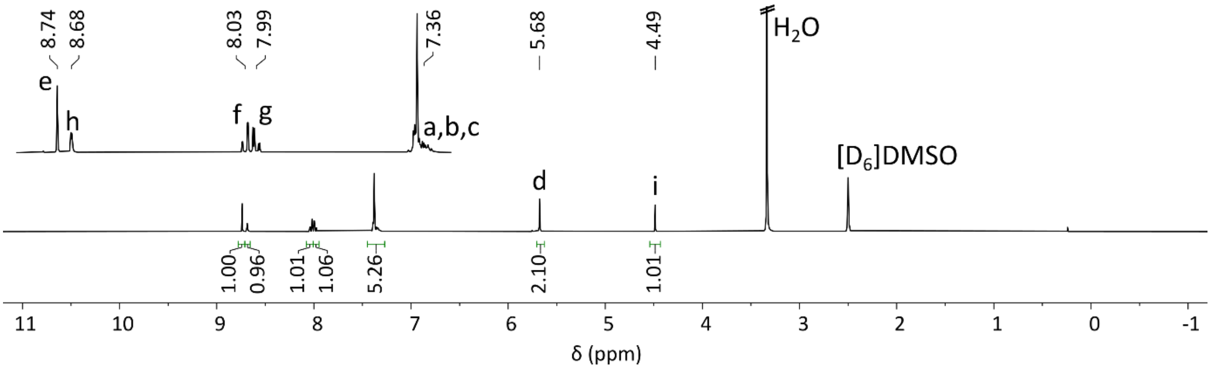

Figure S11. ^{1}H NMR ([D$_6$]DMSO, 400 MHz, 298K) spectrum of **AD-H**.

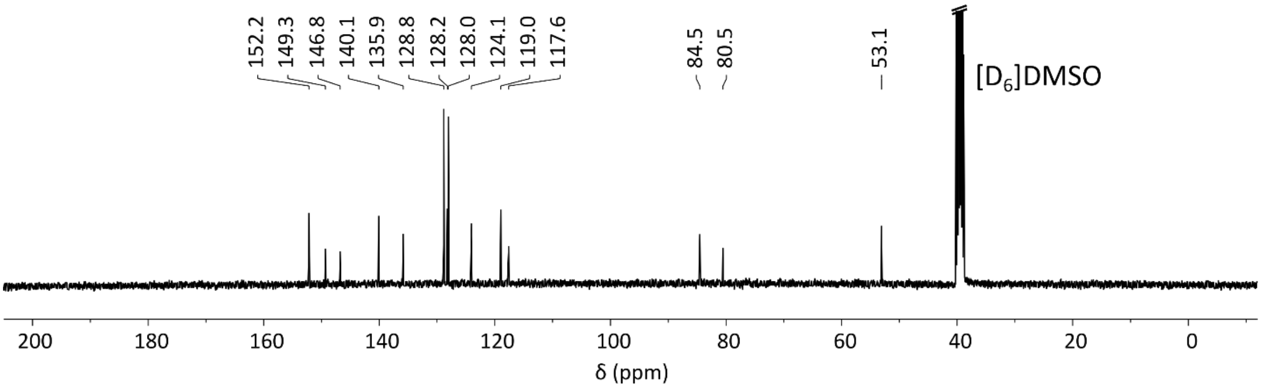

Figure S12. ^{13}C NMR ([D$_6$]DMSO, 100 MHz, 298K) spectrum of **AD-H**.

1.2.7. DA-H

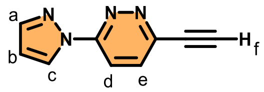

TMS-acetylene **DA-TMS** (400 mg, 1.65 mmol) was dissolved in DCM (30 mL) and MeOH (15 mL), K_2CO_3 (1.14 g, 8.25 mmol) added and the suspension stirred at rt for 90 min. The mixture was diluted with water (100 mL) and DCM (50 mL), the organic phase separated, dried over $MgSO_4$, filtered, and the volatiles removed in vacuo to afford acetylene **DA-H** as an off-white solid (240 mg, 1.41 mmol, 86%). ^{1}H NMR (400 MHz, [D$_6$]DMSO): δ 8.85 (dd, J = 2.7, 0.7 Hz, 1H, H_a), 8.21 (d, J = 9.1 Hz, 1H, H_d), 8.03 (d, J = 9.1 Hz, 1H, H_e), 7.98 (dd, J = 1.7, 0.7 Hz, 1H, H_c), 6.70 (dd, J = 2.7, 1.7 Hz, 1H, H_b), 4.79 (s, 1H, H_f). ^{13}C NMR (100 MHz, [D$_6$]DMSO): δ 152.6, 145.3, 143.8, 133.4, 128.0, 117.3, 109.7, 85.2, 79.9. HR MS (ESI) m/z: $[M+H]^+$ Calcd for $C_9H_7N_4$ 171.0665; found 171.0675.

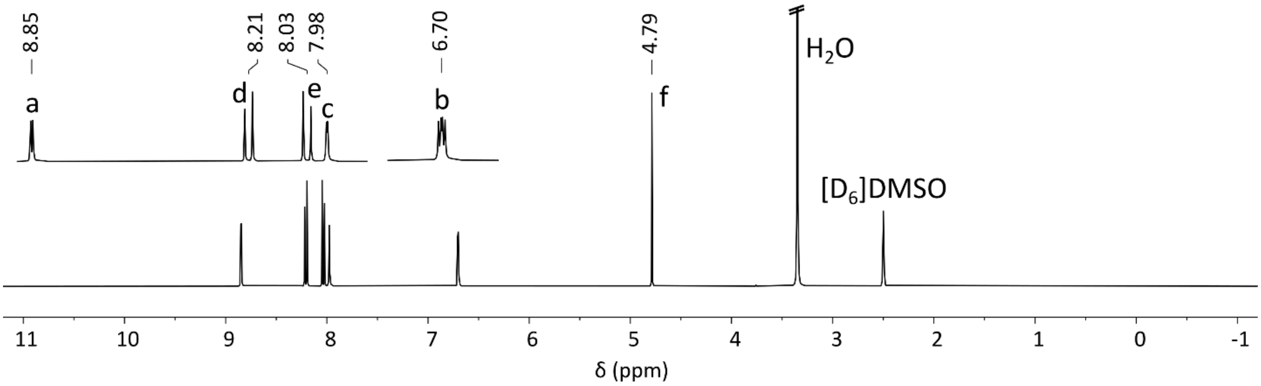

Figure S13. ^{1}H NMR ([D$_6$]DMSO, 400 MHz, 298K) spectrum of **DA-H**.

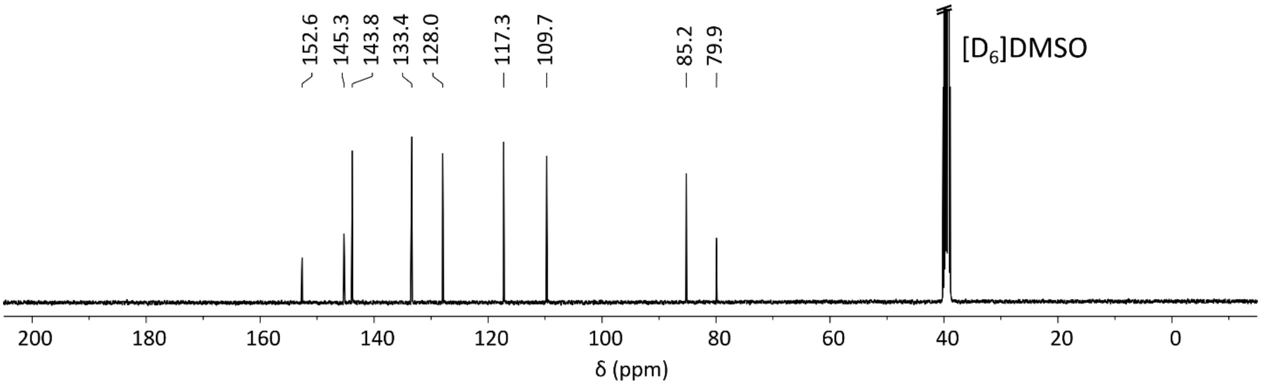

Figure S14. ^{13}C NMR ([D$_6$]DMSO, 100 MHz, 298K) spectrum of **DA-H**.

1.2.8. AA-H

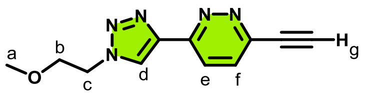

TMS-acetylene **AA-TMS** (50 mg, 0.22 mmol) was dissolved in DCM (30 mL) and MeOH (15 mL), K_2CO_3 (143 mg, 1.04 mmol) added and the suspension stirred at rt for 90 min. The mixture was diluted with water (100 mL) and DCM (50 mL), the organic phase separated, dried over $MgSO_4$, filtered, and the volatiles removed in vacuo to afford acetylene **DD-H** as an off-white solid (29 mg, 0.18 mmol, 84%). ^{1}H NMR (400 MHz, [D$_6$]DMSO): δ 8.89 (s,1H, H_d), 8.25 (d, J = 8.8 Hz, 1H, H_e), 7.95 (d, J = 8.8 Hz, 1H, H_f), 4.78 (s, 1H, H_g), 4.66 (t, J = 5.2 Hz, 2H, H_c), 3.82 (t, J = 5.2 Hz, 2H, H_b), 3.27 (s, 3H, H_a). ^{13}C NMR (100 MHz, [D$_6$]DMSO): δ 151.6, 145.6, 143.7, 131.1, 124.7, 122.8, 85.2, 80.5, 69.9, 58.0, 49.7. HR MS (ESI) *m/z*: [M+Na]$^+$ Calcd for $C_{11}H_{11}N_5NaO$ 252.0856; found 252.0873.

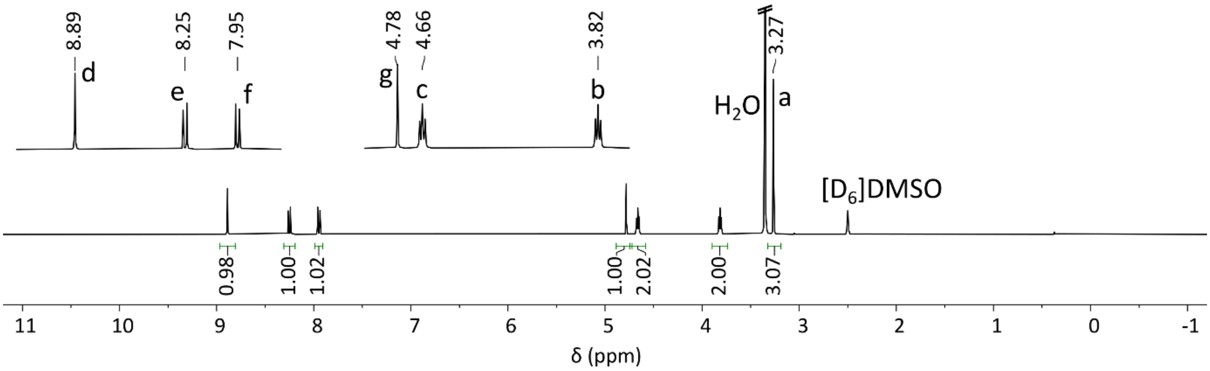

Figure S15. ^{1}H NMR ([D$_6$]DMSO, 400 MHz, 298K) spectrum of **AA-H**.

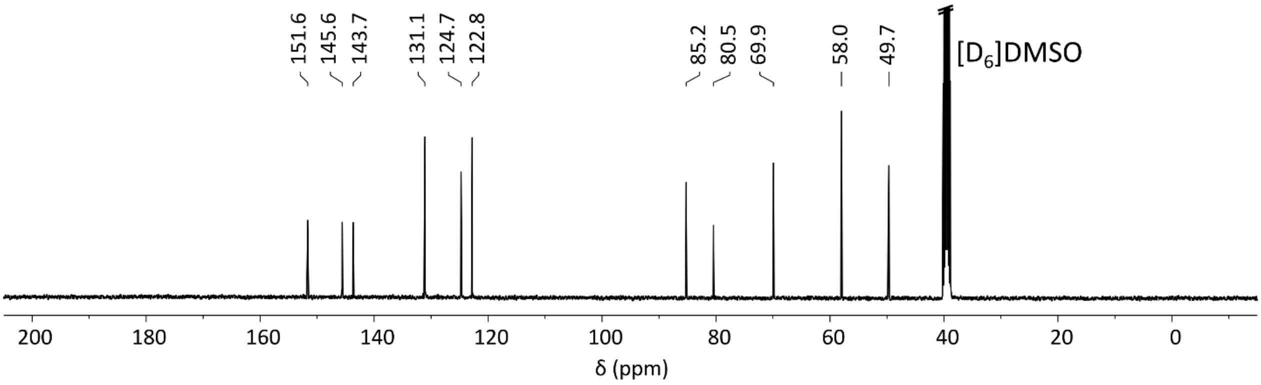

Figure S16. ^{13}C NMR ([D$_6$]DMSO, 100 MHz, 298K) spectrum of **AA-H**.

1.2.9. AAER

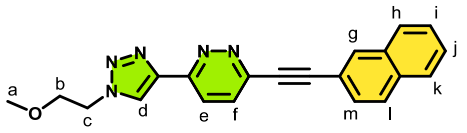

Compound **AA-TMS** (121 mg, 0.40 mmol) and sodium carbonate (87 mg, 0.82 mmol) were stirred in methanol (10 mL) at 50 °C for 1 h. The mixture was filtered, and the filtrate was dried in vacuo. The residue was added to a solution of 1:1 triethylamine/dioxane (20 mL) and deoxygenated. 2-Bromonaphthalene (195 mg, 2.38 mmol), tris(dibenzylideneacetone)dipalladium (19 mg, 0.021 mmol), tri-*tert*-butylphosphonium tetrafluoroborate (27 mg, 0.093 mmol) and copper iodide (9 mg, 0.05 mmol) were added to the deoxygenated mixture. The mixture was then stirred at 80 °C for 17 hours under a nitrogen atmosphere. 0.1 M EDTA/NH_4OH (aq.; 20 mL) and DCM (20 mL) were added, and the mixture was stirred vigorously for 5 min. The organic layer was then washed with brine (2 × 20 mL) and the volatiles removed in vacuo. The residue was purified via column chromatography (SiO_2, DCM) and the volatiles removed in vacuo affording **AAER** as a white solid (46 mg, 0.13 mmol, 32%). ^1H NMR (400 MHz, $CDCl_3$, 298 K) δ: 8.57 (s, 1H, H_d), 8.33 (d, J = 8.8 Hz, 1H, H_e), 8.20 (d, J = 1.6 1H, H_g), 7.89–7.82 (m, 3H, $H_{h,k,l}$), 7.76 (d, J = 8.7 Hz, 3H, H_f), 7.67 (dd, J = 8.5, 1.6 Hz, 1H, H_m), 7.56–7.51 (m, 2H, $H_{i,j}$), 4.65 (t, J = 5.0 Hz, 2H, H_c), 3.83 (t, J = 5.1 Hz, 2H, H_b), 3.40 (s, 3H, H_a). ^{13}C NMR (100 MHz, $CDCl_3$, 298 K) δ: 151.4, 147.0, 145.1, 133.4, 132.8, 132.7,130.2, 128.3, 128.2, 128.1, 127.9, 127.4, 126.8, 124.2, 122.6, 118.9, 94.5, 86.4, 70.5, 59.1, 50.7. HR ESI-MS ($CHCl_3$/MeOH) m/z = 356.1500 $[M+H]^+$ (calcd for $C_{21}H_{17}N_5O$, 356.1506).

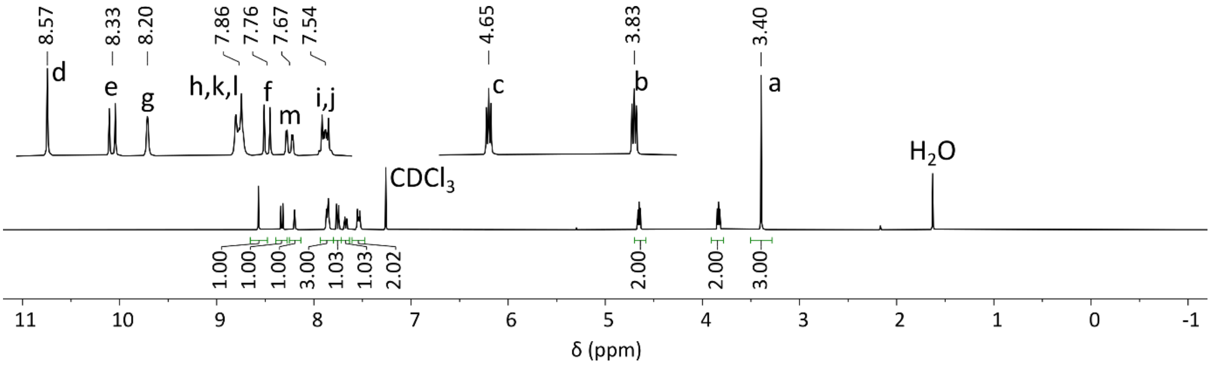

Figure S17. ^1H NMR ($CDCl_3$, 400 MHz, 298K) spectrum of **AAER**.

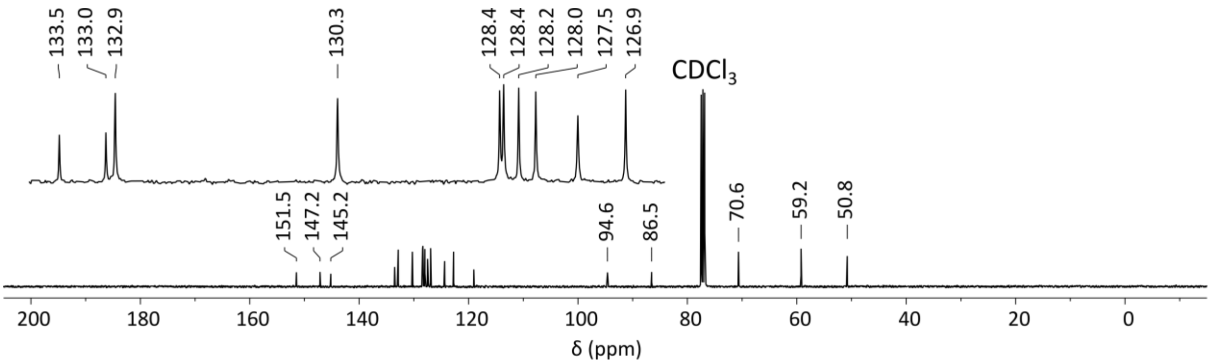

Figure S18. ^{13}C NMR ($CDCl_3$, 100 MHz, 298K) spectrum of **AAER**.

1.2.10. AAEP

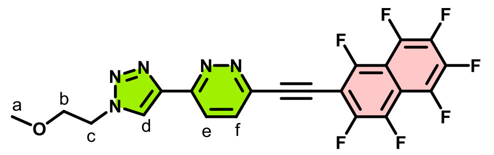

Compound **AA-TMS** (120 mg, 0.40 mmol) and sodium carbonate (60 mg, 0.57 mmol) were stirred in methanol (10 mL) at 50 °C for 1 h. The mixture was filtered, and the filtrate was dried in vacuo. The residue was added to a solution of 1:1 triethylamine/dioxane (35 mL) and deoxygenated. 2-Bromo-1,3,4,5,6,7,8-heptafluoronaphthalene (111 mg, 0.333 mmol), tris(dibenzylideneacetone)-dipalladium (21 mg, 0.023 mmol), tri-*tert*-butylphosphonium tetrafluoroborate (24 mg, 0.083 mmol), and copper iodide (9 mg, 0.05 mmol) were added to the deoxygenated mixture. The mixture was then stirred at 80 °C for 17 h under a nitrogen atmosphere. 0.1 M EDTA/NH_4OH (aq.; 20 mL) and DCM (20 mL) were added, and the mixture was stirred vigorously for 5 min. The organic layer was then washed with distilled water (2 × 20 mL) and the volatiles removed in vacuo. The residue was purified via column chromatography (SiO_2, 10% acetone/DCM) and the volatiles removed in vacuo affording **AAEP** as a brown solid (16 mg, 0.033 mmol, 8%). ^1H NMR (400 MHz, $CDCl_3$, 298 K) δ: 8.59 (s, 1H, H_d), 8.38 (d, J = 8.7 Hz, 1H, H_e), 7.82 (d, J = 8.7 Hz, 1H, H_f), 4.66 (t, J = 4.8 Hz, 2H, H_c), 3.84 (t, J = 4.8 Hz, 2H, H_b), 3.40 (s, 3H, H_a). ^{13}C NMR (100 MHz, $CDCl_3$, 298 K) δ: 152.2, 145.8, 144.9, 130.7, 124.7, 122.7, 111.8, 98.3, 78.8, 70.6, 59.2, 50.8. The naphthalene ^{13}C signals are poorly resolved due to ^{13}C–^{19}F coupling. ^{19}F NMR (400 MHz, $CDCl_3$, 298 K) δ: –110.4 (dd, J = 67.9, 18.6 Hz), –132.2 (m), –142.3 (dtt, J = 62.7, 16.6, 4.4 Hz), –144.9 (dt, J = 58.7, 16.0 Hz), –147.9 (dtt, J = 58.7, 17.8, 4.4 Hz), –150.4 (tt, J = 18.6, 4.4 Hz), –154.1 (t, J = 20.0 Hz). HR ESI-MS ($CHCl_3$/MeOH) m/z = 482.0840 $[M+H]^+$ (calcd for $C_{21}H_{11}F_7N_5O$, 482.0846).

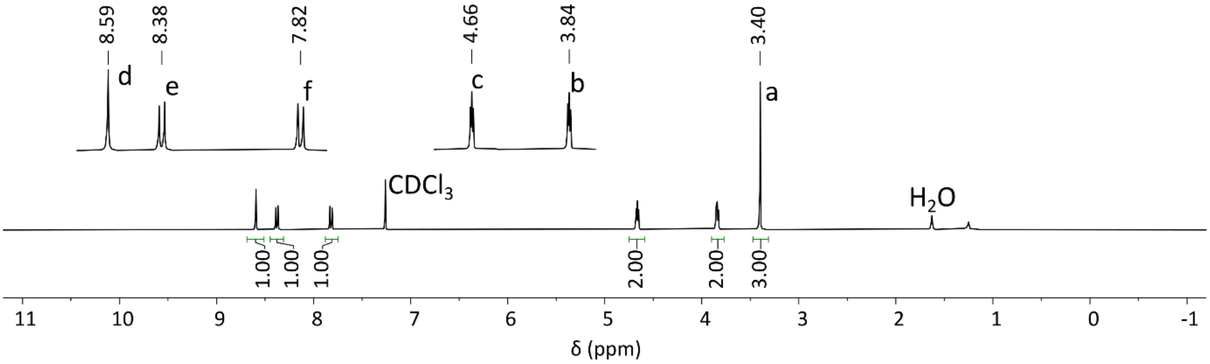

Figure S19. ^1H NMR ($CDCl_3$, 400 MHz, 298K) spectrum of **AAEP**.

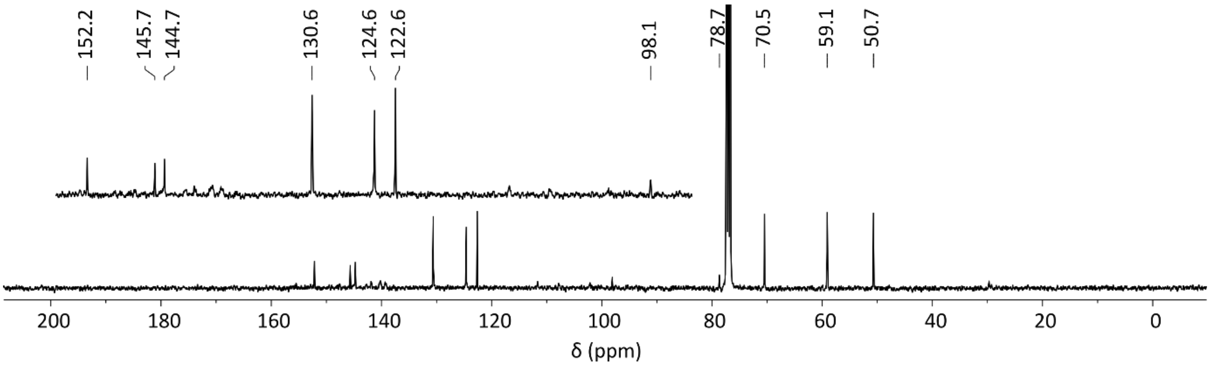

Figure S20. ^{13}C NMR ($CDCl_3$, 100 MHz, 298K) spectrum of **AAEP**.

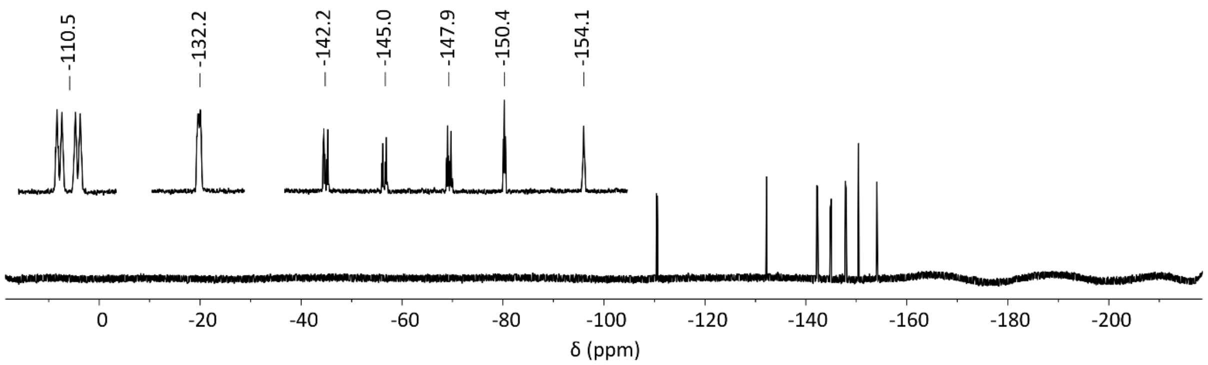

Figure S21. ^{19}F NMR ($CDCl_3$, 376 MHz, 298K) spectrum of **AAEP**.

1.2.11. ADER

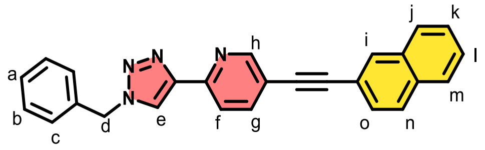

Compound **AD-TMS** (108 mg, 0.33 mmol) and sodium carbonate (80 mg, 0.76 mmol) were stirred in methanol (10 mL) at 50 °C for one hour. The mixture was filtered, and the filtrate was dried in vacuo. The residue was added to a solution of 1:1 triethylamine/1,4-dioxane (20 mL) and deoxygenated. 2-Bromonaphthalene (125 mg, 0.602 mmol), tris(dibenzylideneacetone)dipalladium (14 mg, 0.015 mmol), tri-*tert*-butylphosphonium tetrafluoroborate (17 mg, 0.060 mmol), and copper iodide (6 mg, 0.03 mmol) were added to the deoxygenated mixture. The mixture was then stirred at 85 °C for 17 hours under a nitrogen atmosphere. 0.1 M EDTA/NH_4OH (aq.; 20 mL) and DCM (20 mL) were added, and the mixture was stirred vigorously for 5 min. The organic layer was then washed with brine (2 × 20 mL) and the volatiles removed in vacuo. The residue was purified via column chromatography (SiO_2, 10% acetone/DCM) and the volatiles removed in vacuo affording **ADER** as a pale yellow solid (64 mg, 0.17 mmol, 51%). ^1H NMR (400 MHz, $CDCl_3$, 298 K) δ: 8.72 (s, 1H, H_h), 8.19 (d, J = 8.3 Hz, 1H, H_f), 8.10–8.06 (m, 2H, $H_{e,i}$), 7.93 (dd, J = 8.2, 1.8 Hz, 1H, H_g), 7.86–7.80 (m, 3H, $H_{j,m,n}$), 7.59 (dd, J = 8.5, 1.6 Hz, 1H, H_o), 7.54–7.49 (m, 2H, $H_{k,l}$), 7.43–7.32 (m, 5H, $H_{a,b,c}$), 5.59 (s, 2H, H_d). ^{13}C NMR (100 MHz, $CDCl_3$, 298 K) δ: 151.9, 149.0, 148.3, 139.5, 134.4, 133.12, 133.05, 131.89, 122.5, 119.9, 119.7, 119.6, 129.3, 129.0, 128.4, 128.30, 128.28, 128.0, 127.9, 127.1, 126.8, 93.7, 86.7, 54.6. HR ESI-MS ($CHCl_3$/MeOH) m/z = 387.1599 [M+H]$^+$ (calcd for $C_{26}H_{18}N_4$, 387.1610).

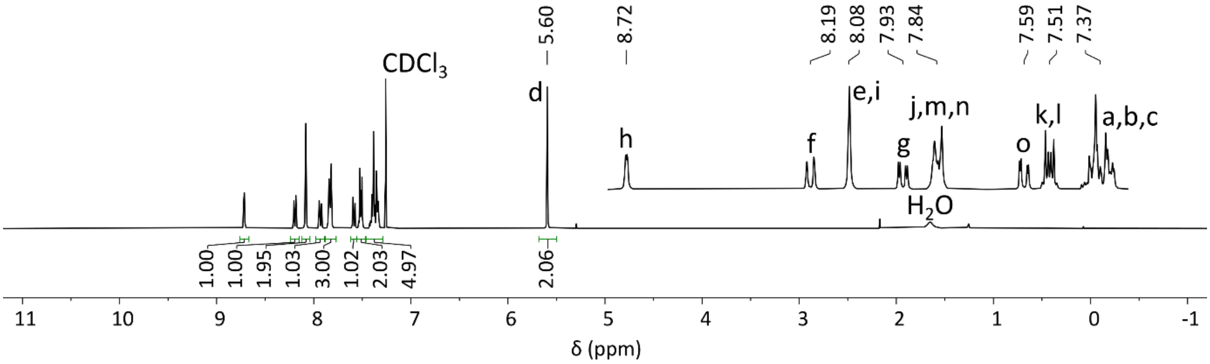

Figure S22. ^1H NMR ($CDCl_3$, 400 MHz, 298K) spectrum of **ADER**.

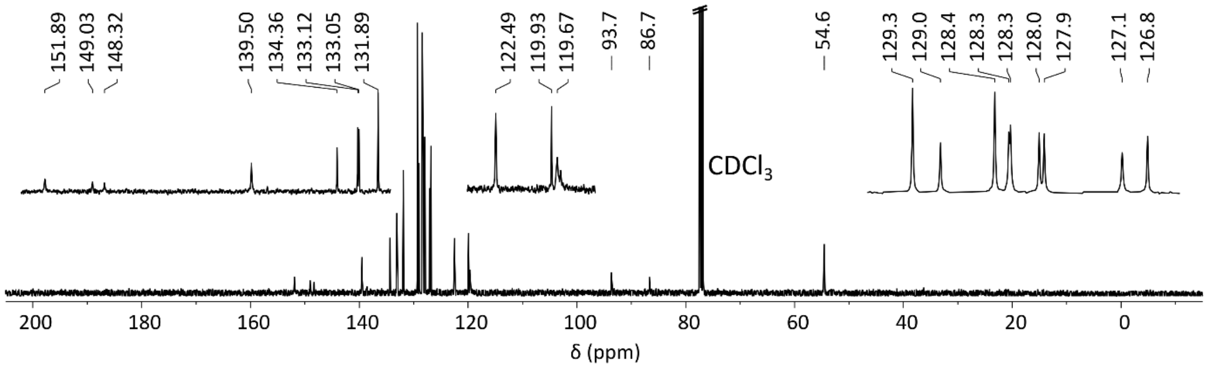

Figure S23. ^{13}C NMR ($CDCl_3$, 100 MHz, 298K) spectrum of **ADER**.

1.2.12. ADEP

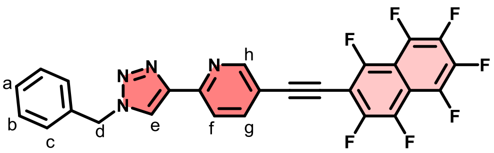

Compound **AD-TMS** (109 mg, 0.33 mmol) and sodium carbonate (100 mg, 0.943 mmol) were stirred in methanol (10 mL) at 50 °C for one hour. The mixture was filtered, and the filtrate was dried in vacuo. The residue was added to a solution of 1:1 triethylamine/1,4-dioxane (20 mL) and deoxygenated. 2-Bromo-1,3,4,5,6,7,8-heptafluoronaphthalene (100 mg, 0.301 mmol), tris(dibenzylideneacetone)-dipalladium (19 mg, 0.021 mmol), triphenylphosphine (23 mg, 0.088 mmol), and copper iodide (12 mg, 0.063 mmol) were added to the deoxygenated mixture. The mixture was then stirred at 80 °C for 17 hours under a nitrogen atmosphere. 0.1 M EDTA/NH_4OH (aq.; 20 mL) and DCM (20 mL) were added, and the mixture was stirred vigorously for 5 min. The organic layer was then washed with distilled water (2 × 20 mL) and the volatiles removed in vacuo. The residue was purified via column chromatography (SiO_2, 10% acetone/DCM). The product was then separated from impurities by suspending in $CHCl_3$ and centrifuged, affording **ADEP** as a sparingly soluble, off-white solid (82 mg, 0.16 mmol, 49%). ^1H NMR (400 MHz, $CDCl_3$, 298 K) δ: 8.77 (d, J = 2.2 Hz, 1H, H_h), 8.26 (d, J = 8.2 Hz, 1H, H_f), 8.12 (s, 1H, H_e), 8.01 (dd, J = 8.3, 2.2 Hz, 1H, H_g), 7.40 (m, 5H, $H_{a,b,c}$), 5.63 (s, 1H, H_d). Sufficiently intense ^{13}C NMR data could not be obtained due the poor solubility of the compound. ^{19}F NMR (400 MHz, $CDCl_3$, 298 K) δ: -112.2 (dd, J = 66.1, 17.3 Hz), -132.8 (m), -142.7 (dtt, J = 66.1, 17.3, 4.5 Hz), -145.2 (dt, J = 58.2, 15.7 Hz), -148.2 (dtt, J = 58.2, 17.3, 4.5 Hz), -151.2 (tt, J = 19.8, 4.5 Hz), -154.4 (t, J = 24.5 Hz). HR ESI-MS ($CHCl_3$/MeOH) m/z = 513.0949 $[M+H]^+$ (calcd for $C_{26}H_{12}F_7N_4$, 513.0945).

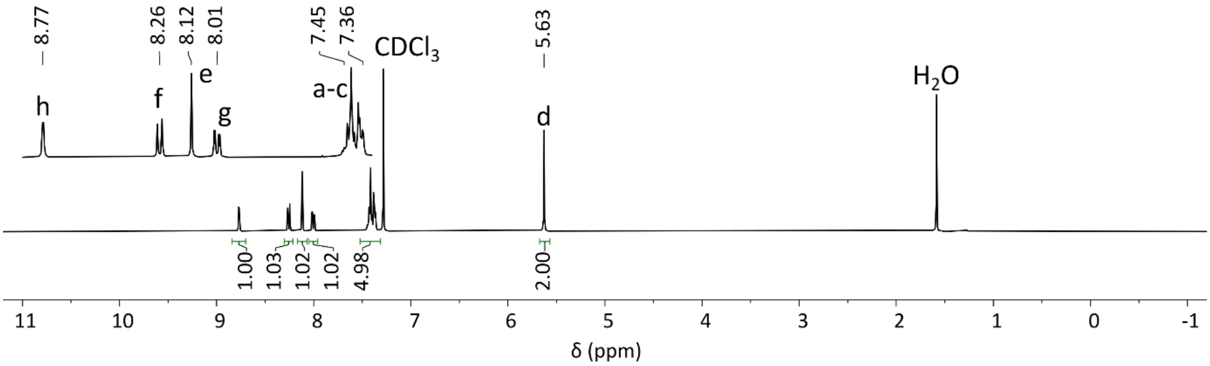

Figure S24. ^{1}H NMR ($CDCl_3$, 400 MHz, 298K) spectrum of **ADEP.**

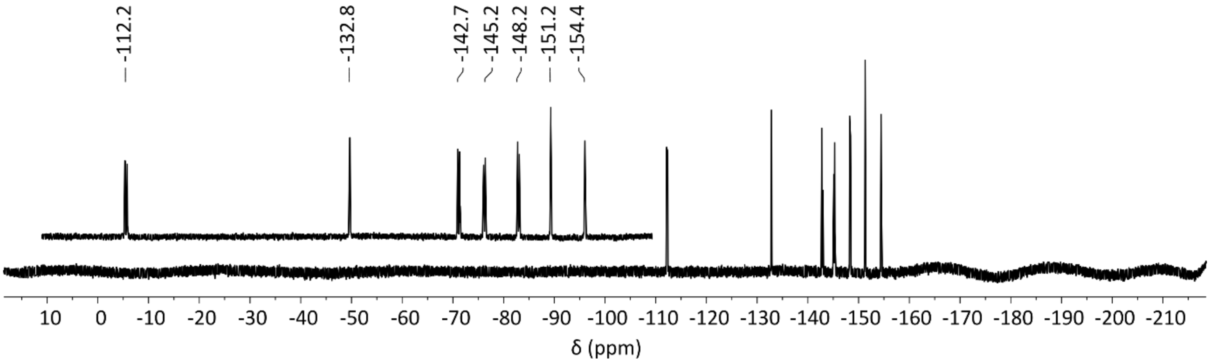

Figure S25. ^{19}F NMR ($CDCl_3$, 376 MHz, 298K) spectrum of **ADEP.**

1.2.13. DDER

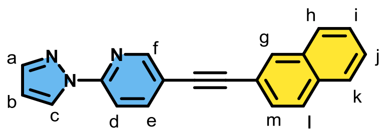

Compound **DD-TMS** (115 mg, 0.48 mmol) and sodium carbonate (90 mg, 0.85 mmol) were stirred in methanol (10 mL) at 50 °C for one hour. The mixture was filtered, and the filtrate was dried in vacuo. The residue was added to a solution of 1:1 triethylamine/Dioxane (53 mL) and deoxygenated. 2-Bromonaphthalene (142 mg, 0.686 mmol), tris(dibenzylideneacetone)dipalladium (22 mg, 0.024 mmol), tri-*tert*-butylphosphonium tetrafluoroborate (19 mg, 0.065 mmol), and copper iodide (10 mg, 0.053 mmol) were added to the deoxygenated mixture. The mixture was then stirred at 80 °C for 17 hours under a nitrogen atmosphere. 0.1 M EDTA/NH_4OH (aq.; 20 mL) and DCM (20 mL) were added, and the mixture was stirred vigorously for 5 min. The organic layer was then washed with brine (2 × 20 mL) and the volatiles removed in vacuo. The residue was purified via column chromatography (SiO_2, DCM), affording **DDER** as an off-white solid (23 mg, 0.077 mmol, 16%). ^{1}H NMR (400 MHz, $CDCl_3$, 298 K) δ: 8.60 (dd, J = 2.2, 1.0 Hz, 1H, H_m), 8.59 (dd, J = 2.7, 0.8 Hz, 1H, H_c), 8.09 (d, J = 1.6 Hz, 1H, H_g), 8.01 (dd, J = 8.5, 0.9 Hz, 1H, H_d), 7.97 (dd, J = 8.5, 2.1 Hz, 1H, H_e), 7.87–7.81 (m, 3H, $H_{h,k,l}$), 7.77 (dd, J = 1.6, 0.8 Hz, 1H, H_a), 7.59 (dd, J = 8.5, 1.7 Hz, 1H, H_f), 7.54–7.49 (m, 2H, $H_{i,j}$), 6.50 (dd, J = 2.7, 1.6 Hz, 1H, H_b). ^{13}C NMR (100 MHz, $CDCl_3$, 298 K) δ: 150.9, 150.4, 142.6, 141.3, 133.15, 133.11 131.9, 129.1, 128.3, 128.0, 128.0, 127.4, 127.1, 126.9, 120.0, 118.1, 112.0, 108.3, 93.2, 86.2. HR ESI-MS ($CHCl_3$/MeOH) *m/z* = 318.0996 $[M+H]^+$ (c alc. for $C_{20}H_{13}N_3Na$, 318.1002).

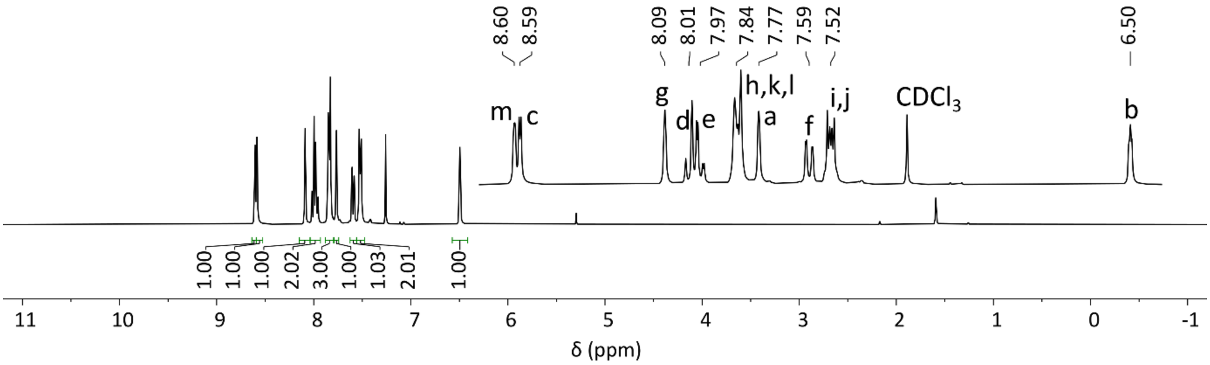

Figure S26. ^{1}H NMR ($CDCl_3$, 400 MHz, 298K) spectrum of **DDER**.

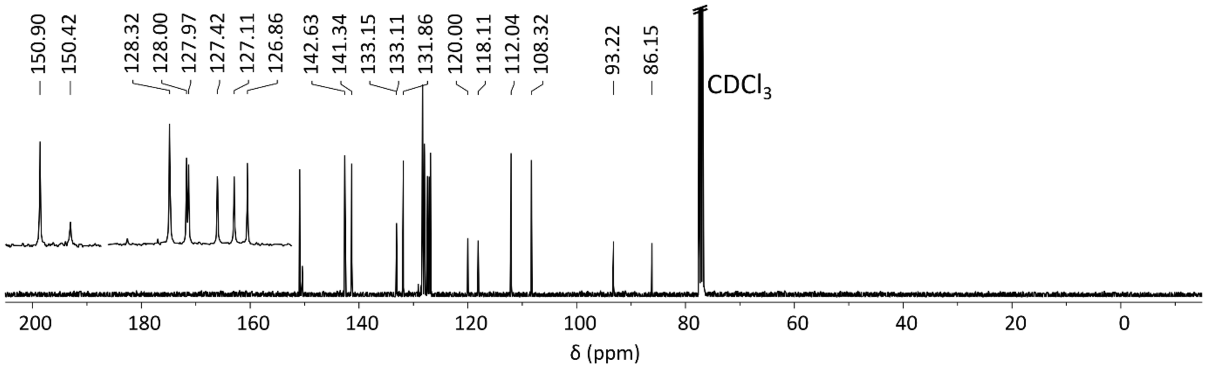

Figure S27. ^{13}C NMR ($CDCl_3$, 100 MHz, 298K) spectrum of **DDER**.

1.2.14. DDEP

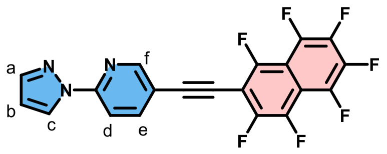

Compound **DD-TMS** (110 mg, 0.46 mmol) and sodium carbonate (100 mg, 0.94 mmol) were stirred in methanol (10 mL) at 50 °C for one hour. The mixture was filtered, and the filtrate was dried in vacuo. The residue was added to a solution of 1:1 triethylamine/1,4-dioxane (20 mL) and deoxygenated. 2-Bromo-1,3,4,5,6,7,8-heptafluoronaphthalene (182 mg, 0.55 mmol), tris(dibenzylideneacetone)-dipalladium (19 mg, 0.021 mmol), tri-*tert*-butylphosphonium tetrafluoroborate (21 mg, 0.072 mmol), and copper iodide (11 mg, 0.058 mmol) were added to the deoxygenated mixture. The mixture was then stirred at 85 °C for 53 hours under a nitrogen atmosphere. 0.1 M EDTA/NH_4OH (aq.; 20 mL) and DCM (20 mL) were added, and the mixture was stirred vigorously for 5 min. The organic layer was then washed with brine (2 × 20 mL) and the volatiles removed in vacuo. The residue was purified via column chromatography (SiO_2, DCM) and the volatiles removed in vacuo affording **DDEP** as a white solid (76 mg, 0.18 mmol, 40%). ^{1}H NMR (400 MHz, $CDCl_3$, 298 K) δ: 8.65 (d, J = 1.9 Hz, 1H, H_d), 8.60 (d, J = 2.7 Hz, 1H, H_c), 8.05–8.02 (m, 2H, $H_{e,f}$), 7.78 (d, J = 1.7 Hz, 1H, H_a), 6.51 (s, 1H, H_b). ^{13}C NMR data could not be obtained due the poor solubility of the compound. ^{19}F NMR (400 MHz, $CDCl_3$, 298 K) δ: -112.3 (dd, J = 67.0, 18.3 Hz), -132.8–-133.0 (m), -142.8 (dtt, J = 66.9, 16.7, 4.8 Hz), -145.2 (dt, J = 58.9, 16.7 Hz), -148.3 (dtt, J = 58.6, 17.7, 4.8 Hz), -151.3 (tt, J = 19.0, 4.2 Hz), -154.3–-154.5 (m). HR ESI-MS ($CHCl_3$/MeOH) m/z = 422.0516 $[M+H]^+$ (calcd for $C_{20}H_7F_7N_3$, 422.0523).

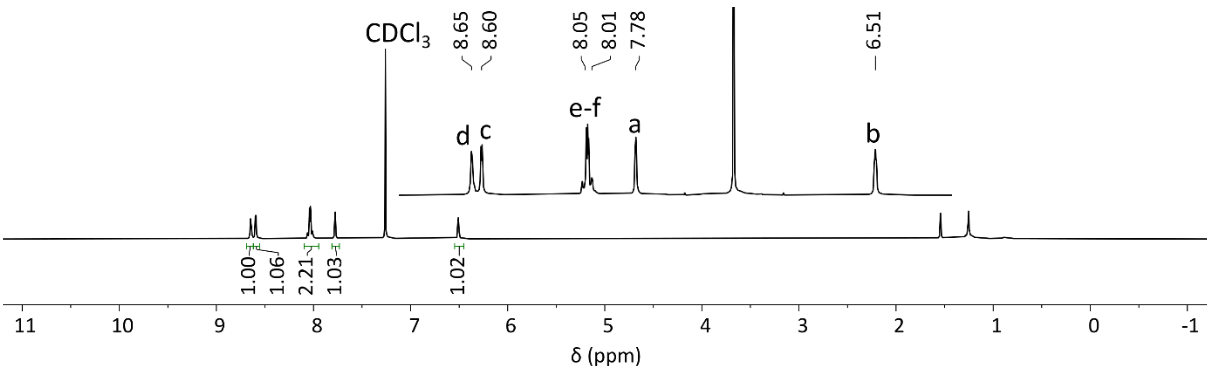

Figure S28. ^{1}H NMR ($CDCl_3$, 400 MHz, 298K) spectrum of **DDEP**.

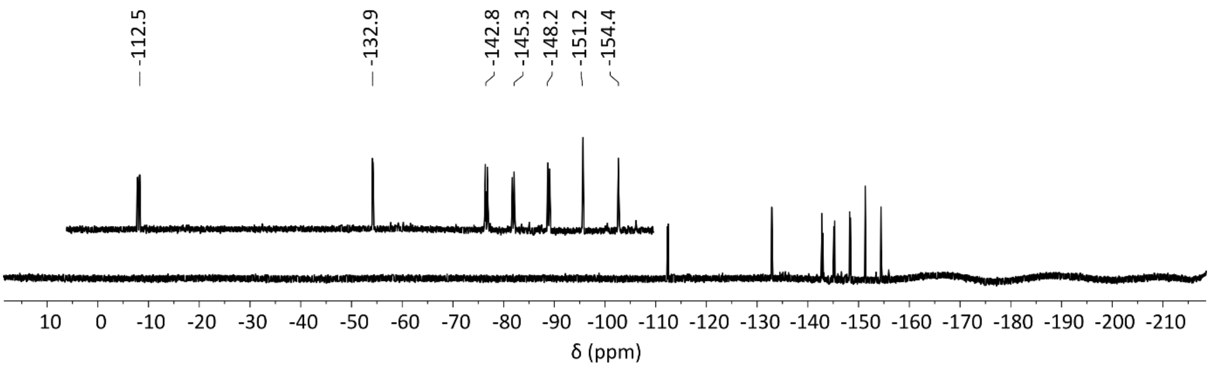

Figure S29. ^{19}F NMR ($CDCl_3$, 376 MHz, 298K) spectrum of **DDER.**

1.2.15. DAER

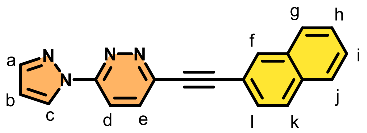

Compound **DA-TMS** (125 mg, 0.516 mmol) and sodium carbonate (140 mg, 1.32 mmol) were stirred in methanol (10 mL) at 50 °C for one hour. The mixture was filtered, and the filtrate was dried in vacuo. The residue was added to a solution of 1:1 triethylamine/THF (7 mL) and deoxygenated in a heavy-walled pressure tube. 2-Bromonaphthalene (500 mg, 2.41 mmol), tris(dibenzylideneacetone)dipalladium (34 mg, 0.037 mmol), tri-*tert*-butylphosphonium tetrafluoroborate (46 mg, 0.16 mmol), and copper iodide (31 mg, 0.16 mmol) were added to the deoxygenated mixture. The mixture was then stirred at 80 °C for 17 hours under a nitrogen atmosphere. 0.1 M EDTA/NH_4OH (aq.; 20 mL) and DCM (20 mL) were added, and the mixture was stirred vigorously for 5 min. The organic layer was then washed with distilled water (2 × 20 mL) and the volatiles removed in vacuo. The residue was purified via column chromatography (SiO_2, DCM) and the volatiles removed in vacuo affording **DAER** as an off-white solid (21 mg, 0.071 mmol, 14%). ^{1}H NMR (400 MHz, $CDCl_3$, 298 K) δ: 8.83 (d, J = 2.1 Hz, 1H, H_c), 8.20 (m, 2H, $H_{d,f}$), 7.86 (m, 3H, $H_{g,j,l}$), 7.83 (d, J = 1.6 Hz, 1H, H_a), 7.79 (d, J = 9.1 Hz, 1H, H_e), 7.66 (dd, J = 8.5, 1.7 Hz, 1H, H_g), 7.54 (m, 2H, $H_{h,i}$), 6.57 (dd, J = 2.7, 1.7 Hz, 1H, H_b). ^{13}C NMR (100 MHz, $CDCl_3$, 298 K) δ: 152.6, 146.7, 143.5, 133.5, 133.0, 132.9, 132.2, 128.4, 128.3, 128.2, 128.0, 127.9, 127.5, 127.0, 118.9, 116.9, 109.3, 94.6, 85.9. HR ESI-MS ($CHCl_3$/MeOH) m/z = 297.1135 $[M+H]^+$ (calcd for $C_{19}H_{13}N_4$, 297.1135).

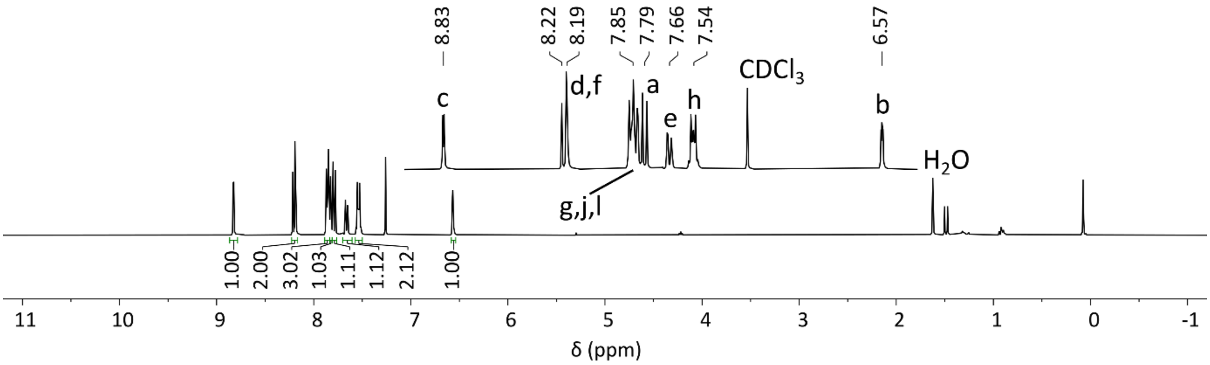

Figure S30. ^{1}H NMR ($CDCl_3$, 400 MHz, 298K) spectrum of **DAER**.

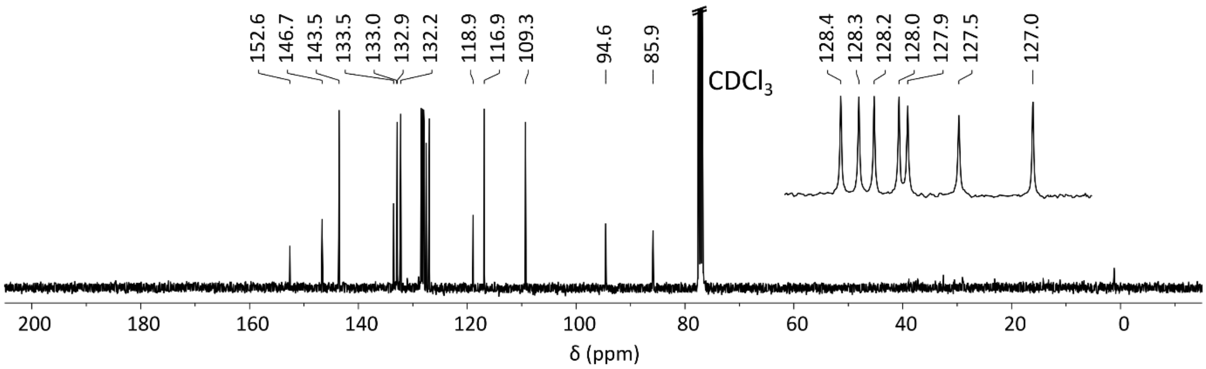

Figure S31. ^{13}C NMR ($CDCl_3$, 100 MHz, 298K) spectrum of **DAER**.

2. Complexations

2.1. General

All complexes were prepared in 5 mm NMR tubes from stock solutions of ligands and $[Pd(MeCN)_4](BF_4)_2$ to a volume of 600 µL, and reached equilibrium in the time required to collect the ^{1}H NMR spectrum. DOSY NMR spectra were collected at 1.88 mM Pd(II) to control for concentration effects. ^{1}H NMR integrations are reported per ligand. Impurities in the Pd(II) source appear at 7.28 and 6.68 ppm in [D$_6$]DMSO.

2.2. Equilibria

To calculate equilibrium constants (*K*) and *ΔG*, mol-fractions of the species were determined by ^{1}H NMR integration. The concentration of residual Pd(II) is assumed based on the stoichiometry. Due to the detection limit of ^{1}H NMR experiments, when a species is not detected its proportion is assumed to be 5% in the equilibrium constant calculations.

Equilibria for homoleptic complex formation after the addition of 0.5 eq. Pd(II) (K_1) are given in Equation 1.

$$\mathbf{[Pd(L)(X)_2]^{2+} + L \rightleftharpoons [Pd(L)_2]^{2+}} \qquad \textbf{(E1)}$$

$$K_1 = \frac{[\mathbf{Pd(L)_2}]}{[\mathbf{Pd(L)(X)_2}][\mathbf{L}]}$$

Table S1. Summary of equilibria for homoleptic species after addition of 0.5 eq. Pd(II).

	mol fraction				
**Ligand**	**M_1L_1**	**M_1L_2**	**K_1**	**ΔG (kJ mol^{-1})**	**M_1L_2 isomer**
**DA-TMS**	ND	>0.95	>67000	< –28	HT
**AD-TMS**	ND	>0.95	>67000	< –28	HT
**AA-TMS**	0.29	0.71	4600	–21	HT
**AAER**	0.15	0.85	21500	–25	HT
**AAEP**	0.33	0.67	3200	–20	HT
**ADER**	ND	>0.95	>200000	< –30	HT
**ADEP**	ND	>0.95	>200000	< –30	HT
**DAER**	ND	>0.95	>200000	< –30	HT

Mol fractions determined by ^{1}H NMR integration. ND = not detected.

Equilibria for homoleptic complex formation after the addition of 1.0 eq. Pd(II) (K_2) are given in Equation 2.

$$[Pd(L)_2]^{2+} + Pd \rightleftharpoons 2[Pd(L)(X)_2]^{2+} \quad \textbf{(E2)}$$

$$K_2 = \frac{[\mathbf{Pd(L)}]^2}{[\mathbf{Pd(L)(X)_2}][(\mathbf{Pd})]}$$

Table S2. Summary of equilibria for homoleptic species after addition of 1.0 eq. Pd(II).

	**mol fraction**				
**Ligand**	**M_1L_1**	**M_1L_2**	***K_2***	***ΔG* (kJ mol^{-1})**	**M_1L_2 isomer**
**AA-TMS**	0.77	0.23	11.2	–6.0	HT
**DD-TMS**	>0.95	ND	>360	< –14.6	-
**AAER**	0.82	0.18	21	–7.6	HT
**AAEP**	0.77	0.23	11.1	–6.0	HT
**ADER**	0.53	0.47	1.3	–0.6	HT
**ADEP**	0.5	0.5	1.0	0	HT
**DAER**	0.51	0.49	0.9	0.2	HT
**DDER**	>0.95	ND	>360	< –14.5	-
**DDEP**	>0.95	ND	>360	< –14.5	-

Mol fractions determined by ^{1}H NMR integration. ND = not detected.

Equilibria for heteroleptic complex formation (K_{het}) are given in Equation 3.

$$\mathbf{homo} + \mathbf{homo'} \rightleftharpoons 2\mathbf{het} \quad \textbf{(E3)}$$

$$K_{het} = \frac{[\mathbf{het}]^2}{[\mathbf{homo}][(\mathbf{homo'})]}$$

Table S3. Summary of equilibria for heteroleptic species after addition of 0.5 eq. Pd(II).

	**mol fraction**				
**Ligands**	**Homo**	**Hetero**	***K_{het}***	***ΔG* (kJ mol^{-1})**	**M_1L_2 isomer**
**AA-TMS:DD-TMS**	ND	>0.95	>360	< –14.5	4:1 HT:HH
**AD-TMS:DA-TMS**	0.36	0.28	0.64	0.9	HH
**ADEP:DAER**	0.1	0.8	81	–10.9	HH
**AAEP:DDER**	ND	>0.95	>360	< –14.5	HH
**AAER:DDEP**	ND	>0.95	>360	< –14.5	HH
**DAER:ADER**	0.35	0.30	0.64	1.1	HH

Mol fractions determined by ^{1}H NMR integration. ND = not detected.

2.3. Model Complexes

2.3.1. [Pd(DA-TMS)$_2$](BF$_4$)$_2$

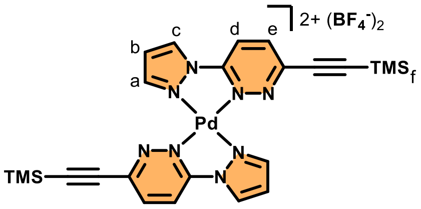

Stock solutions of **DA-TMS** (1.86 mg, 11.3 µmol) and [Pd(MeCN)$_4$](BF$_4$)$_2$ (1.71 mg, 5.67 µmol) were combined in [D$_6$]DMSO for a total volume of 600 µL. The solution was further diluted to 1.88 mM for DOSY NMR experiments. For **[Pd(DA-TMS)(DMSO)$_2$](BF$_4$)$_2$**: ^{1}H NMR (400 MHz, [D$_6$]DMSO, 298 K) δ: 9.44 (1H, d, J = 3.2 Hz, H$_c$), 8.98 (1H, d, J = 9.1 Hz, H$_d$), 8.79 (1H, d, J = 9.1 Hz, H$_e$), 8.66 (1H, d, J = 2.5 Hz, H$_a$), 7.36 (1H, t, J = 2.3 Hz, H$_b$), 0.37 (9H, s, H$_f$). HR ESI-MS (DMSO/MeCN) m/z = 295.0543 [M–(BF$_4$)$_2$]$^{2+}$ (calcd for C$_{24}$H$_{28}$N$_8$PdSi$_2$, 295.0516). D (× 10^{-10} m^2 s^{-1}, 400 MHz, [D$_6$]DMSO, 298 K, [Pd(II)] = 1.88 mM) = 1.25.

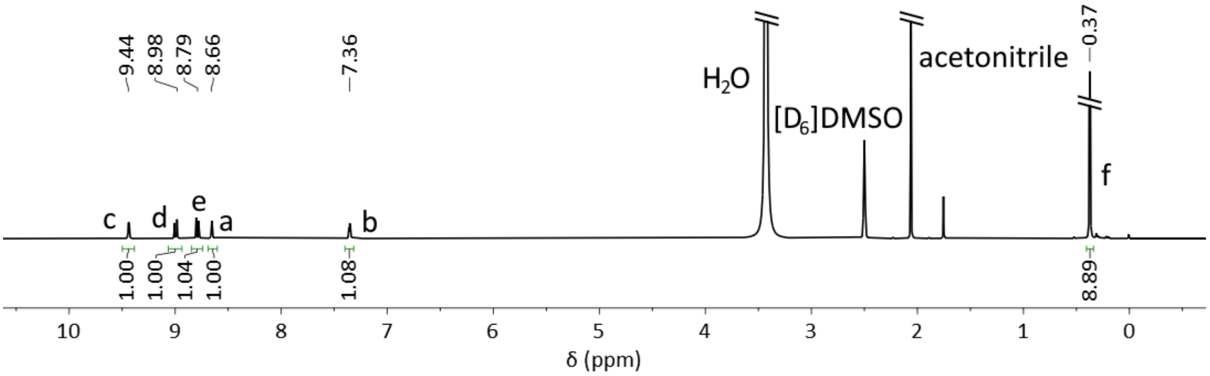

Figure S32. ^{1}H NMR ([D$_6$]DMSO, 400 MHz, 298 K) spectrum of **[Pd(DA-TMS)$_2$](BF$_4$)$_2$.**

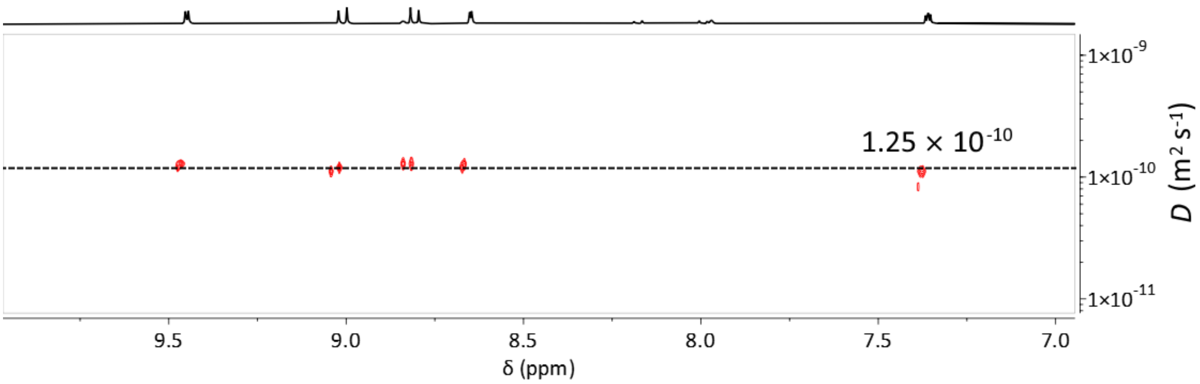

Figure S33. Partial DOSY NMR ([D$_6$]DMSO, 400 MHz, 298 K) spectrum of **[Pd(DA-TMS)$_2$](BF$_4$)$_2$.**

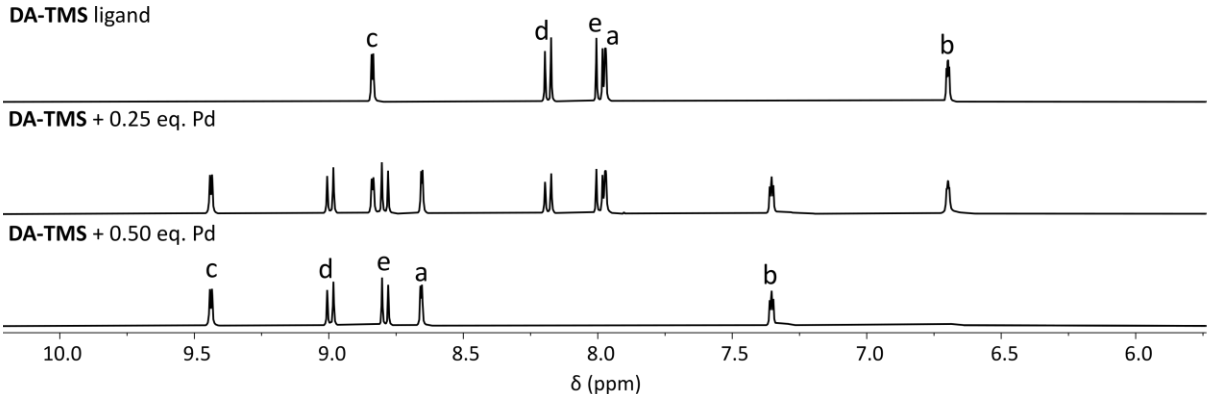

Figure S34. Partial stacked ^{1}H NMR ([D$_6$]DMSO, 400 MHz, 298 K) spectra of **DA-TMS** with increasing equiv. of Pd(II).

2.3.2. [Pd(AD-TMS)$_2$](BF$_4$)$_2$

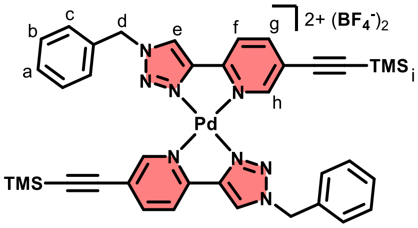

Stock solutions of **AD-TMS** (2.54 mg, 11.3 µmol) and [Pd(MeCN)$_4$](BF$_4$)$_2$ (1.71 mg, 5.67 µmol) were combined in [D$_6$]DMSO for a total volume of 600 µL. The solution was further diluted to 1.88 mM for DOSY NMR experiments. For **[Pd(AD-TMS)(DMSO)$_2$](BF$_4$)$_2$**: ^{1}H NMR (400 MHz, [D$_6$]DMSO, 298 K) δ: 9.54 (1H, s, H$_e$), 9.36 (1H, d, J = 2.2 Hz, H$_h$), 8.64 (1H, dd, J = 8.2 Hz, 1.7 Hz, H$_f$), 8.50 (1H,d, J = 8.4 Hz, H$_g$), 7.60 – 7.57 (2H, m, H$_c$), 7.48 – 7.44 (3H, m, H$_{a,b}$), 6.04 (2H, s, H$_d$), 0.34 (9H, s, H$_i$). HR ESI-MS (DMSO/MeCN) m/z = 385.1004 [M–(BF$_4$)$_2$]$^{2+}$ (calcd for $C_{38}H_{40}N_8PdSi_2$, 385.0985). D (× 10^{-10} m^2 s^{-1}, 400 MHz, [D$_6$]DMSO, 298 K, [Pd(II)] = 1.88 mM) = 1.30.

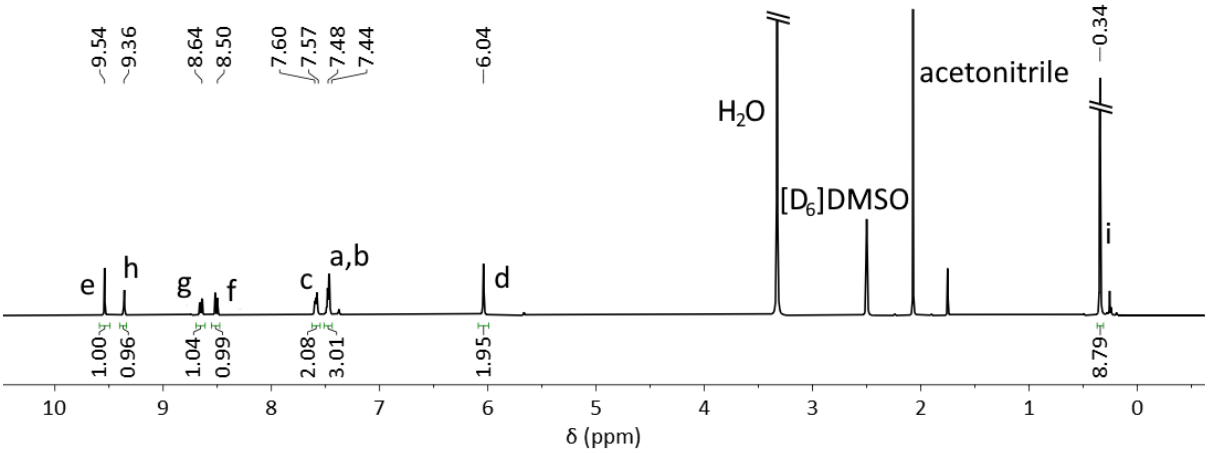

Figure S35. ^{1}H NMR ([D$_6$]DMSO, 400 MHz, 298 K) spectrum of **[Pd(AD-TMS)$_2$](BF$_4$)$_2$.**

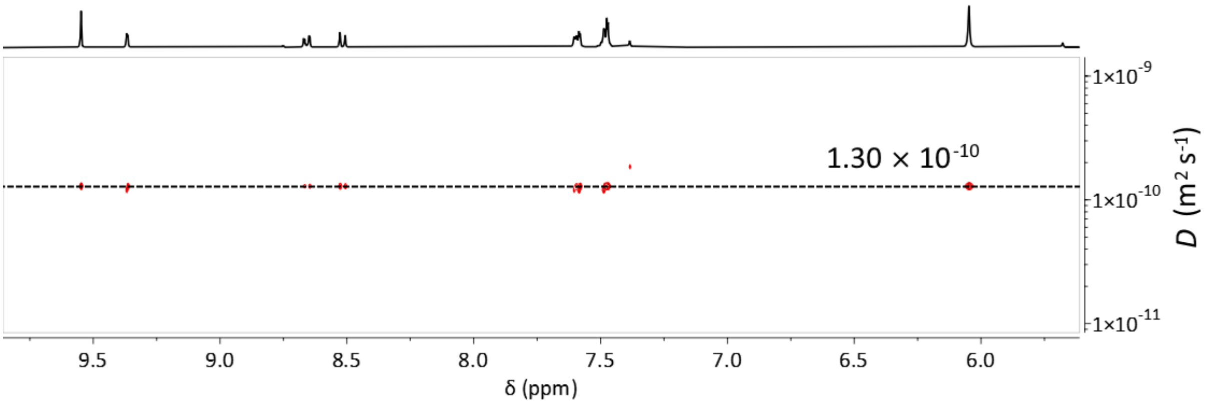

Figure S36. DOSY NMR ([D_6]DMSO, 400 MHz, 298 K) spectrum of **[Pd(AD-TMS)$_2$](BF4)$_2$.**

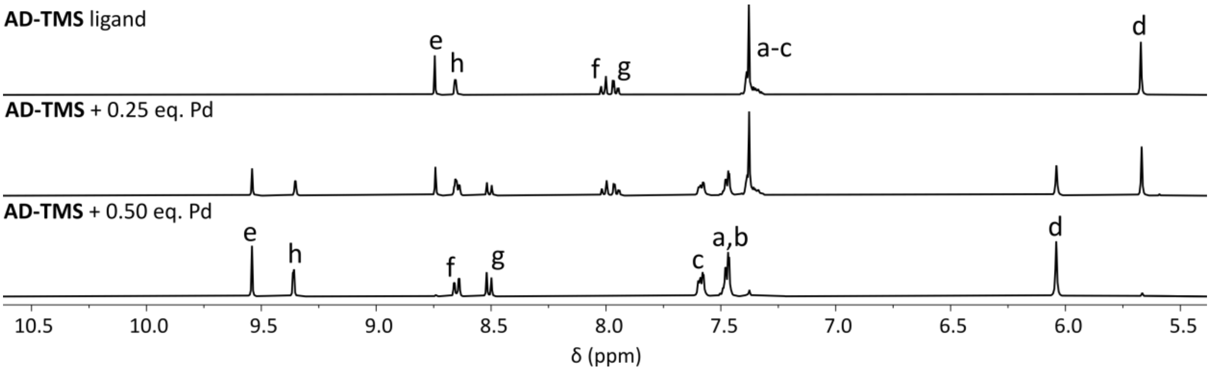

Figure S37. Partial stacked ^{1}H NMR ([D_6]DMSO, 400 MHz, 298 K) spectra of **AD-TMS** with increasing equiv. of Pd(II).

2.3.3. [Pd(AA-TMS)$_2$](BF$_4$)$_2$

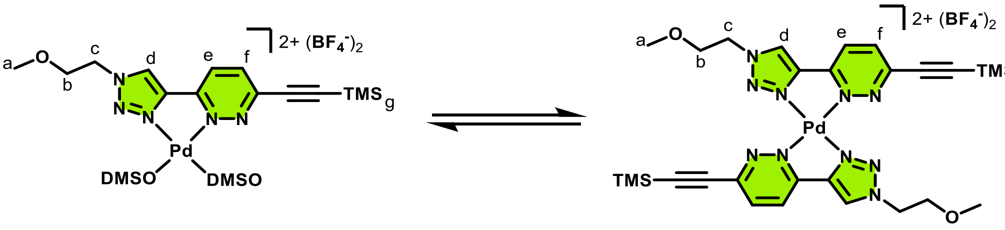

Stock solutions of **AA-TMS** and Pd(MeCN)$_4$(BF$_4$)$_2$ in [D_6]DMSO were combined in 2:1 ratio to 1.88 mM (600 uL) and the mixture analysed by ^{1}H NMR spectroscopy. For **[Pd(AA-TMS)$_2$](BF$_4$)$_2$**: ^{1}H NMR (400 MHz, [D_6]DMSO, 298 K) δ: 9.48 (1H, s, H_d), 8.88 (1H, d, J = 8.7 Hz, H_e), 8.62 (1H, d, J = 8.8 Hz, H_f), 4.97 – 4.94 (2H, m, H_c), 3.99 – 3.97 (2H, m, H_b), methyl peak H_a is coincident with the water peak, 0.36 (9H, s, H_g). HR ESI-MS (DMSO/MeCN) m/z = 354.0817 [M–(BF$_4$)$_2$]$^{2+}$ (calcd for $C_{28}H_{38}N_{10}O_2PdSi_2$, 354.0887). D ($\times$ 10^{-10} m^2 s^{-1}, 400 MHz, [D_6]DMSO, 298 K, [Pd(II)] = 1.88 mM) = 1.39.

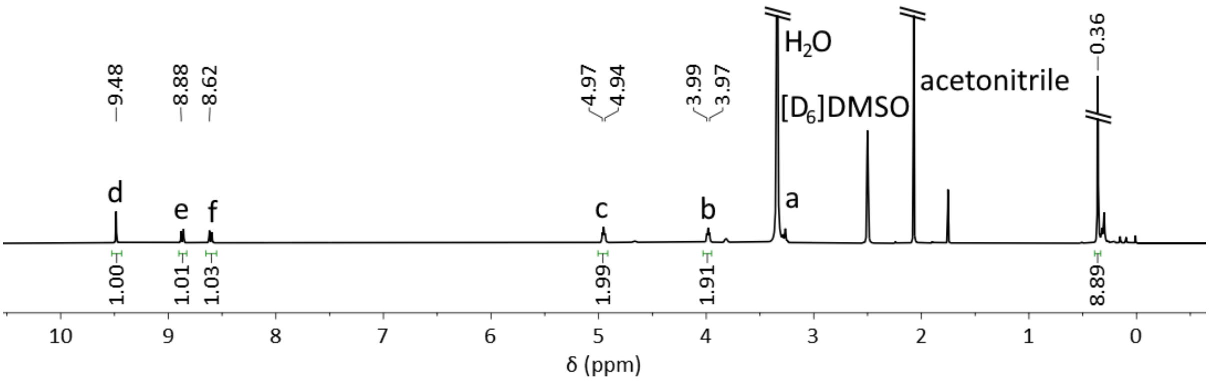

Figure S38. ^1H NMR ([D$_6$]DMSO, 400 MHz, 298 K) spectrum of **[Pd(AA-TMS)$_2$](BF$_4$)$_2$.**

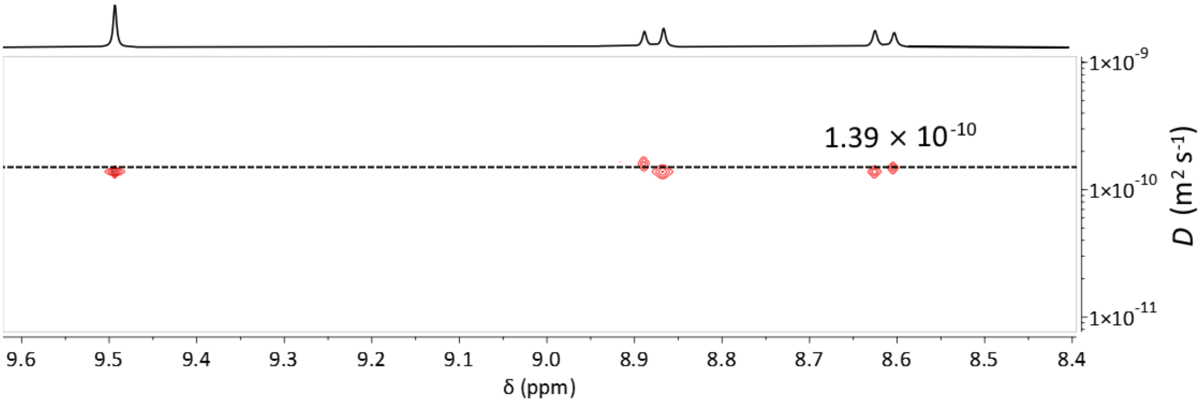

Figure S39. DOSY NMR ([D$_6$]DMSO, 400 MHz, 298 K) spectrum of **[Pd(DA-TMS)$_2$](BF$_4$)$_2$.**

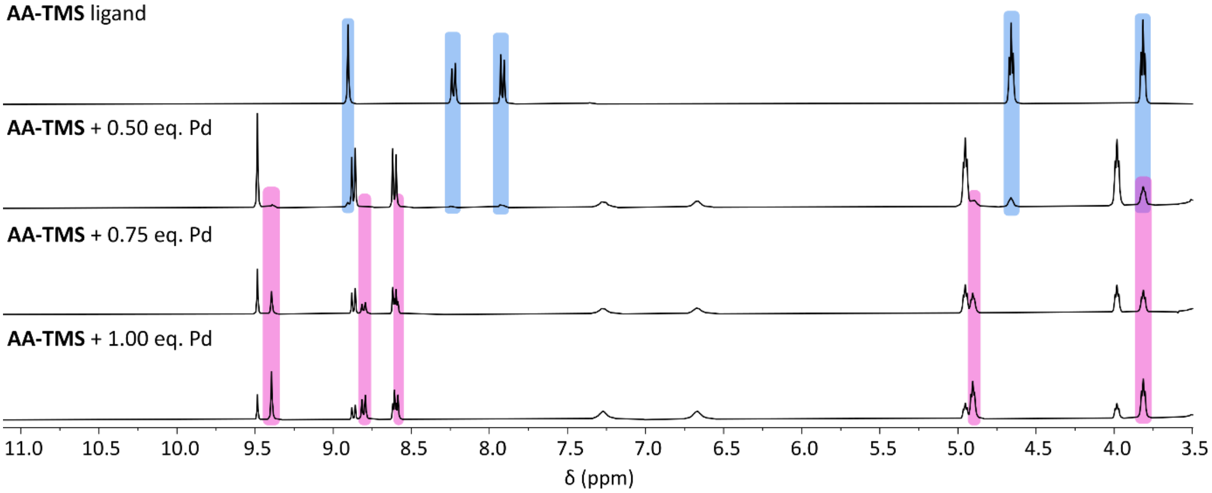

Figure S40. Partial stacked ^1H NMR ([D$_6$]DMSO, 400 MHz, 298 K) spectra of **AA-TMS** with increasing equiv. of Pd(II). Blue highlights the free ligand, and pink highlights **[Pd(AA-TMS)(DMSO)$_2$](BF$_4$)$_2$.**

The addition of excess Pd(II) (2.00 eq. total) saw ≥95% conversion to **[Pd(AA-TMS)(DMSO)$_2$]$^{2+}$** (Figure S40). For **[Pd(AA-TMS)(DMSO)$_2$](BF$_4$)$_2$**: ^1H NMR (400 MHz, [D$_6$]DMSO, 298 K) δ: 9.39 (1H, s, H$_d$), 8.80 (1H, d, J = 8.6 Hz, H$_e$), 8.61 (1H, d, J = 8.8 Hz, H$_f$), 4.92 – 4.89 (2H, m, H$_c$), 3.83 – 3.80 (2H, m, H$_b$), 3.28 (3H, s, H$_a$), 0.32 (9H, s, H$_g$). D (× 10^{-10} m^2 s^{-1}, 400 MHz, [D$_6$]DMSO, 298 K, [Pd(II)] = 1.88 mM) = 1.50.

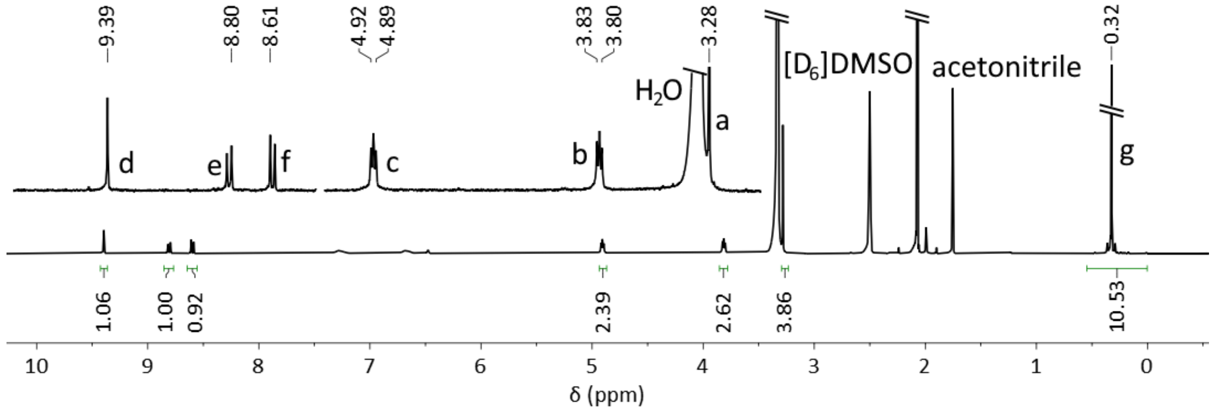

Figure S41. ^{1}H NMR ([D$_6$]DMSO, 400 MHz, 298 K) spectrum of **[Pd(AA-TMS)(DMSO)$_2$](BF$_4$)$_2$.**

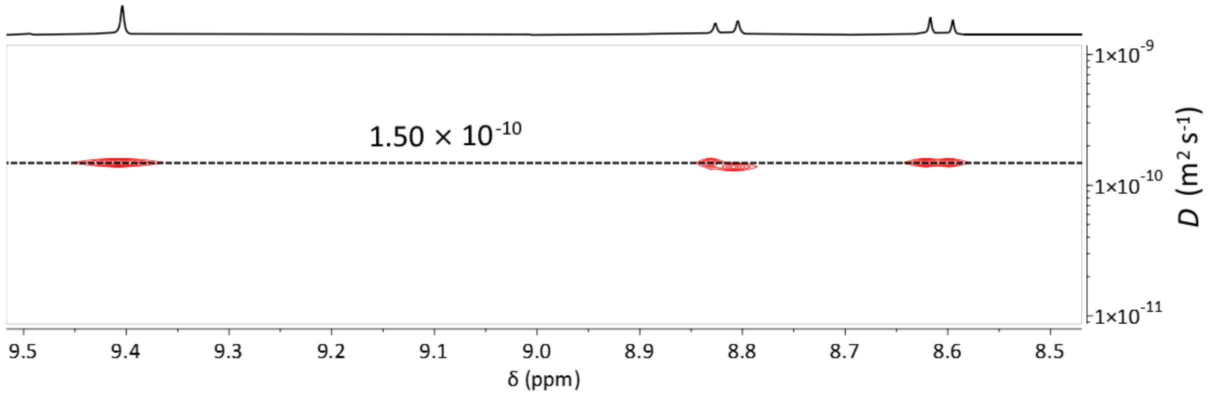

Figure S42. Partial DOSY NMR ([D$_6$]DMSO, 400 MHz, 298 K) spectrum of **[Pd(DA-TMS)(DMSO)$_2$](BF$_4$)$_2$.**

2.3.4. [Pd(DD-TMS)(solvent)$_2$](BF$_4$)$_2$

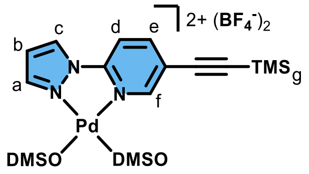

Stock solutions of **DD-TMS** (1.86 mg, 11.3 µmol) and [Pd(MeCN)$_4$](BF$_4$)$_2$ (3.42 mg, 11.3 µmol) were combined in [D$_6$]DMSO for a total volume of 600 µL.. The solution was further diluted to 1.88 mM for DOSY NMR experiments. For **[Pd(DD-TMS)(DMSO)$_2$](BF$_4$)$_2$**: ^{1}H NMR (400 MHz, [D$_6$]DMSO, 298 K) δ: 9.27 (1H, d, J = 3.2 Hz, H$_c$), 8.63 (1H, dd, J = 8.7 Hz, 2.0 Hz, H$_e$), 8.35 (1H, d, J = 8.7 Hz, H$_d$), 8.29 (1H, br, H$_a$), 8.03 (1H, br, H$_f$), 7.10 (1H, t, J = 2.5 Hz), 0.28 (9H, s, H$_g$). The complex was not observed by HR-ESI-MS. D (× 10^{-10} m^2 s^{-1}, 400 MHz, [D$_6$]DMSO, 298 K, [Pd(II)] = 1.88 mM) = 1.80.

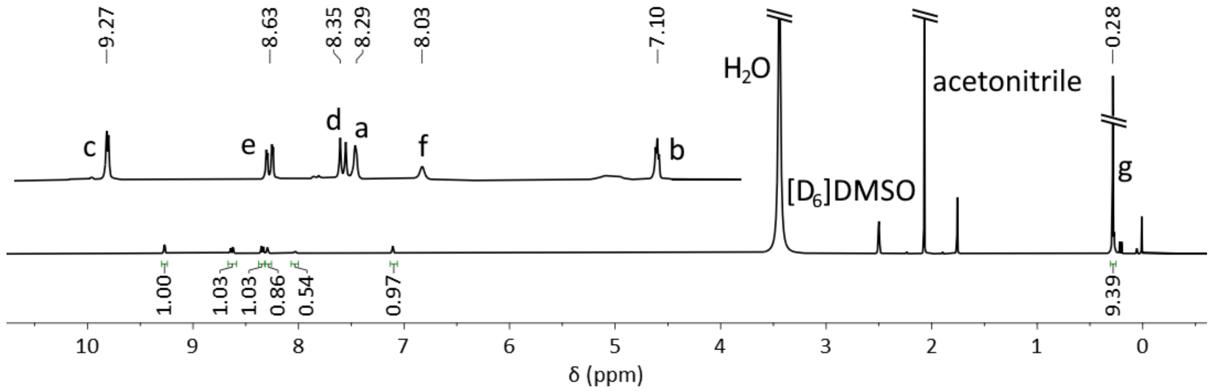

Figure S43. ^{1}H NMR ([D$_6$]DMSO, 400 MHz, 298 K) spectrum of **[Pd(DD-TMS)(DMSO)$_2$](BF$_4$)$_2$.**

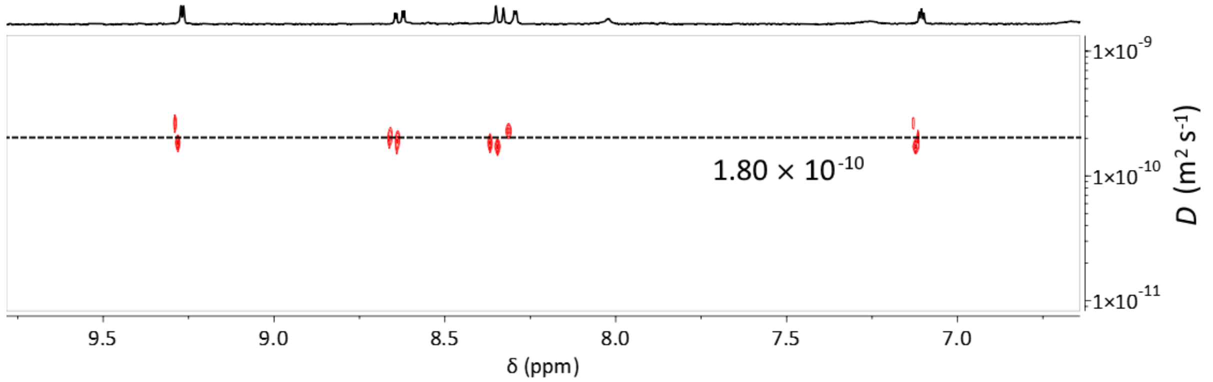

Figure S44. Partial DOSY NMR ([D$_6$]DMSO, 400 MHz, 298 K) spectrum of **[Pd(DD-TMS)(DMSO)$_2$](BF$_4$)$_2$.**

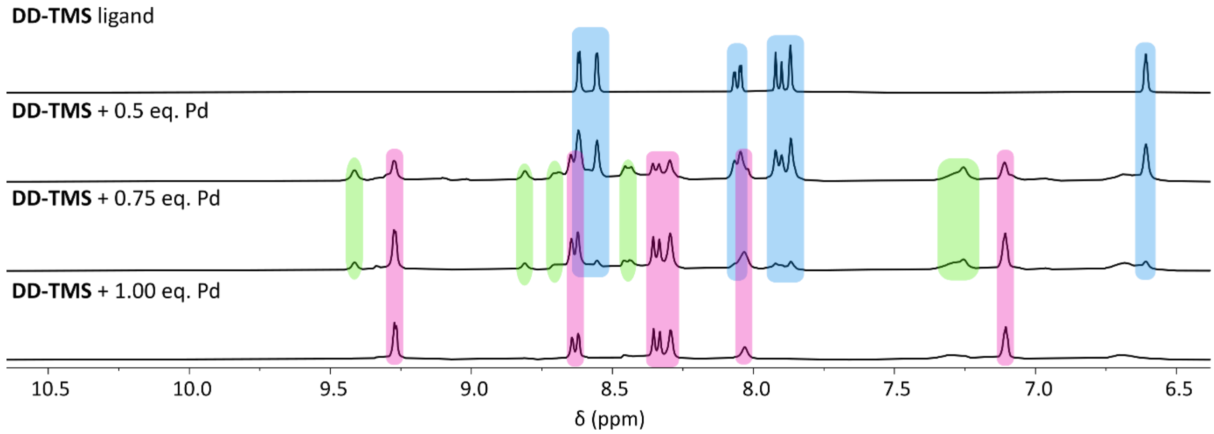

Figure S45. Partial stacked ^{1}H NMR ([D$_6$]DMSO, 400 MHz, 298 K) spectra of **DD-TMS** with increasing equiv. of Pd(II). Blue highlights ligand, pink highlights complex, and green an intermediate species.

2.3.5. Comparison of TMS-protected and terminal-alkyne complexes

To exclude the steric influence of the TMS group on isomer preference, the terminal alkyne ligands (**AA-H**, **DD-H**, **AD-H**, **DA-H**) and their complexes were prepared. Changes in δ upon complexation were similar to the TMS-ligands in all cases and so were assigned as the same isomer species. The ligand **DD-H** decomposed rapidly when exposed to Pd(II) and is not included here.

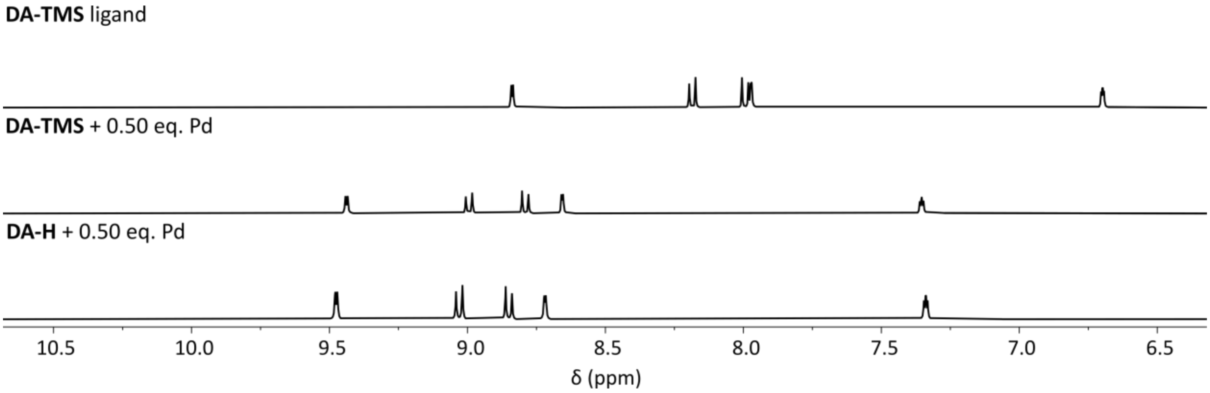

Figure S46. Partial stacked ^{1}H NMR ([D_6]DMSO, 400 MHz, 298 K) spectra of **DA-TMS, $[Pd(DA\text{-}TMS)_2]^{2+}$** and **$[Pd(DA\text{-}H)_2]^{2+}$** with increasing equiv. of Pd(II).

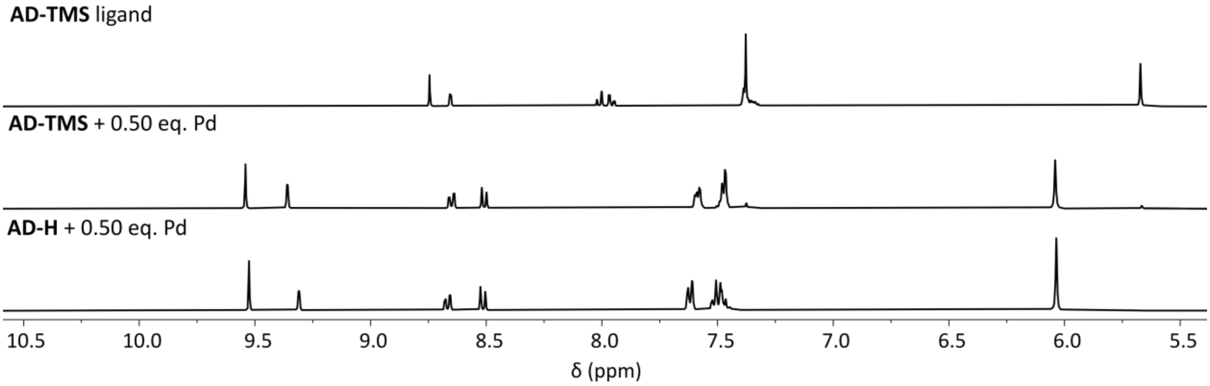

Figure S47. Partial stacked ^{1}H NMR ([D_6]DMSO, 400 MHz, 298 K) spectra of **AD-TMS, $[Pd(AD\text{-}TMS)_2]^{2+}$** and **$[Pd(AD\text{-}H)_2]^{2+}$** with increasing equiv. of Pd(II).

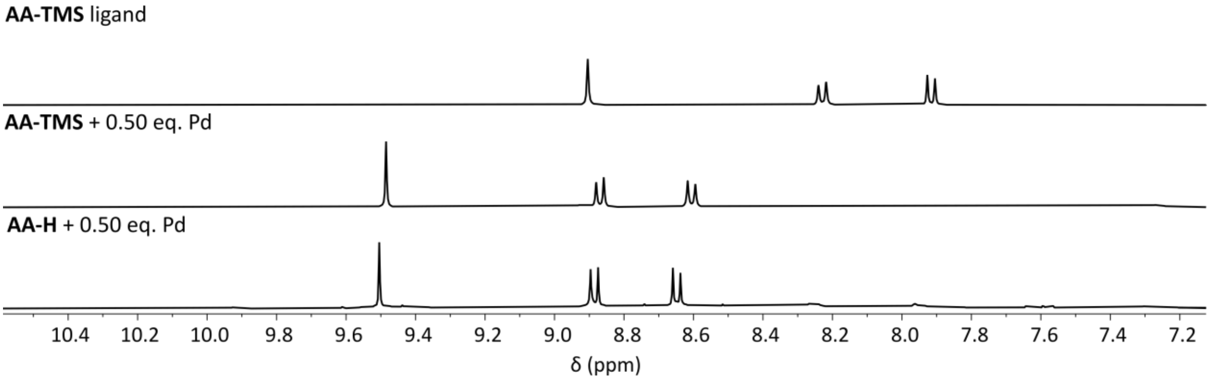

Figure S48. Partial stacked ^{1}H NMR ([D_6]DMSO, 400 MHz, 298 K) spectra of **AA-TMS, $[Pd(AA\text{-}TMS)_2]^{2+}$** and **$[Pd(AA\text{-}H)_2]^{2+}$** with increasing equiv. of Pd(II).

2.3.6. Combining [Pd(DA-TMS)$_2$](BF$_4$)$_2$ and [Pd(AD-TMS)$_2$](BF$_4$)$_2$

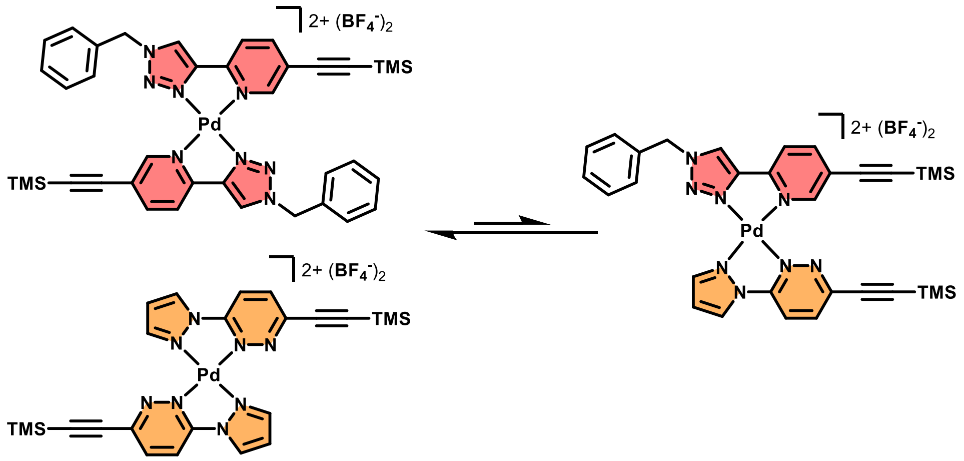

Stock solutions of **DA-TMS**, **AD-TMS** and Pd(II) in [D$_6$]DMSO were combined and diluted to 600 uL (1.88 mM), and the ^{1}H NMR spectrum recorded. HR ESI-MS (DMSO/MeCN) *m/z* = 295.0543 [M–(BF$_4$)$_2$]$^{2+}$ (calcd for $C_{24}H_{28}N_8PdSi_2$, 295.0516); *m/z* = 679.1421 [M–(BF$_4$)$_2$]$^+$ (calcd for $C_{24}H_{28}N_8PdSi_2$, 679.1423). *D* (× 10^{-10} m^2 s^{-1}, 400 MHz, [D$_6$]DMSO, 298 K, [Pd(II)] = 1.88 mM) = 1.45.

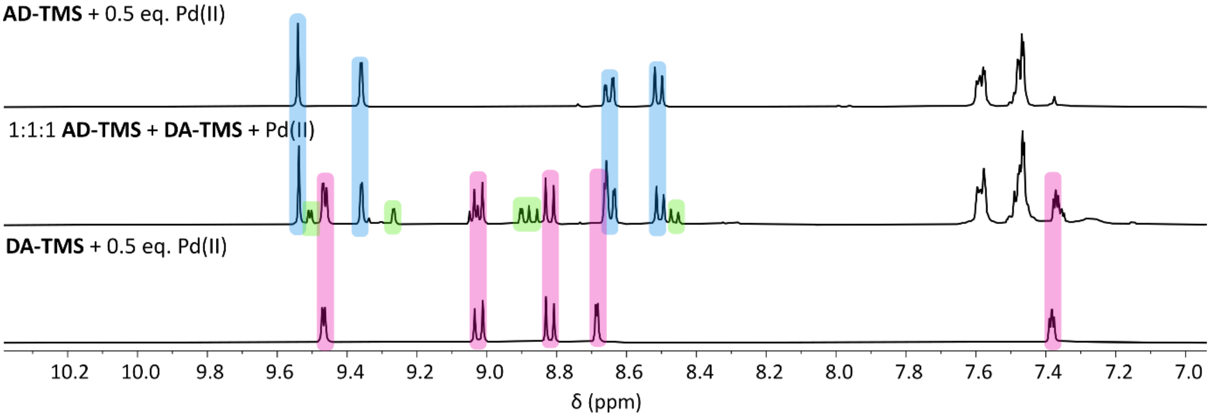

Figure S49. Partial stacked ^{1}H NMR ([D$_6$]DMSO, 400 MHz, 298 K) spectra of 1:2 **AD-TMS** + Pd(II), 1:1:1 **AD-TMS** + **DA-TMS** + Pd(II), and 1:2 DA-**TMS** + Pd(II).Green highlights the new heteroleptic species **[Pd(AD-TMS)(DA-TMS)]$^{2+}$.**

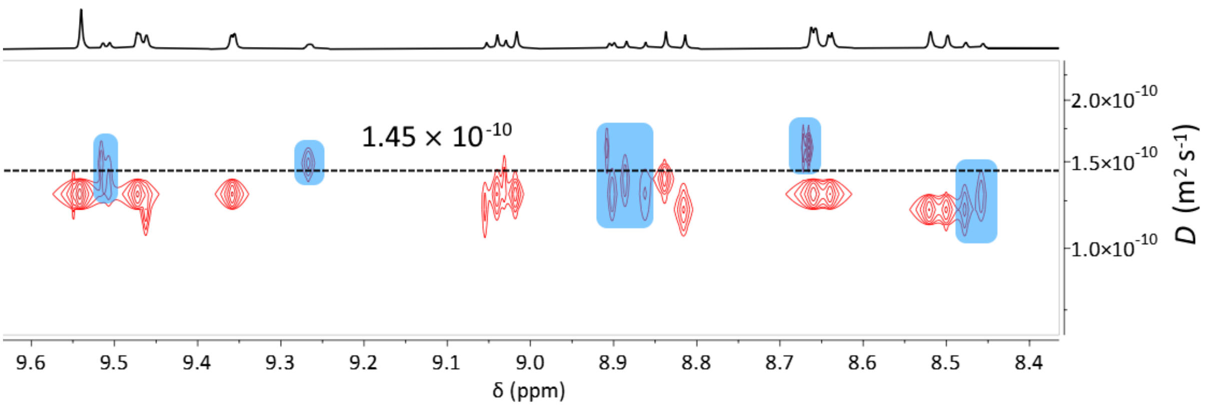

Figure S50. Partial DOSY NMR ([D$_6$]DMSO, 400 MHz, 298 K) spectrum of the mixture of **[Pd(DA-TMS)$_2$], [Pd(AD-TMS)$_2$]** and **[Pd(DA-TMS)(AD-TMS)].** Cross-peaks attributed to **[Pd(DA-TMS)(AD-TMS)]$^{2+}$** are highlighted in blue.

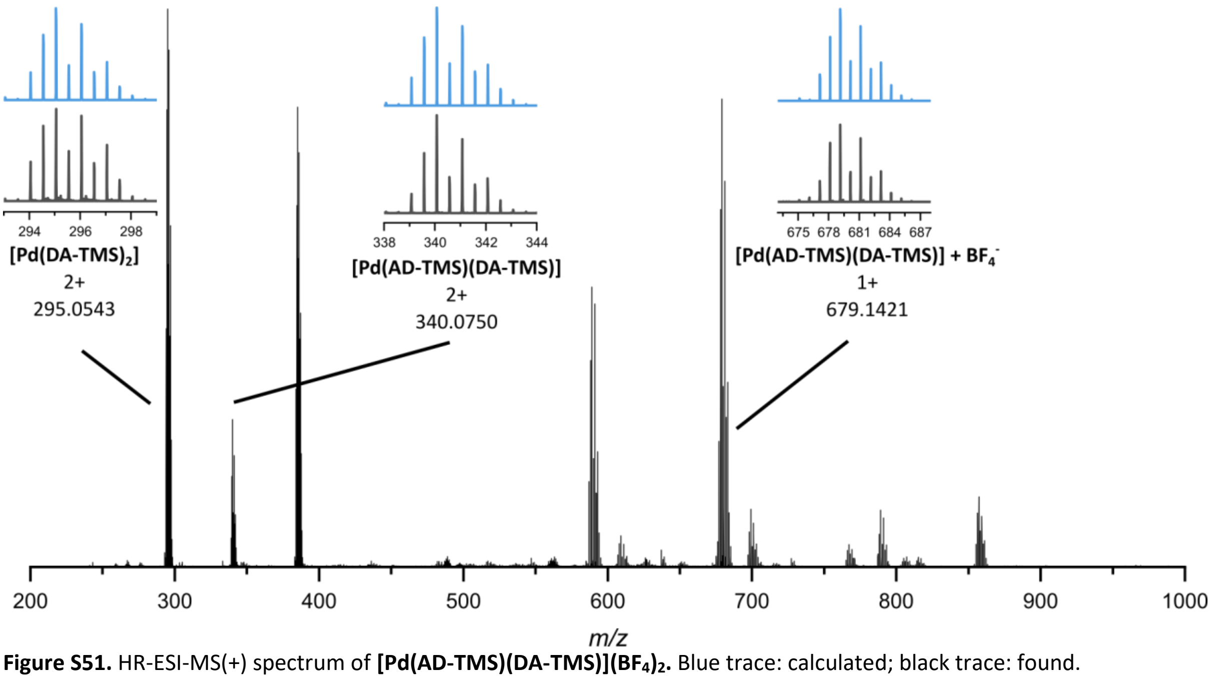

Figure S51. HR-ESI-MS(+) spectrum of **[Pd(AD-TMS)(DA-TMS)](BF_4)$_2$.** Blue trace: calculated; black trace: found.

2.3.7. Combining '[Pd(DD-TMS)$_2$](BF_4)$_2$' and [Pd(AA-TMS)$_2$](BF_4)$_2$

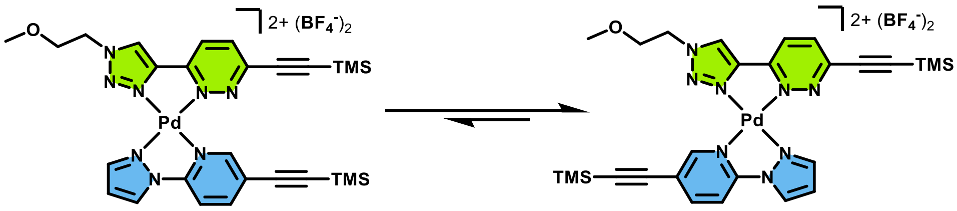

Stock solutions of **DD-TMS**, **AA-TMS** and Pd(II) in [D$_6$]DMSO were combined and diluted to 600 uL (1.88 mM), and the ^{1}H NMR spectrum recorded. The major heteroleptic species of **[Pd(AA-TMS)(DD-TMS)]$^{2+}$** is assigned as HT. HR ESI-MS (DMSO/MeCN) *m/z* = 324.0798 [M–(BF_4)$_2$]$^{2+}$ (calcd for $C_{27}H_{34}N_8OPdSi_2$, 324.0725); *m/z* = 735.1429 [M–(BF_4)]$^+$ (calcd for $C_{27}H_{34}N_8OPdSi_2BF_4$, 735.1437). *D* (× 10^{-10} m^2 s^{-1}, 400 MHz, [D$_6$]DMSO, 298 K, [Pd(II)] = 1.88 mM) = 1.38.

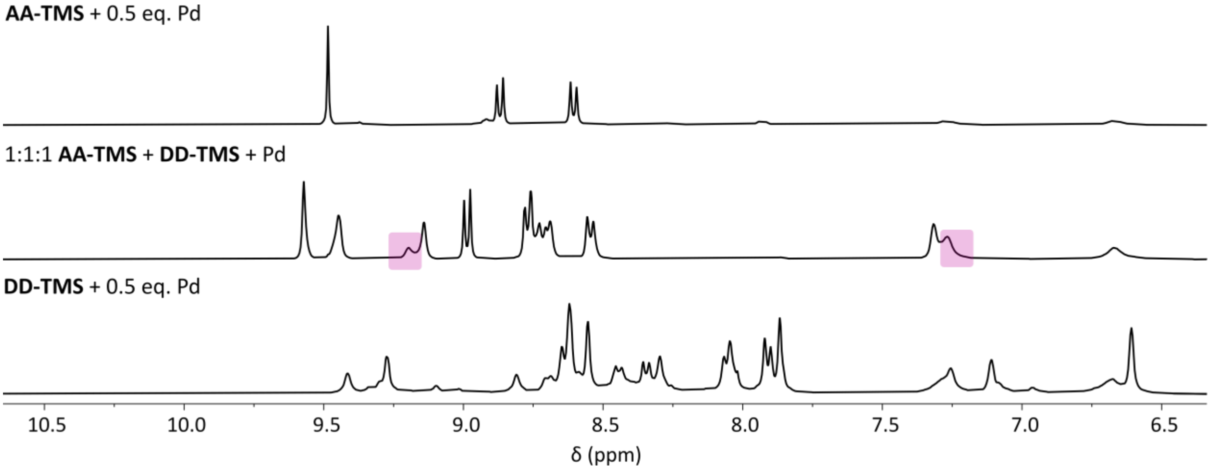

Figure S52. Partial stacked ^{1}H NMR ([D$_6$]DMSO, 400 MHz, 298 K) spectra of 1:2 **AA-TMS** + Pd(II), 1:1:1 **AA-TMS** + **DD-TMS** + Pd(II), and 1:2 **DD-TMS + Pd(II).** Pink highlights signals attributed to the minor species (assigned as HH).

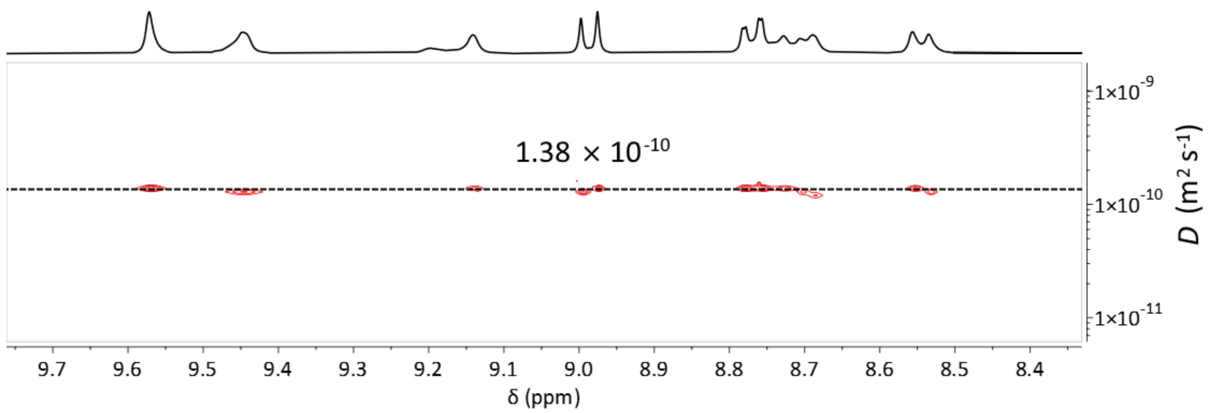

Figure S53. Partial DOSY NMR ([D$_6$]DMSO, 400 MHz, 298 K) spectrum o**f [Pd(AA-TMS)(DD-TMS)](BF_4)$_2$.**

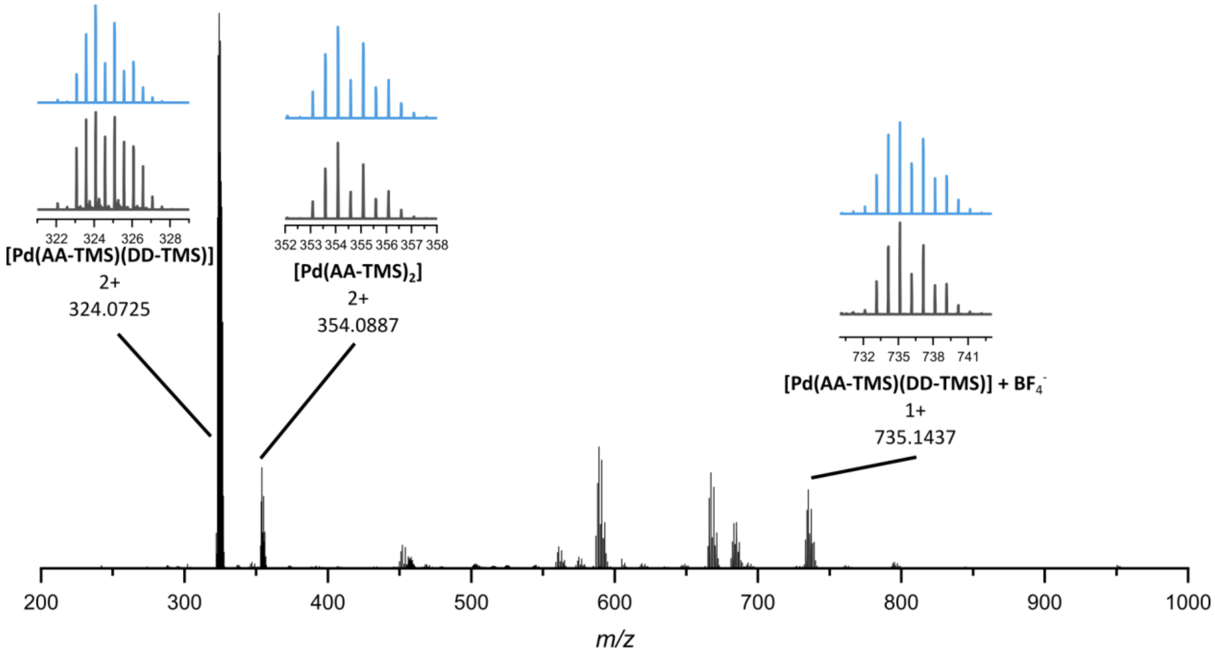

Figure S54. HR-ESI-MS(+) spectrum of **[Pd(AA-TMS)(DD-TMS)](BF_4)$_2$.** Blue trace: calculated; black trace: found.

2.4. Homoleptic Complexes

2.4.1. [Pd(AAER)$_2$](BF$_4$)$_2$

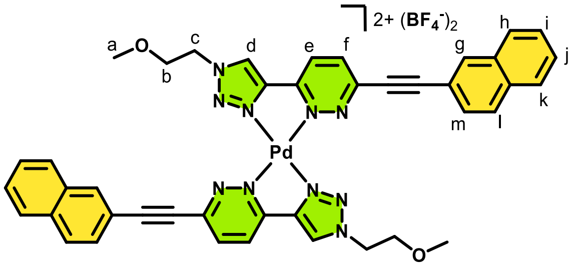

Stock solutions of **AAER** and Pd(MeCN)$_4$(BF$_4$)$_2$ in [D$_6$]DMSO were combined in 2:1 ratio to 1.88 mM (600 uL) and the mixture analysed by ^{1}H NMR spectroscopy. For **[Pd(AAER)$_2$](BF$_4$)$_2$:** ^{1}H NMR (400 MHz, [D$_6$]DMSO, 298 K) δ: 9.54 (s, 1H, H), 8.95 (d, J = 8.7 Hz, 1H, H$_e$), 8.76 (d, J = 8.7 Hz, 1H, H$_f$), 8.64–7.63 (m (br), 7H, H$_{g\text{-}l}$), 5.06 (t, J = 7.0 Hz, 2H, H$_c$), 4.02 (t, J = 7.0 Hz, 2H, H$_b$), 3.36 (s, 3H, H$_a$). HR ESI-MS (DMSO/MeCN) m/z = 408.0957 [M–(BF$_4$)$_2$]$^{2+}$ (calcd for $C_{42}H_{34}N_{10}O_2Pd$, 408.0958); m/z = 903.1948 [M–(BF$_4$)]$^+$ (calcd for $BC_{42}H_{35}N_{10}Pd$, 903.1951). D (× 10^{-10} m^2 s^{-1}, 400 MHz, [D$_6$]DMSO, 298 K, [Pd(II)] = 1.88 mM) = 1.22.

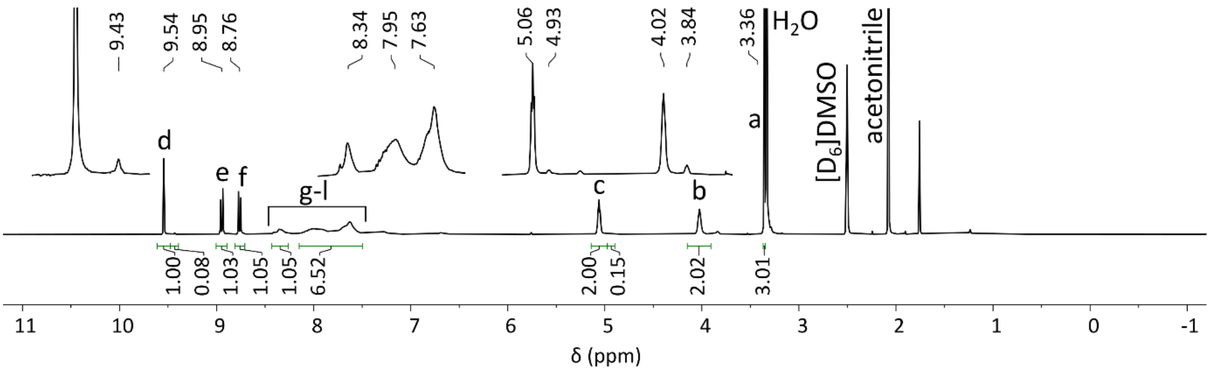

Figure S55. ^{1}H NMR ([D$_6$]DMSO, 400 MHz, 298 K) spectrum of **[Pd(AAER)$_2$](BF$_4$)$_2$.**

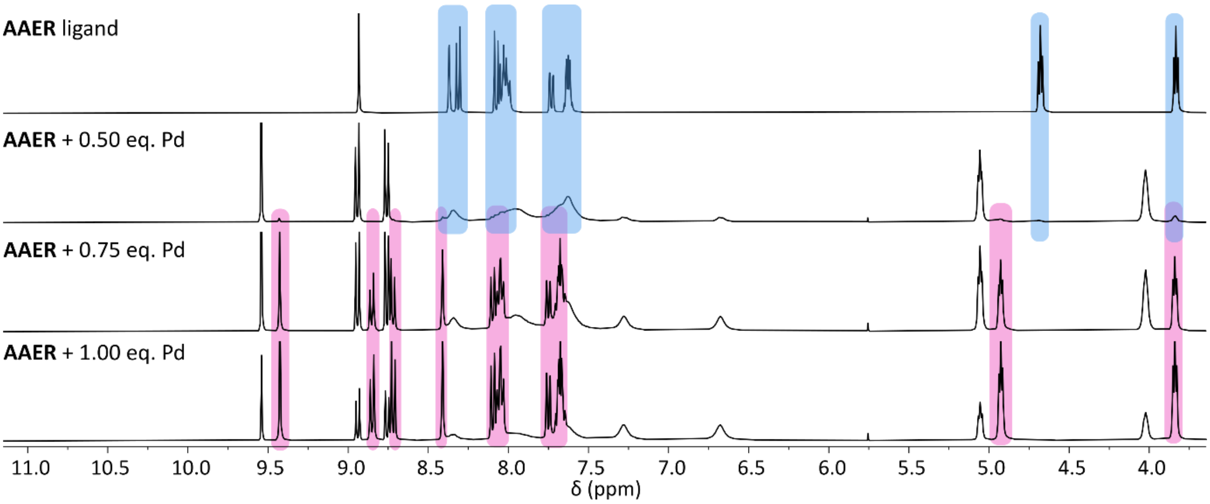

Figure S56. Partial stacked ^{1}H NMR ([D$_6$]DMSO, 400 MHz, 298 K) spectra of **AAER** with increasing equiv. of Pd(II). Blue highlights the free ligand, and pink highlights **[Pd(AAER)(DMSO)$_2$]$^{2+}$.**

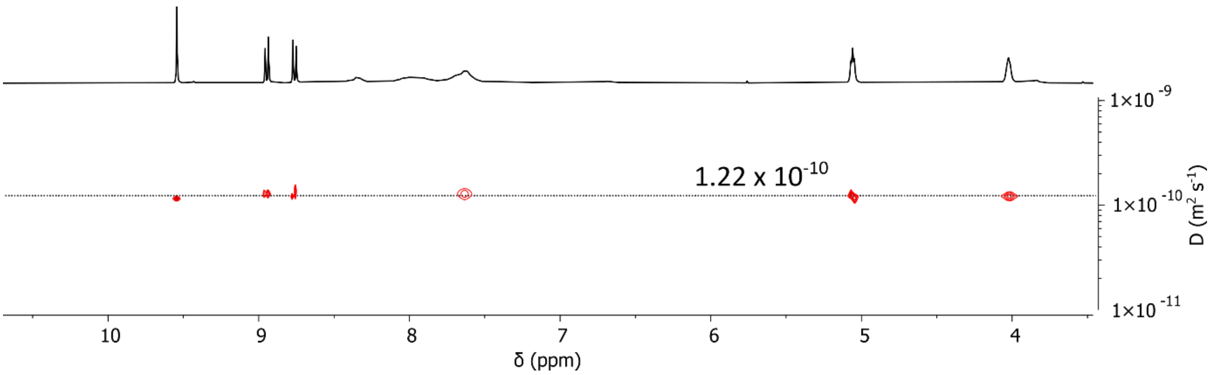

Figure S57. Partial DOSY NMR ([D$_6$]DMSO, 400 MHz, 298 K) spectrum of **[Pd(AAER)$_2$](BF$_4$)$_2$.**

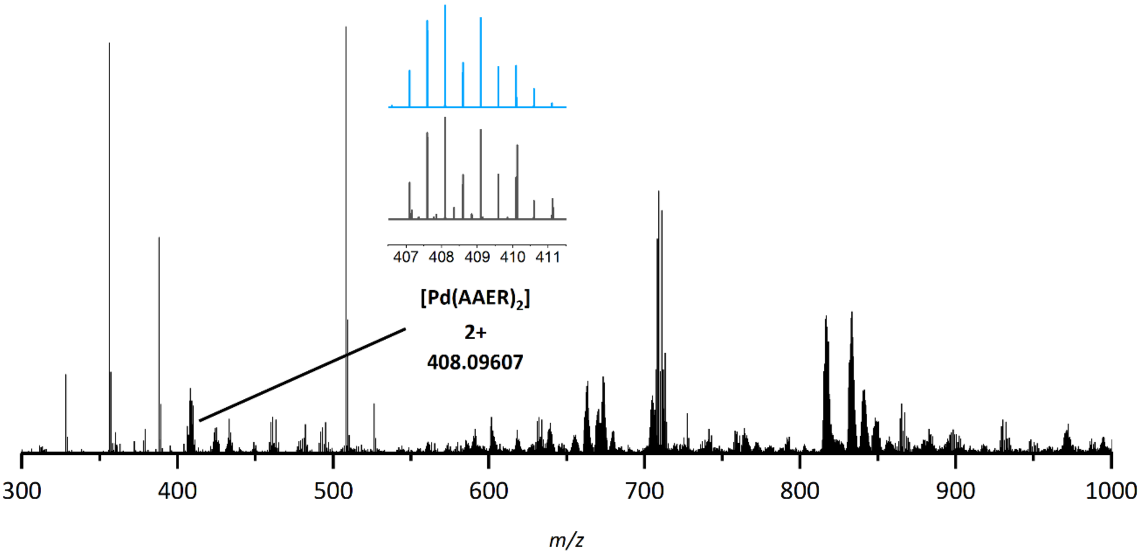

Figure S58. HR-ESI-MS(+) spectrum of **[Pd(AAER)$_2$](BF$_4$)$_2$.** Blue trace: calculated; black trace: found.

To probe the effect of concentration on intermolecular π-π stacking, a solution of **[Pd(AAER)$_2$]$^{2+}$** in [D$_6$]DMSO was diluted 8-fold and then 4-fold. This dilution shifts the equilibrium towards **[Pd(AAER)(DMSO)$_2$]$^{2+}$**, but importantly causes some sharpening of the broad signals, consistent with reduced intermolecular π-π interactions at low concentrations.

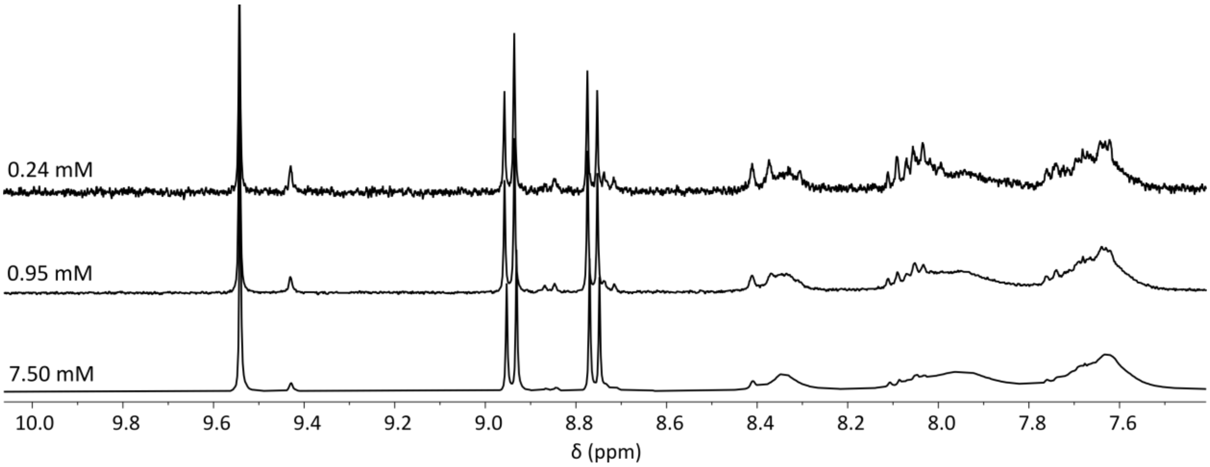

Figure S59. Partial stacked ^{1}H NMR ([D$_6$]DMSO, 400 MHz, 298 K) spectra of **[Pd(AAER)$_2$]$^{2+}$** at various concentrations.

2.4.2. $[Pd(AAEP)_2](BF_4)_2$

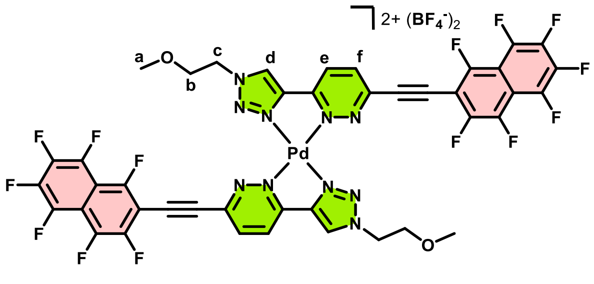

Stock solutions of **AAEP** and $Pd(MeCN)_4(BF_4)_2$ in [D$_6$]DMSO were combined in 2:1 ratio to 1.88 mM (600 uL) and the mixture analysed by ^{1}H NMR spectroscopy. For **$[Pd(AAEP)_2](BF_4)_2$:** ^{1}H NMR (400 MHz, [D$_6$]DMSO, 298 K) δ: 9.59 (s, 1H, H$_d$), 9.03 (d, J = 8.7 Hz, 1H, H$_e$), 8.89 (d, J = 8.7 Hz, 1H, H$_f$), 5.02 (t, J = 7.0 Hz, 2H, H$_c$), 3.98 (t, J = 7.0 Hz, 2H, H$_b$), 3.35 (s, 3H, H$_a$). ^{19}F NMR (400 MHz, $CDCl_3$, 298 K) δ: -112.1 (m), -134.5 (m), -143.9 (dt, J = 68, 17 Hz), -146.3 (dt, J = 58, 22 Hz), -148.3 (s), -150.6 (d, J = 2.2 Hz), -154.3(br). HR ESI-MS (DMSO/MeCN) m/z = 565.0444 $[M-(BF_4)_2]^{2+}$ (calcd for $C_{52}H_{22}F_{14}N_8Pd$, 565.0384); m/z = 1217.0879 $[M-(BF_4)]^+$ (calcd for $BC_{52}H_{22}F_{18}N_8Pd$, 1217.0803). D ($\times$ 10^{-10} m^2 s^{-1}, 400 MHz, [D$_6$]DMSO, 298 K, [Pd(II)] = 1.88 mM) = 1.27.

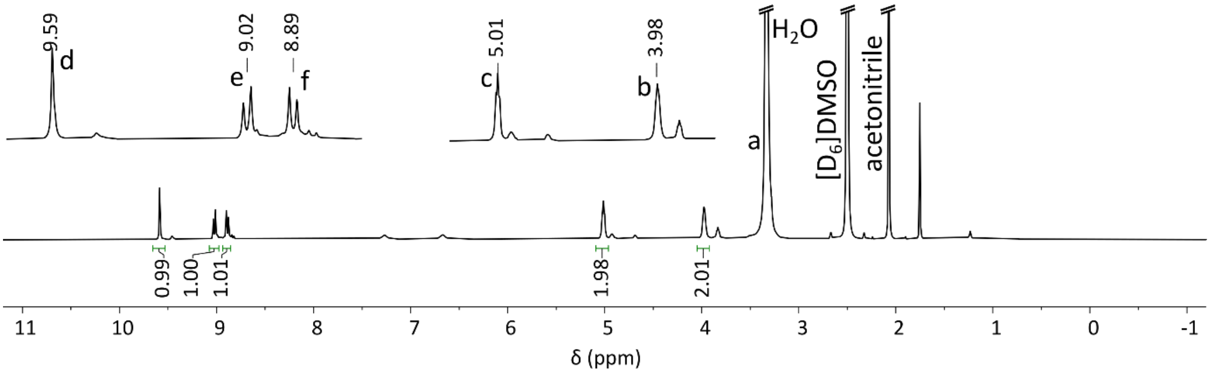

Figure S60. ^{1}H NMR ([D$_6$]DMSO, 400 MHz, 298 K) spectrum of **$[Pd(AAEP)_2](BF_4)_2$.**

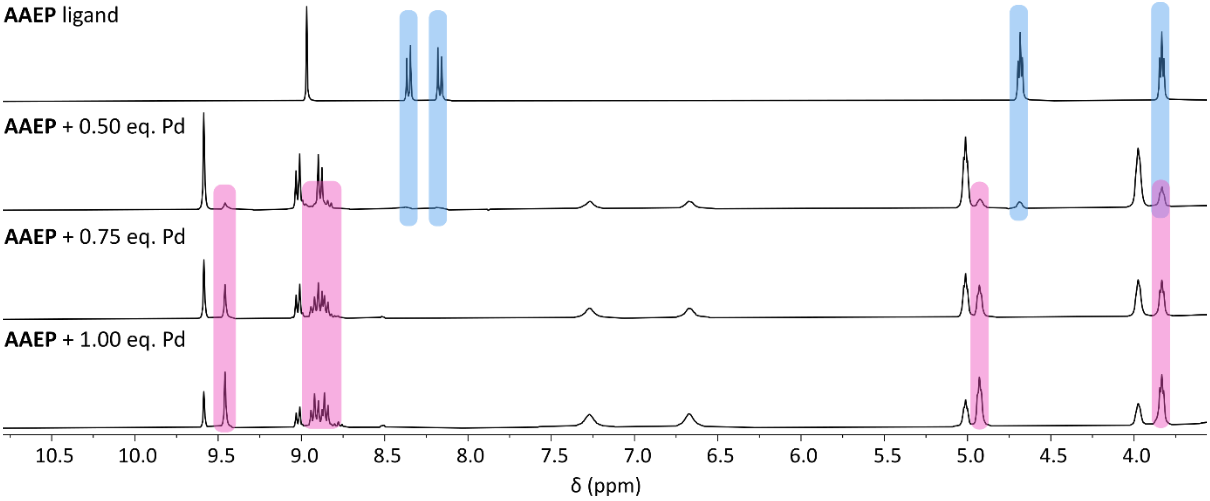

Figure S61. Partial stacked ^{1}H NMR ([D$_6$]DMSO, 400 MHz, 298 K) spectra of **AAEP** with increasing equiv. of Pd(II). Blue highlights the free ligand, and pink highlights **$[Pd(AAEP)(DMSO)_2](BF_4)_2$.**

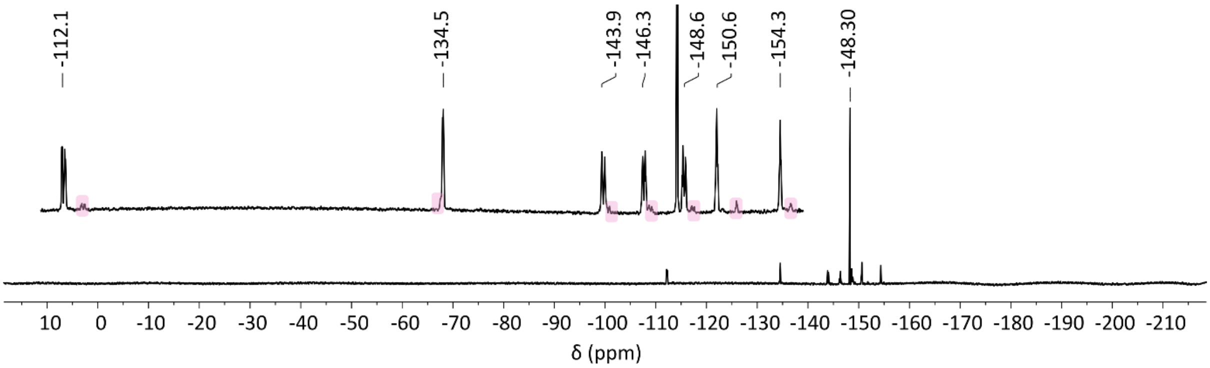

Figure S62. ^{19}F NMR ([D$_6$]DMSO, 376 MHz, 298 K) spectrum of **[Pd(AAEP)$_2$](BF$_4$)$_2$.** Pink highlights the minor species.

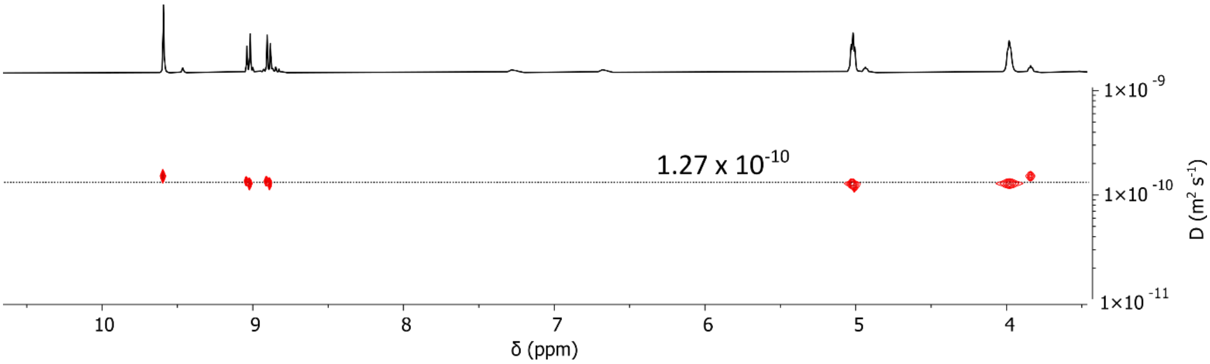

Figure S63. Partial DOSY NMR ([D$_6$]DMSO, 400 MHz, 298 K) spectrum of **[Pd(AAEP)$_2$]$^{2+}$.** Minor species were not observed.

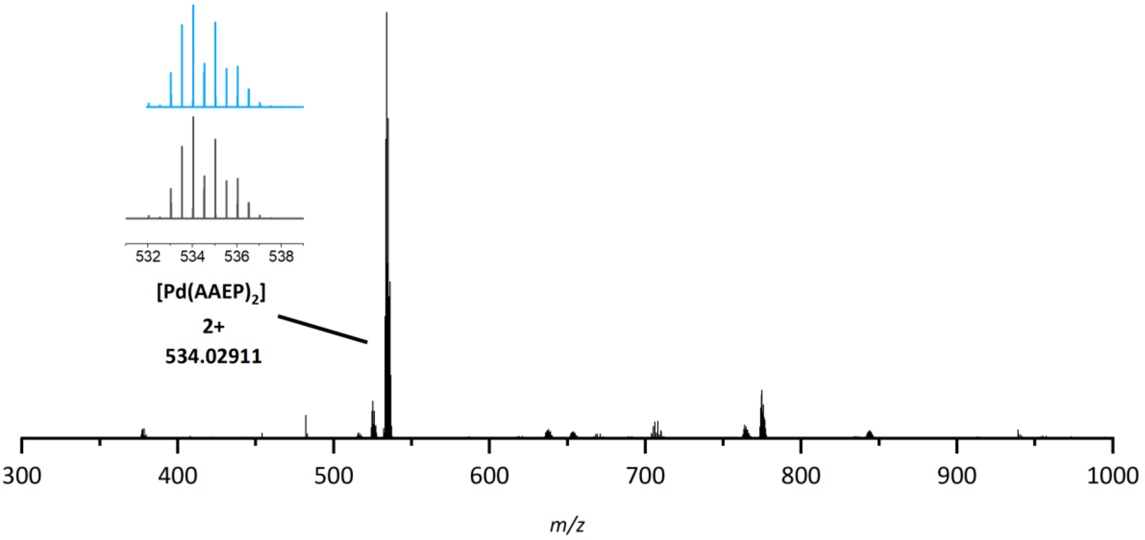

Figure S64. HR-ESI-MS(+) spectrum of **[Pd(AAEP)$_2$](BF$_4$)$_2$.** Blue trace: calculated; black trace: found.

2.4.3. $[Pd(ADER)_2](BF_4)_2$

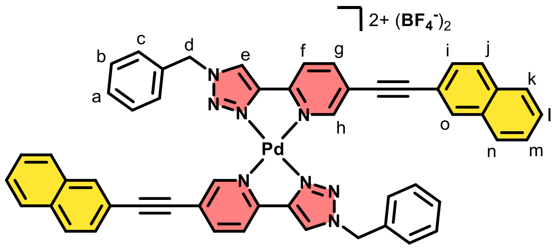

Stock solutions of **ADER** and $Pd(MeCN)_4(BF_4)_2$ in [D$_6$]DMSO were combined in 2:1 ratio to 1.88 mM (600 uL) and the mixture analysed by ^{1}H NMR spectroscopy. For **$[Pd(ADER)_2](BF_4)_2$:** ^{1}H NMR (400 MHz, [D$_6$]DMSO, 298 K) δ: 9.57 (s, 2H, H_e), 9.46 (dd, J = 1.7, 0.6 Hz, 2H, H_h), 8.78 (dd, J = 8.1, 1.7 Hz, 2H, H_f), 8.59 (dd, J = 8.1, 0.6 Hz, 2H, H_g), 8.38 (t, J = 1.1 Hz, 2H, H_i), 8.08 (d, J = 8.6 Hz, 2H, H_o), 8.03–7.97 (m, 2H, $H_{j,m}$), 7.76 (dd, J = 8.4, 1.7 Hz, 2H, H_n), 7.71–7.65 (m, 2H, H_c), 7.65–7.59 (m, 2H, $H_{k,l}$), 7.41–7.36 (m 2H, H_b), 6.14 (s, 2H, H_d). HR ESI-MS (DMSO/MeCN) m/z = 439.1084 $[M–(BF_4)_2]^{2+}$ (calcd for $C_{52}H_{36}N_8Pd$, 439.1044); m/z = 965.2166 $[M–(BF_4)]^+$ (calcd for B $C_{52}H_{36}N_8F_3Pd$, 965.2127). D ($\times 10^{-10}$ m^2 s^{-1}, 400 MHz, [D$_6$]DMSO, 298 K, [Pd(II)] = 1.88 mM) = 1.15.

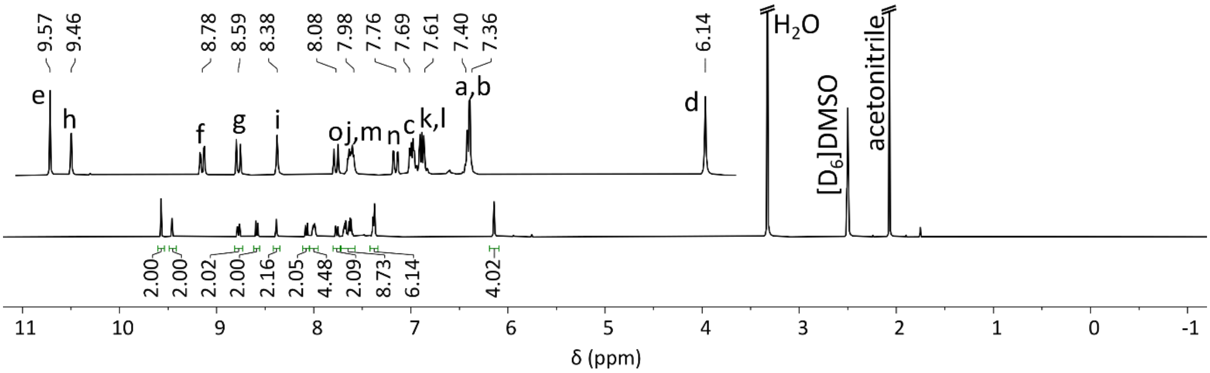

Figure S65. ^{1}H NMR ([D$_6$]DMSO, 400 MHz, 298 K) spectrum of **$[Pd(ADER)_2](BF_4)_2$.**

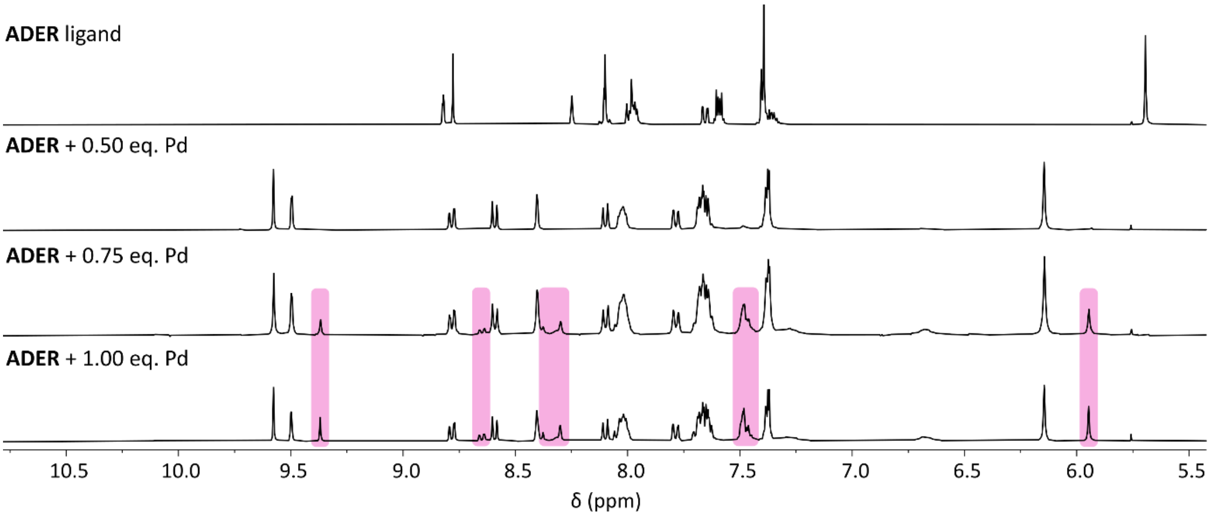

Figure S66. Partial stacked ^{1}H NMR ([D$_6$]DMSO, 400 MHz, 298 K) spectra of **ADER** with increasing equiv. of Pd(II). Pink highlights **$[Pd(ADER)(DMSO)_2](BF_4)_2$.**

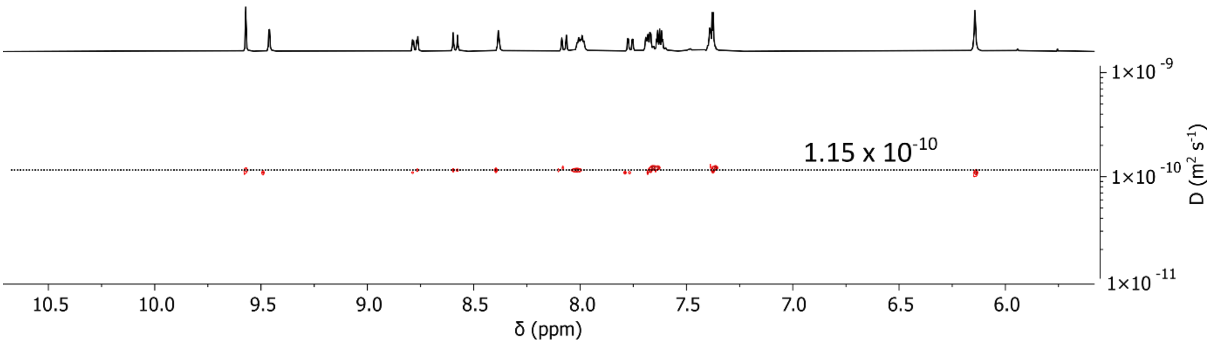

Figure S67. Partial DOSY NMR ([D$_6$]DMSO, 400 MHz, 298 K) spectrum of **[Pd(ADER)$_2$](BF$_4$)$_2$.**

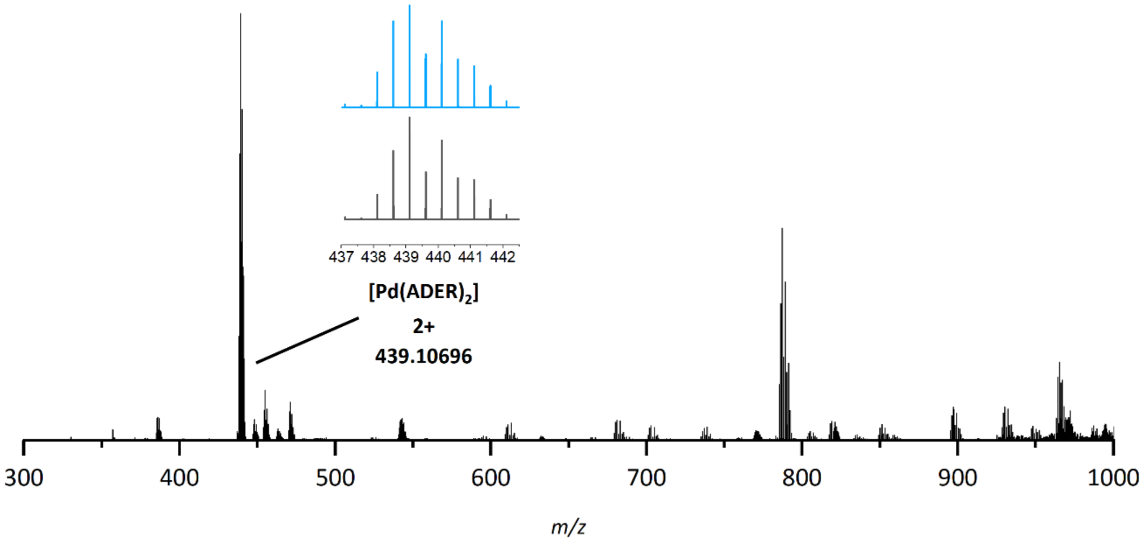

Figure S68. HR-ESI-MS(+) spectrum of **[Pd(ADER)$_2$](BF$_4$)$_2$.** Blue trace: calculated; black trace: found.

[Pd(ADEP)$_2$](BF$_4$)$_2$

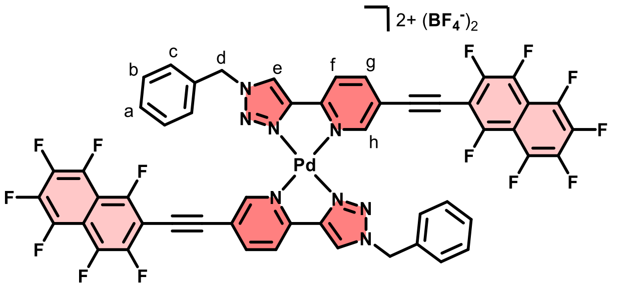

Stock solutions of **ADEP** and Pd(MeCN)$_4$(BF$_4$)$_2$ in [D$_6$]DMSO were combined in 2:1 ratio to 1.88 mM (600 uL) and the mixture analysed by ^{1}H NMR spectroscopy. For **[Pd(ADEP)$_2$](BF$_4$)$_2$**:^{1}H NMR (400 MHz, [D$_6$]DMSO, 298 K) δ: 9.64 (s, 1H, H$_e$), 9.56 (d, J = 1.8 Hz, 1H, H$_h$), 8.89 (dd, J = 8.3, 1.8 Hz, 1H, H$_f$), 8.64 (d, J = 8.3 Hz, 1H, H$_g$), 7.61 (dd, J = 8.0, 2.2 Hz, 2H, H$_c$), 7.47–7.38 (m, 3H, H$_{a,b}$), 6.07 (s, 2H, H$_d$). ^{19}F NMR (400 MHz, CDCl$_3$, 298 K) δ: -111.1 (dd, J = 65.7, 17.4 Hz), -134.3 (d, J = 18.9 Hz), -143.5 (dt, J = 65.8, 17.5 Hz), -146.2 (dt, J = 56.9, 17.2 Hz), -148.3 (s, 4F), -148.3 (d, J = 2.2 Hz), -148.6 (dt, J = 57.2, 18.5 Hz), -149.9 (t, J = 20.3 Hz), -154.1 (t, J = 22.0 Hz). HR ESI-MS (DMSO/MeCN) m/z = 565.0444 [M–(BF$_4$)$_2$]$^{2+}$ (calcd for C$_{52}$H$_{22}$F$_{14}$N$_8$Pd, 565.0393); m/z = 1217.0879 [M–(BF$_4$)]$^+$ (calcd for BC$_{52}$H$_{22}$F$_{18}$N$_8$Pd, 1217.0490). D (× 10^{-10} m^2 s^{-1}, 400 MHz, [D$_6$]DMSO, 298 K, [Pd(II)] = 1.88 mM) = 1.16.

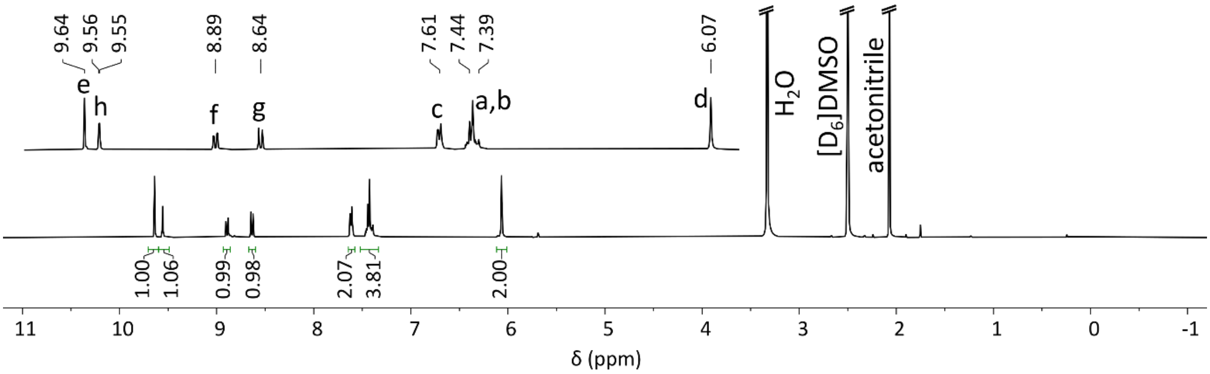

Figure S69. ^{1}H NMR ([D$_6$]DMSO, 400 MHz, 298 K) spectrum of **[Pd(ADEP)$_2$](BF$_4$)$_2$.**

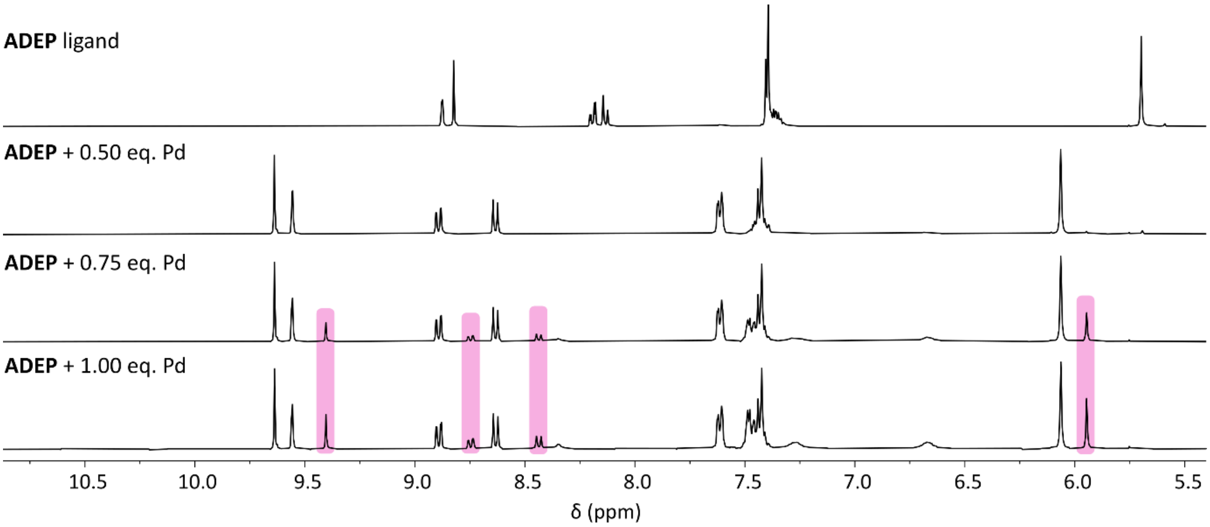

Figure S70. Partial stacked ^{1}H NMR ([D$_6$]DMSO, 400 MHz, 298 K) spectra of **ADEP** with increasing equiv. of Pd(II). Pink highlights [**Pd(AA-TMS)(DMSO)$_2$](BF$_4$)$_2$.**

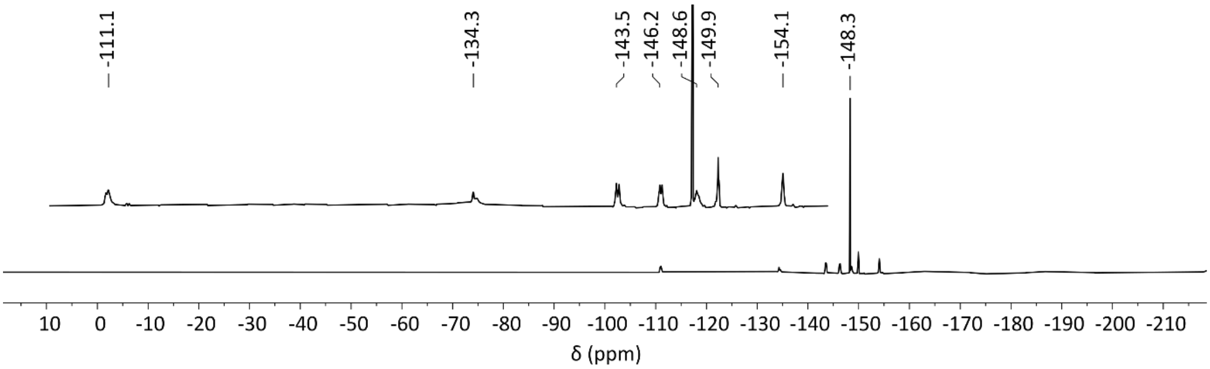

Figure S71. ^{19}F NMR ([D$_6$]DMSO, 376 MHz, 298 K) spectrum of **[Pd(ADEP)$_2$](BF$_4$)$_2$.**

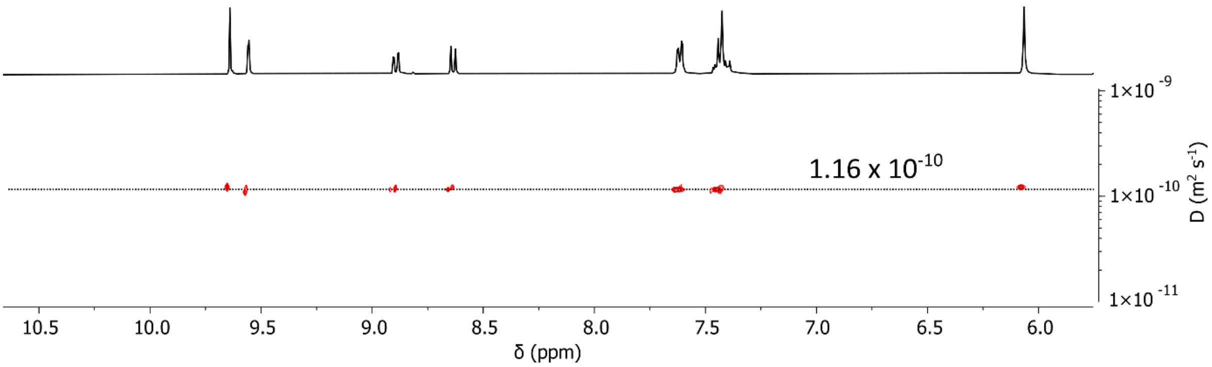

Figure S72. Partial DOSY NMR ([D_6]DMSO, 400 MHz, 298 K) spectrum of **[Pd(ADEP)$_2$](BF$_4$)$_2$.**

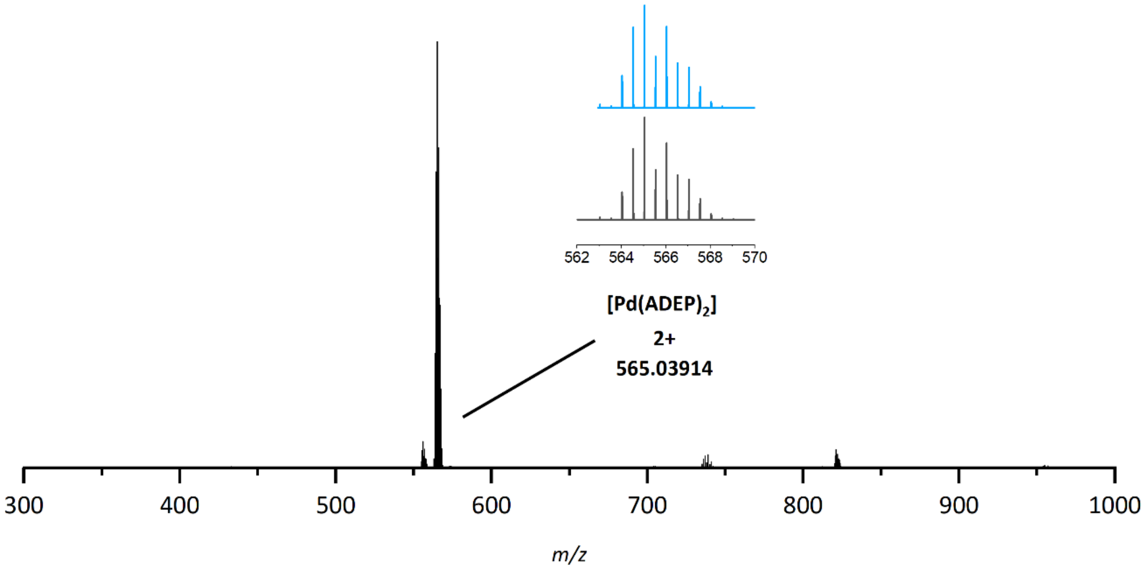

Figure S73. HR-ESI-MS(+) spectrum of **[Pd(ADEP)$_2$](BF$_4$)$_2$.** Blue trace: calculated; black trace: found.

2.4.4. [Pd(DAER)$_2$](BF$_4$)$_2$

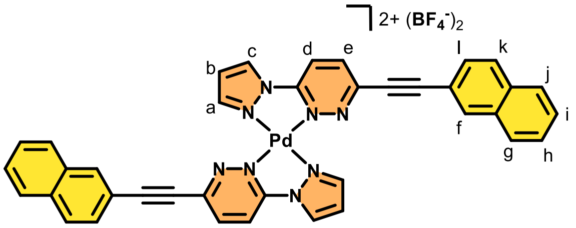

Stock solutions of **DAER** and Pd(MeCN)$_4$(BF$_4$)$_2$ in [D_6]DMSO were combined in 2:1 ratio to 1.88 mM (600 uL) and the mixture analysed by ^{1}H NMR spectroscopy. For **[Pd(DAER)$_2$](BF$_4$)$_2$**:^{1}H NMR (400 MHz, [D_6]DMSO, 298 K) δ: 9.50 (d, J = 3.2 Hz, 1H, H_c), 9.09 (d, J = 9.3 Hz, 1H, H_d), 8.96 (d, J = 9.3 Hz, 1H, H_e), 8.88 (d, J = 2.3 Hz, 1H, H_a), 8.52 (d, J = 1.7 Hz, 1H, H_d), 8.13 (d, J = 8.5 Hz, 1H, H_l), 8.10–8.03 (m, 2H, $H_{g,j}$), 7.87 (dd, J = 8.4, 1.6 Hz, 1H, H_k), 7.73–7.65 (m, 2H, $H_{h,i}$), 7.38 (dd, J = 3.1, 2.3 Hz, 1H, H_b). HR ESI-MS (DMSO/MeCN) m/z = 349.0593 [M–(BF$_4$)$_2$]$^{2+}$ (calcd for $C_{38}H_{24}N_8Pd$, 349.0586); m/z = 785.1194 [M–

$(BF_4)]^+$ (calc. for $BC_{38}H_{24}N_8F_4Pd$, 785.1207). *D* (× 10^{-10} m^2 s^{-1}, 400 MHz, [D$_6$]DMSO, 298 K, [Pd(II)] = 1.88 mM) = 1.22.

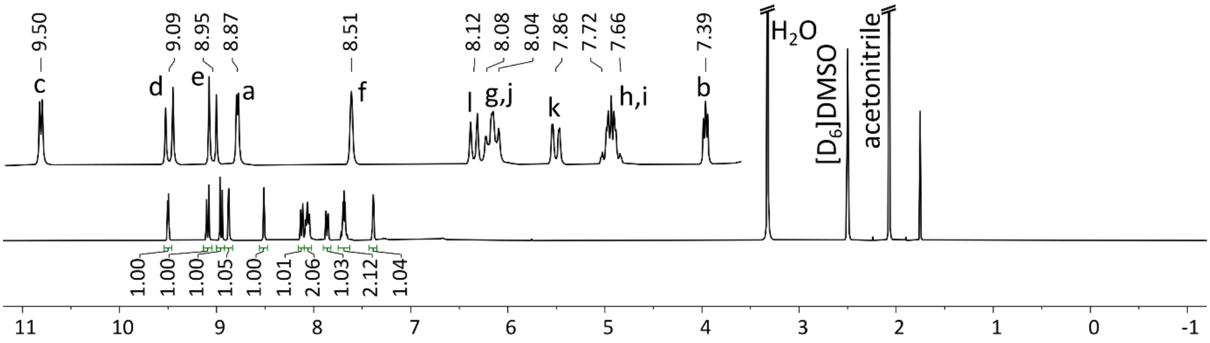

Figure S74. ^{1}H NMR ([D$_6$]DMSO, 400 MHz, 298 K) spectrum of **[Pd(DAER)$_2$](BF$_4$)$_2$.**

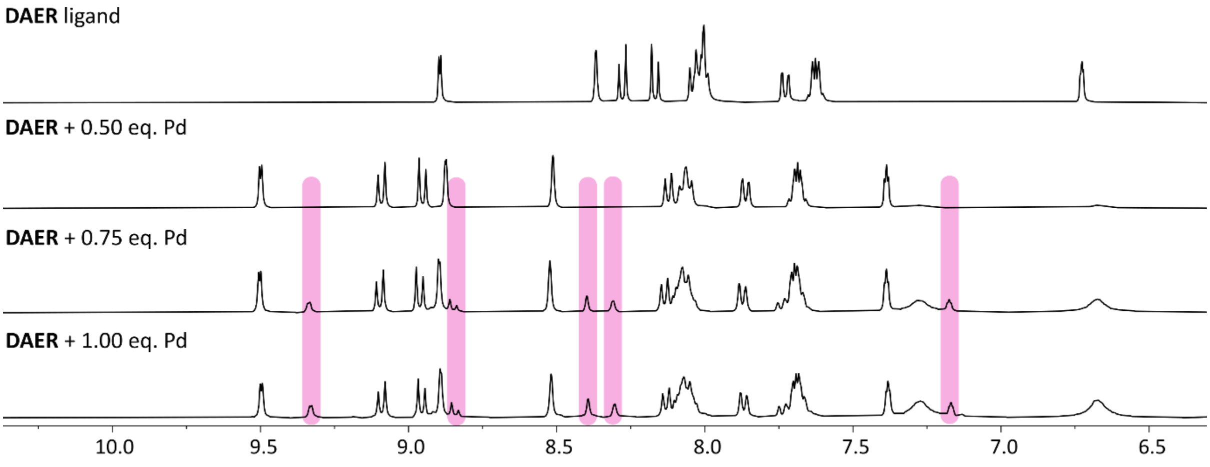

Figure S75. Partial stacked ^{1}H NMR ([D$_6$]DMSO, 400 MHz, 298 K) spectra of **DAER** with increasing equiv. of Pd(II). Pink highlights **[Pd(DAER)(DMSO)$_2$](BF$_4$)$_2$.**

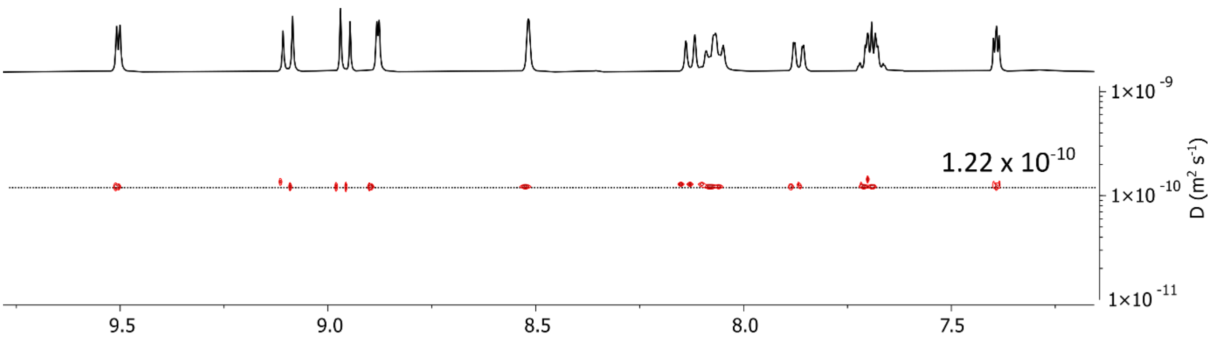

Figure S76. Partial DOSY NMR ([D$_6$]DMSO, 400 MHz, 298 K) spectrum of **[Pd(DAER)$_2$](BF$_4$)$_2$.**

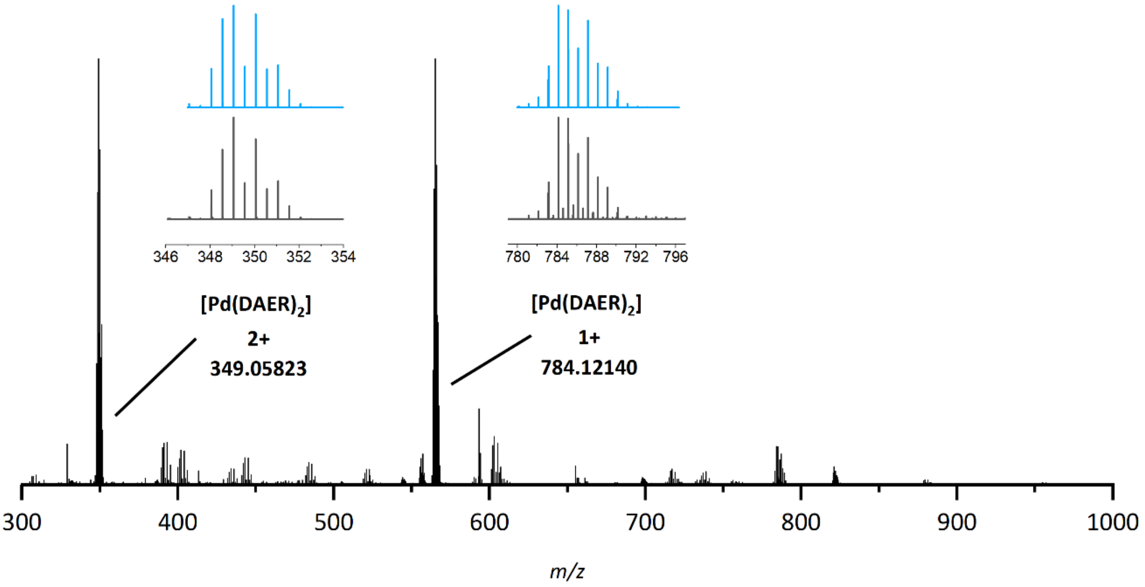

Figure S77. HR-ESI-MS(+) spectrum of **$[Pd(DAER)_2](BF_4)_2$.** Blue trace: calculated; black trace: found.

2.4.5. $[Pd(DDER)_2](BF_4)_2$

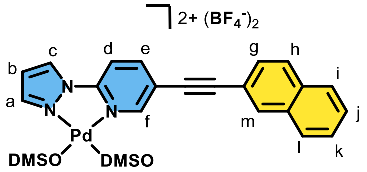

Stock solutions of **DDER** and $Pd(MeCN)_4(BF_4)_2$ in [D$_6$]DMSO were combined in 2:1 ratio to 1.88 mM (600 uL) and the mixture analysed by ^{1}H NMR spectroscopy. For **$[Pd(DDER)_2](BF_4)_2$**: ^{1}H NMR (400 MHz, [D$_6$]DMSO, 298 K) δ: 9.29 (d, J = 3.2 Hz, 1H, H$_c$), 8.77 (dd, J = 8.7, 1.9 Hz, 1H, H$_f$), 8.41 (d, J = 8.8 Hz, 1H, H$_e$), 8.33–8.28 (m, 2H, H$_{a,g}$), 8.21 (d, J = 0.4 Hz, 1H, Hd), 8.06–7.97 (m, 3H, H$_{h,k,l}$), 7.70 (dd, J = 8.4, 1.6 Hz, 1H, H$_m$), 7.66–7.60 (m, 2H, H$_{i,j}$), 7.13 (t, J = 2.8 Hz, 1H, H$_b$). The complex was not observed by HR-ESI-MS. D ($\times 10^{-10}$ m^2 s^{-1}, 400 MHz, [D$_6$]DMSO, 298 K, [Pd(II)] = 1.88 mM) = 1.69.

At 0.50–0.75 eq. Pd added, the equilibrium is between three NMR-active species: **DDER**, **$[Pd(DDER)(DMSO)_2]^{2+}$** and **$[Pd(DDER)_2]^{2+}$**.

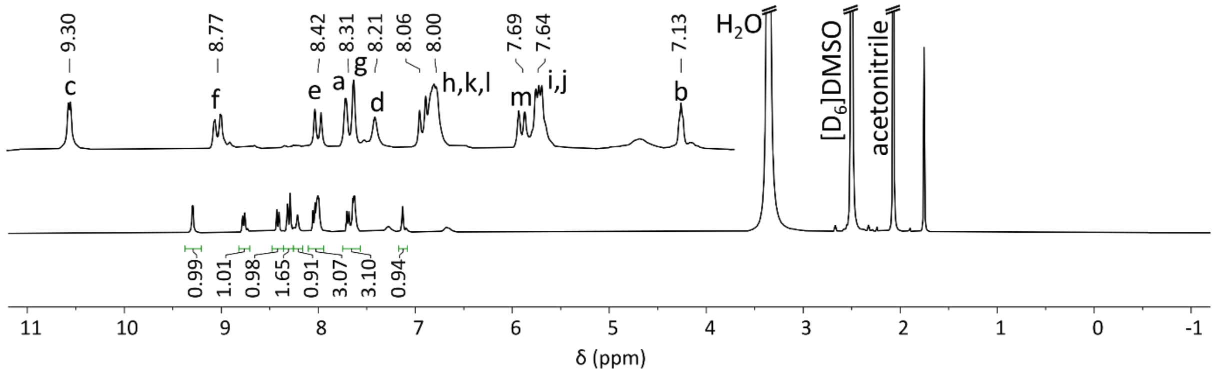

Figure S78. ^{1}H NMR ([D6]DMSO, 400 MHz, 298 K) spectrum of **[Pd(DDER)2](BF4)2.**

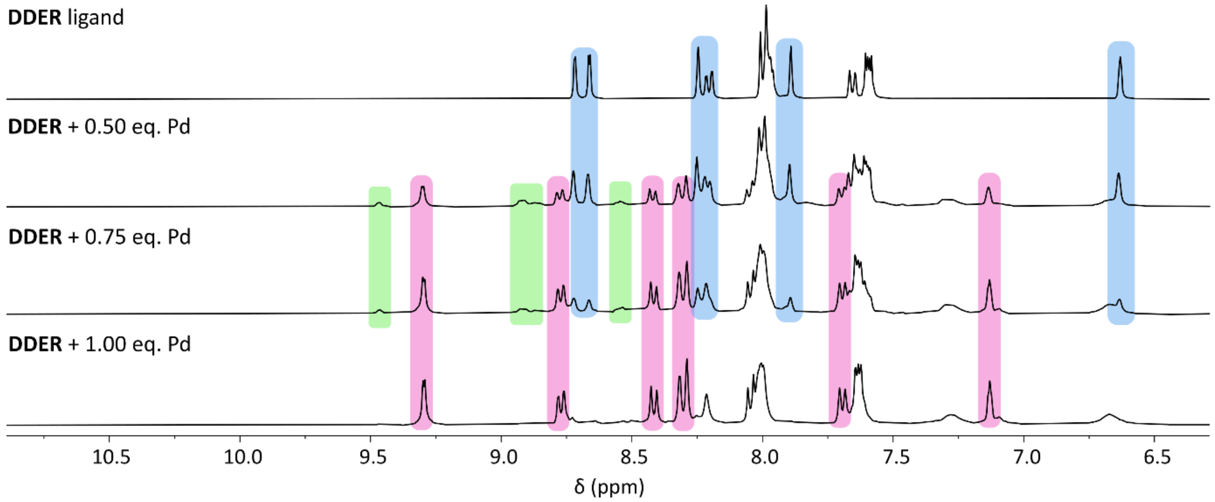

Figure S79. Partial stacked ^{1}H NMR ([D6]DMSO, 400 MHz, 298 K) spectra of **DDER** with increasing equiv. of Pd(II). Blue highlights the free ligand, pink highlights **[Pd(DDER)(DMSO)2](BF4)2** and green highlights **[Pd(DDER)2](BF4)2.**

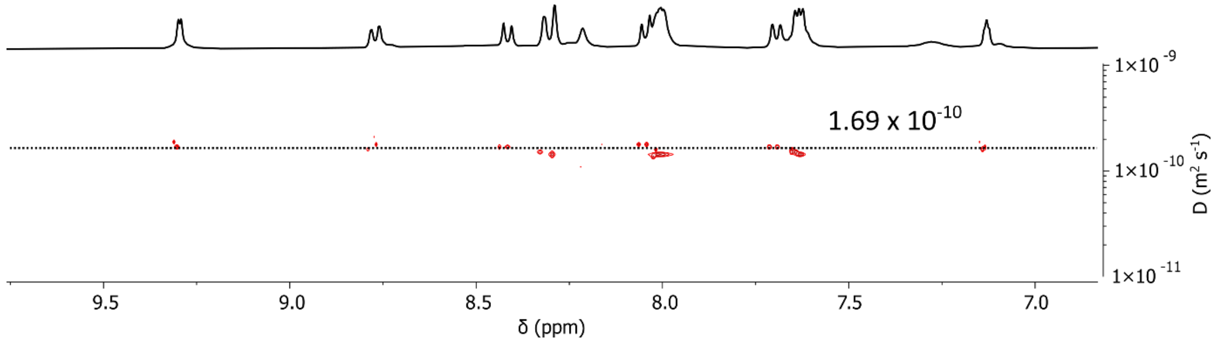

Figure S80. Partial DOSY NMR ([D6]DMSO, 400 MHz, 298 K) spectrum of **[Pd(DDER)(DMSO)2](BF4)2.**

2.4.6. [Pd(DDEP)$_2$](BF$_4$)$_2$

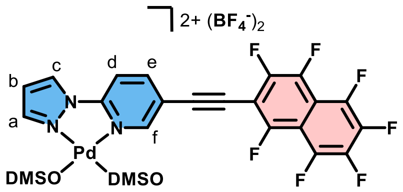

Stock solutions of **DDEP** and Pd(MeCN)$_4$(BF$_4$)$_2$ in [D$_6$]DMSO were combined in 2:1 ratio to 1.88 mM (600 uL) and the mixture analysed by ^{1}H NMR spectroscopy. Further aliquots of Pd(II) were added to increase the ligand:metal ratio to 1:1. For **[Pd(DDEP)$_2$](BF$_4$)$_2$**: ^{1}H NMR (400 MHz, [D$_6$]DMSO, 298 K) δ: 9.34 (d, J = 3.3 Hz, 1H, H$_c$), 8.87 (dd, J = 8.8, 2.0 Hz, 1H, H$_f$), 8.46 (d, J = 8.8 Hz, 1H, H$_e$), 8.32 (d, J = 2.4 Hz, 1H, H$_a$), 8.26 (s, 1H, H$_d$), 7.15 (t, J = 2.6 Hz, 1H, H$_b$). ^{19}F NMR (376 MHz, [D$_6$]DMSO, 298 K) δ: -112.1 (dd, J = 65.9, 17.5 Hz), -134.3 (d, J = 19.4 Hz), -143.8 (dt, J = 64.7, 17.2 Hz), -146.5 (dt, J = 56.6, 17.5 Hz), -148.3 (s), -148.9 (dt, J = 57.1, 18.4 Hz), -150.9 (t, J = 20.2 Hz), -154.5 (t, J = 21.3 Hz). The complex was not observed by HR-ESI-MS. D (× 10^{-10} m^2 s^{-1}, 400 MHz, [D$_6$]DMSO, 298 K, [Pd(II)] = 1.88 mM) = 1.37.

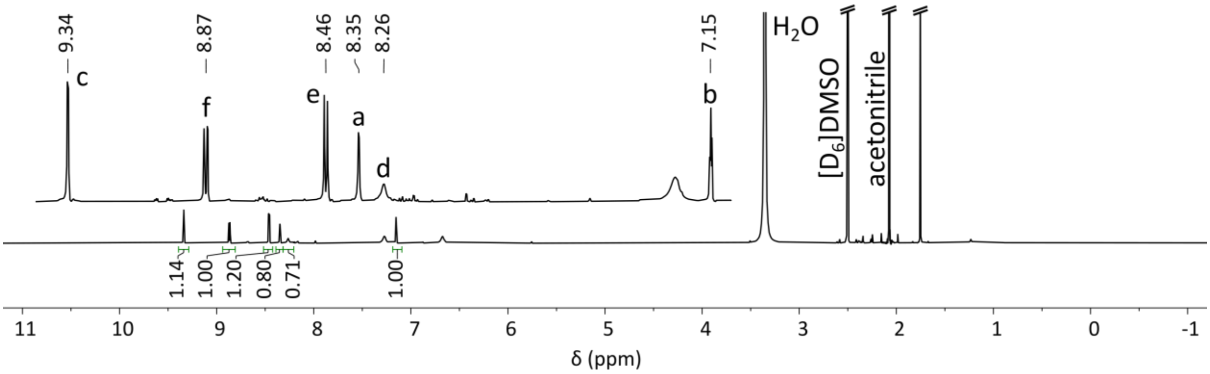

Figure S81. ^{1}H NMR ([D$_6$]DMSO, 400 MHz, 298 K) spectrum of **[Pd(DDEP)(DMSO)$_2$](BF$_4$)$_2$.**

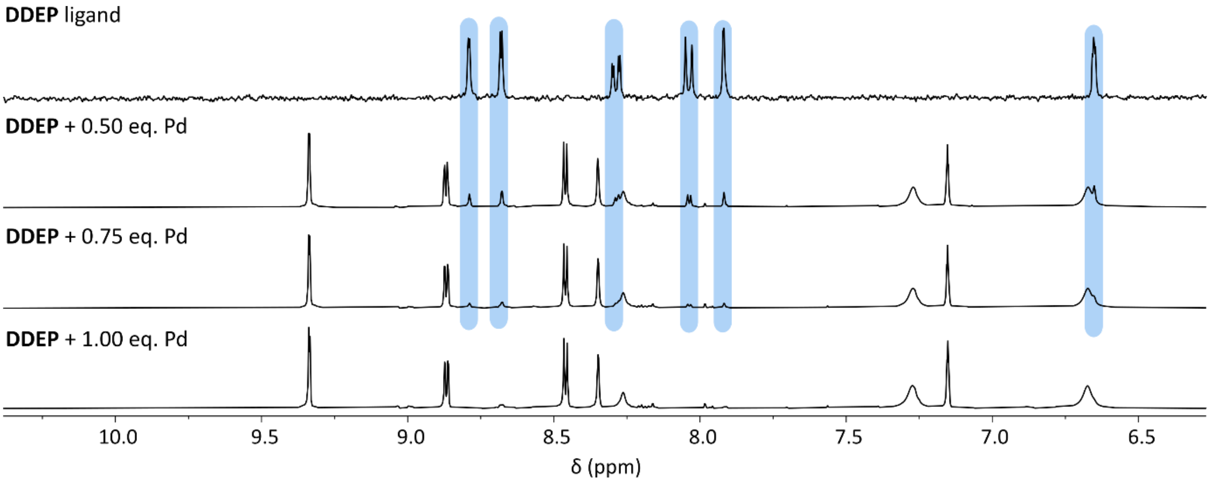

Figure S82. Partial stacked ^{1}H NMR ([D$_6$]DMSO, 400 MHz, 298 K) spectra of **DDEP** with increasing equiv. of Pd(II). Blue highlights the free ligand.

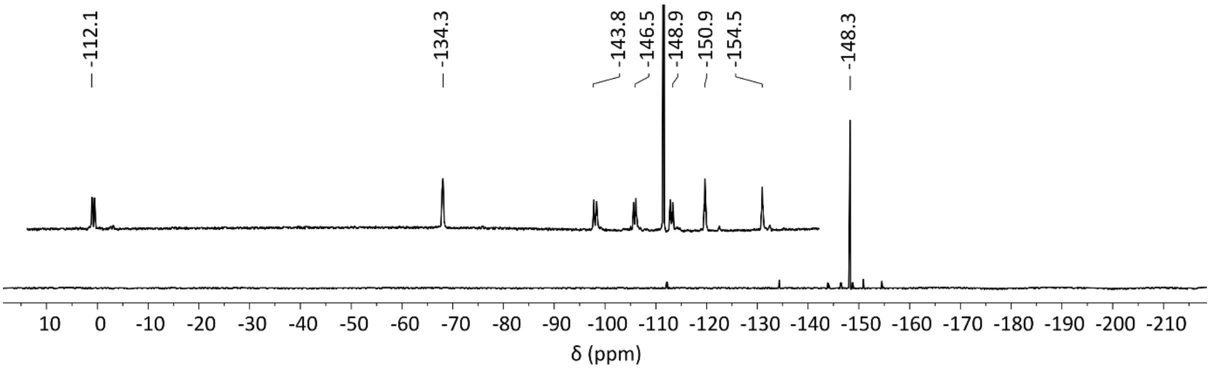

Figure S83. ^{19}F NMR ([D$_6$]DMSO, 376 MHz, 298 K) spectrum of **[Pd(DDEP)(DMSO)$_2$](BF$_4$)$_2$.**

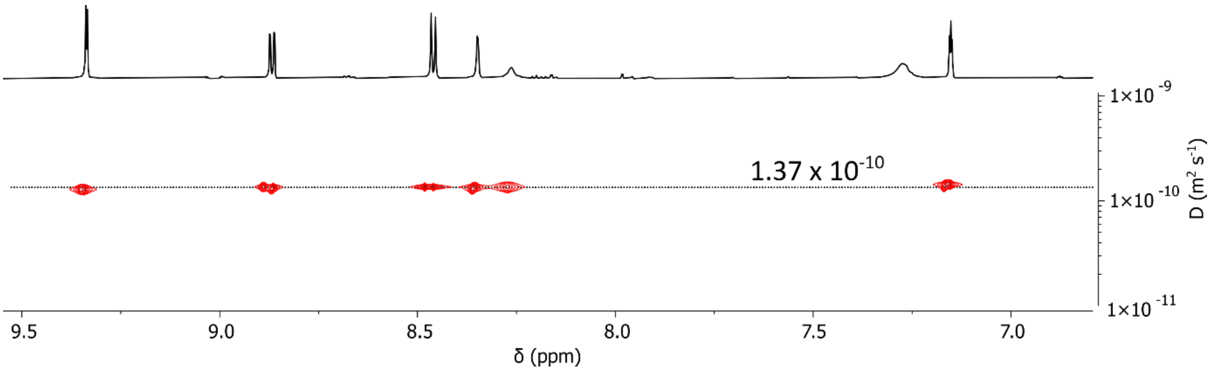

Figure S84. Partial DOSY NMR ([D$_6$]DMSO, 400 MHz, 298 K) spectrum of **[Pd(DDEP)(DMSO)$_2$](BF$_4$)$_2$.**

2.5. Heteroleptic Complexes

2.5.1. Combining [Pd(ADEP)$_2$](BF$_4$)$_2$ and [Pd(DAER)$_2$](BF$_4$)$_2$

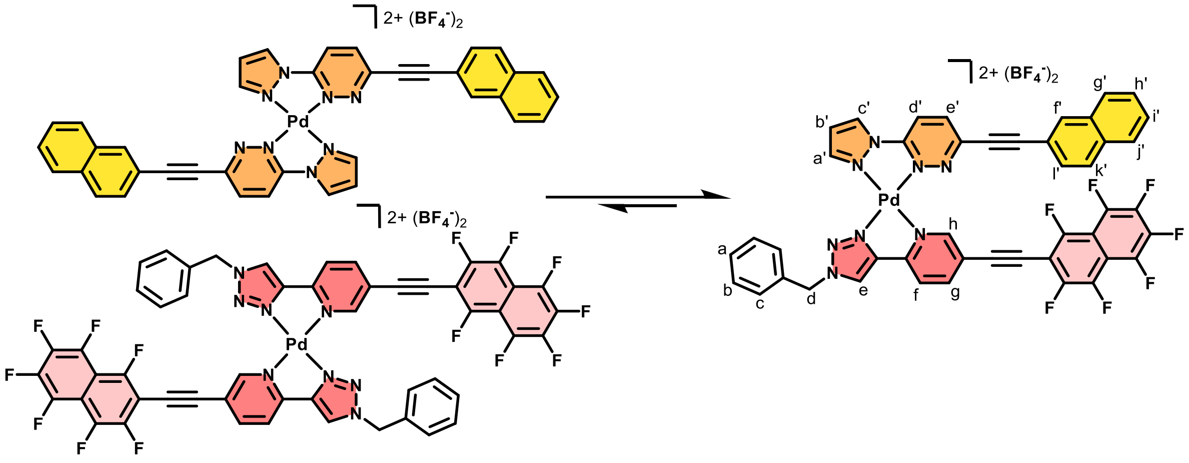

Stock solutions (7.5 mM in [D$_6$]DMSO) of **[Pd(ADEP)$_2$]$^{2+}$** and **[Pd(DAER)$_2$]$^{2+}$** were combined in 1:1 ratio to 1.88 mM (600 uL) and the mixture analysed by ^{1}H NMR spectroscopy and ESI-MS spectrometry. For **Pd[(ADEP)(DAER)](BF$_4$)$_2$**: ^{1}H NMR (400 MHz, [D$_6$]DMSO, 298 K) δ: 9.97 (dd, J = 1.9 Hz, 1H, H$_h$), 9.56–9.52 (m, 2H, H$_{e,c'}$), 9.11 (d, J = 9.2 Hz, 1H, H$_{d'}$), 8.99 (d, J = 9.2 Hz, 1H, H$_{e'}$), 8.90 (d, J = 2.4 Hz, 1H, H$_{a'}$), 8.86 (dd, J = 8.2, 1.8 Hz, 1H, H$_g$), 8.59 (d, J = 8.2 Hz, 1H, H$_f$), 8.08 (s, 1H, H$_{f'}$), 7.67–7.59 (m, 5H, H$_{c,g',j',l'}$), 7.56–7.36 (m, 7H, H$_{a,b,b',h',i',k'}$), 6.08 (s, 2H, H$_d$). ^{19}F NMR (376 MHz, [D$_6$]DMSO, 298 K) δ: -112.0 (dd, J = 65.7, 16.5 Hz), –134.1 (d, J = 19.0 Hz), –143.7 (dt, J = 65.4, 16.7 Hz), –146.3 (dt, J = 56.8, 22.5 Hz), –148.2 (s), –148.8 (d, J = 2.2 Hz), –151.0 (t, J = 19.0 Hz), -154.8 (t, J = 17.6 Hz). HR ESI-MS (DMSO) m/z = 349.0567 [Pd(DAER)$_2$]$^{2+}$ (calcd for C$_{38}$H$_{24}$N$_8$Pd, 349.0581); 457.0481 [M]$^{2+}$ (calcd dfor C$_{45}$H$_{23}$F$_7$N$_8$Pd, 457.0487); 565.0385 [Pd(ADEP)$_2$]$^{2+}$ (calcd for C$_{52}$H$_{22}$F$_{14}$N$_8$Pd, 565.0393) 823.0424 [M-C$_7$H$_7$]$^+$ (calcd for C$_{38}$H$_{16}$F$_7$N$_8$Pd, 823.0429); 1001.1002 [M+BF$_4$]$^+$ (calcd for C$_{45}$H$_{23}$F$_7$N$_8$PdBF$_4$, 1001.1003). D ($\times$ 10^{-10} m^2 s^{-1}, 400 MHz, [D$_6$]DMSO, 298 K, [Pd(II)] = 1.88 mM) = 1.22.

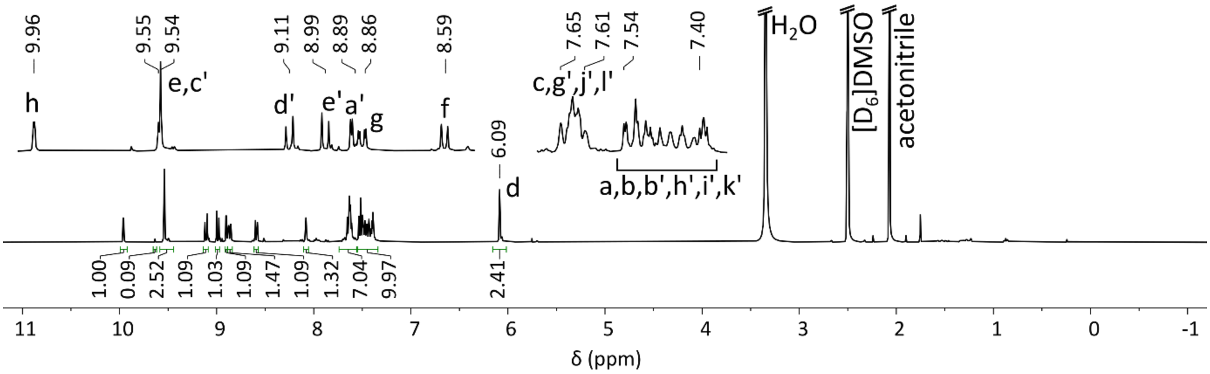

Figure S85. ^{1}H NMR ([D$_6$]DMSO, 400 MHz, 298 K) spectrum of **[Pd(ADEP)(DAER)](BF$_4$)$_2$.**

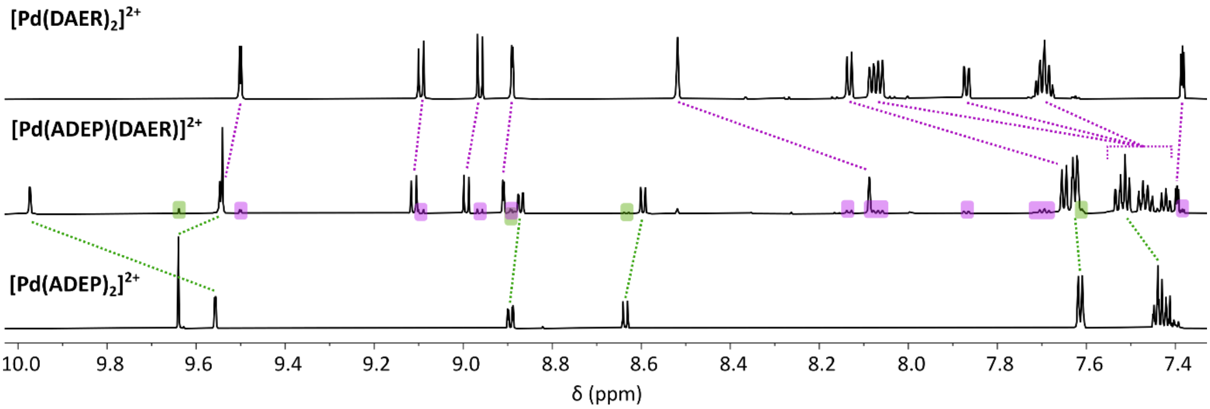

Figure S86. Partial stacked ^{1}H NMR ([D$_6$]DMSO, 400 MHz, 298 K) spectra of **[Pd(ADEP)$_2$]$^{2+}$, [Pd(ADEP)(DAER)]$^{2+}$**, and **Pd(ADEP)$_2$]$^{2+}$.** Pink highlights **[Pd(ADEP)$_2$]$^{2+}$**and green highlights **Pd(ADEP)$_2$]$^{2+}$.**

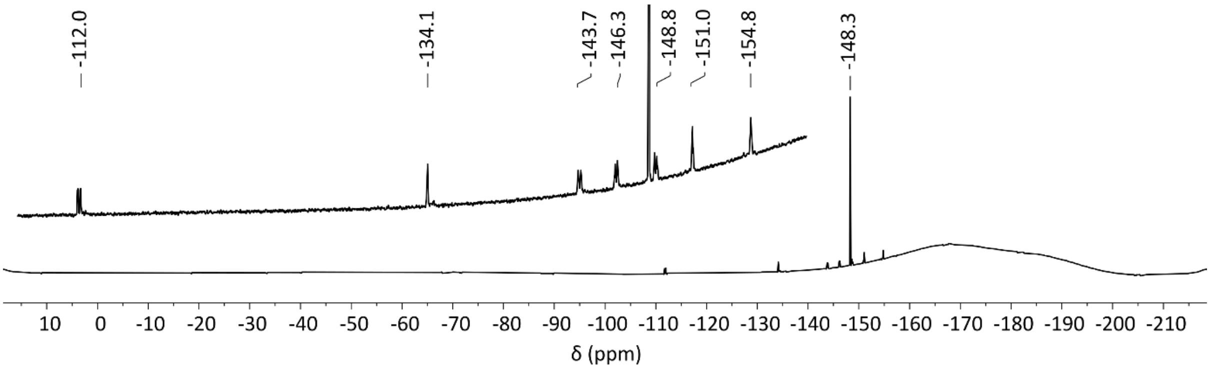

Figure S87. ^{19}F NMR ([D$_6$]DMSO, 376 MHz, 298 K) spectrum of **[Pd(ADEP)(DAER)](BF$_4$)$_2$.**

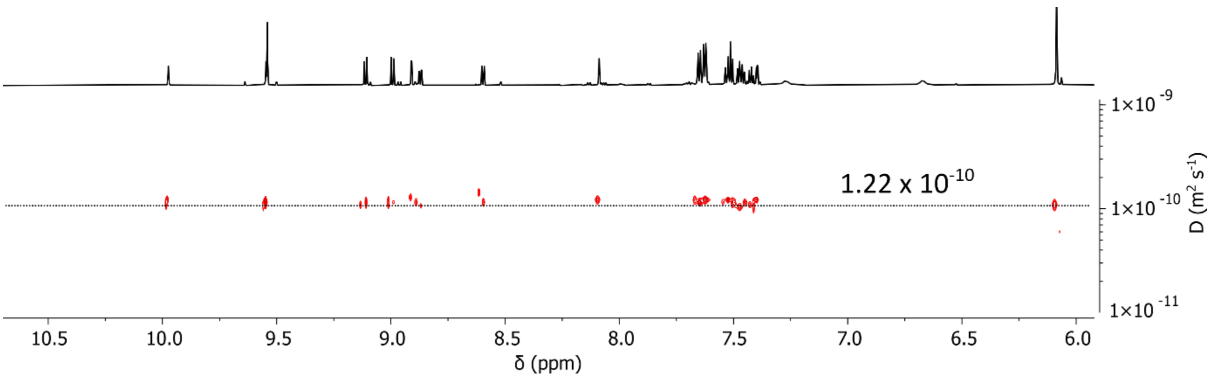

Figure S88. Partial DOSY NMR ([D$_6$]DMSO, 400 MHz, 298 K) spectrum of **[Pd(ADEP)(DAER)](BF$_4$)$_2$.**

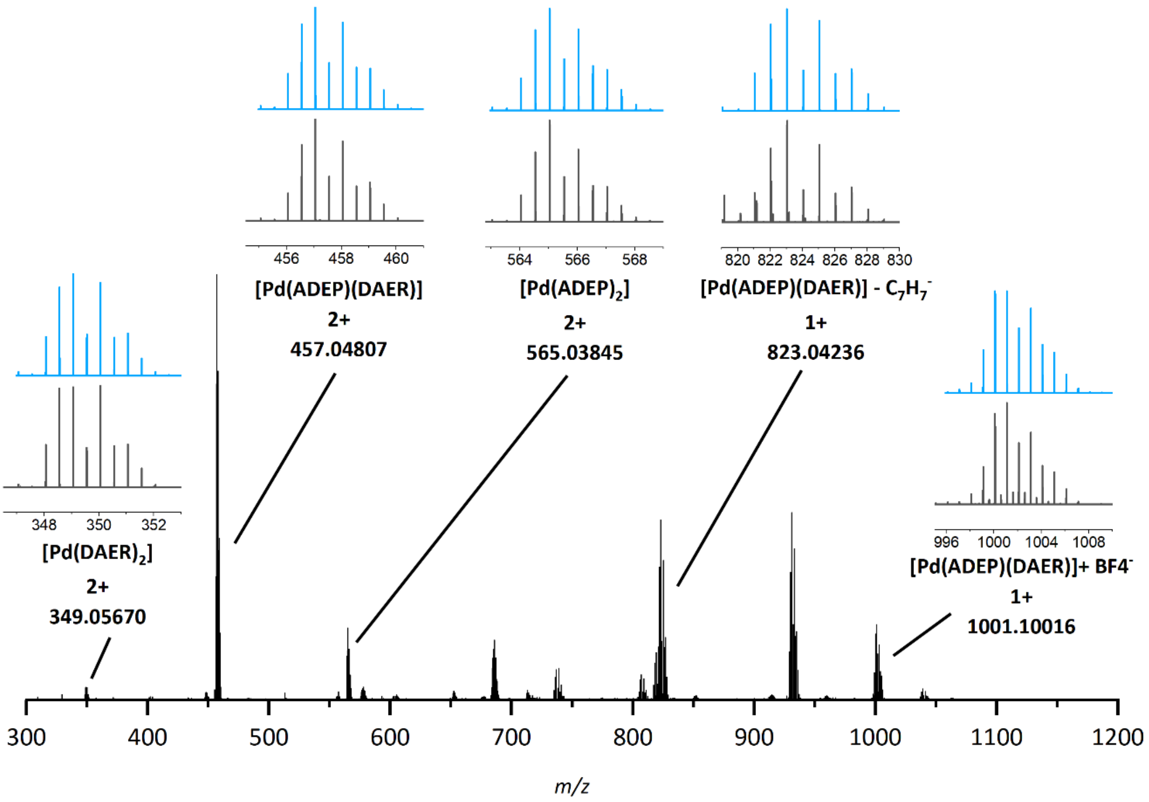

Figure S89. HR Nanospray-MS(+) spectrum of **[Pd(ADEP)(DAER)$_2$](BF$_4$)$_2$.** Blue trace: calculated; black trace: found.

2.5.2. Combining [Pd(AAEP)$_2$](BF$_4$)$_2$ and [Pd(DDER)$_2$](BF$_4$)$_2$

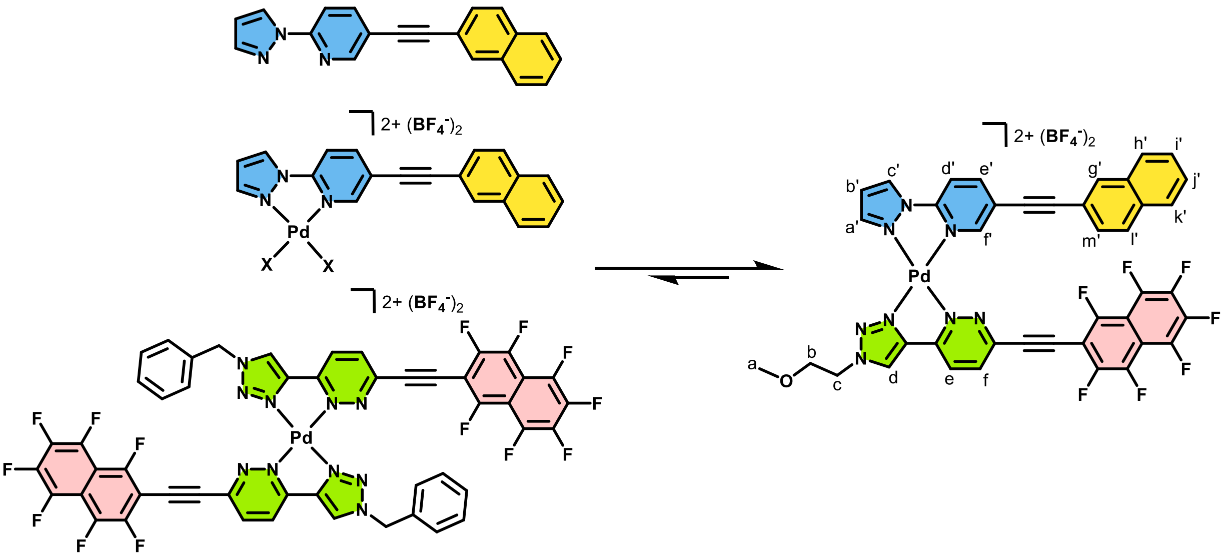

Stock solutions (7.5 mM in [D$_6$]DMSO) of **[Pd(AAEP)$_2$]$^{2+}$** and **[Pd(DDER]$^{2+}$** (at L:M ratio 2:1) were combined in 1:1 ratio to 1.88 mM (600 uL) and the mixture analysed by ^{1}H NMR spectroscopy and ESI-MS spectrometry. For **Pd(AAEP)(DDER)(BF$_4$)$_2$**: ^{1}H NMR (400 MHz, [D$_6$]DMSO, 298 K) δ: 9.74 (d, J = 1.9 Hz, 1H, H$_{d'}$), 9.62 (s, 1H, H$_d$), 9.49 (d, J = 3.2 Hz, 1H, H$_{c'}$), 9.14 (d, J = 8.7 Hz, 1H, H$_e$), 9.01 (d, J = 8.7 Hz,

1H, H_f), 8.88 (dd, J = 8.7, 2.0 Hz, 1H, $H_{f'}$), 8.77 (d, J = 2.4 Hz, 1H, $H_{a'}$), 8.59 (d, J = 8.7 Hz, 1H, $H_{e'}$), 7.86 (s, 1H, $H_{g'}$), 7.68 (d, J = 8.1 Hz, 1H, $H_{l'}$), 7.63–7.56 (m, 2H, $H_{h',k'}$), 7.51–7.37 (m, 3H, $H_{i',j',m'}$), 7.29 (t, J = 2.7 Hz, 1H, $H_{b'}$), 5.08 (t, J = 4.8 Hz, 2H, H_c), 3.98 (d, J = 4.8 Hz, 1H, H_b). ^{19}F NMR (376 MHz, $CDCl_3$, 298 K) δ: –110.5 (dd, J = 65.8, 16.6 Hz), –133.9 (d, J = 18.8 Hz), –143.3 (dd, J = 66.0, 17.5 Hz), –145.9 (dd, J = 57.4, 17.2 Hz), –148.3 (s), –148.4 (t, J = 19.0 Hz), –150.0 (t, J = 18.1 Hz), –154.4 (dd, J = 19.4 Hz). HR ESI-MS (DMSO) m/z = 441.0477 $[M]^{2+}$ (calcd for $C_{41}H_{23}F_7N_8OPd$, 441.0461); 534.0306 $[Pd(AAEP)_2]^{2+}$ (calcd for $C_{42}H_{20}F_{14}N_{10}O_2Pd$, 534.0293); 823.0457 $[M\text{-}C_7H_7]^+$ (calcd for $C_{38}H_{16}F_7N_8Pd$, 823.0429); 969.0996 $[M+BF_4]^+$ (calcd for $C_{41}H_{23}F_7N_8OPdBF_4$, 969.0962). D ($\times 10^{-10}$ m^2 s^{-1}, 400 MHz, [D$_6$]DMSO, 298 K, [Pd(II)] = 1.88 mM) = 1.22.

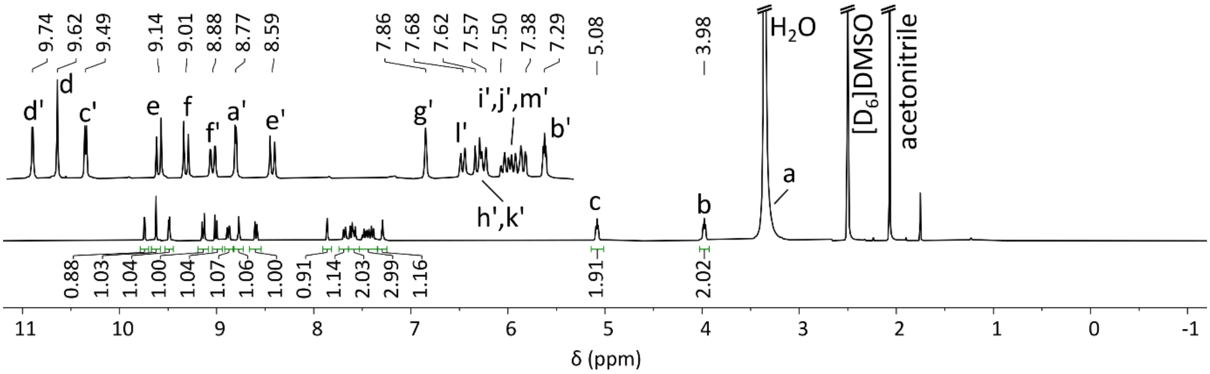

Figure S90. ^{1}H NMR ([D$_6$]DMSO, 400 MHz, 298 K) spectrum of **[Pd(AAEP)(DDER)]$^{2+}$.**

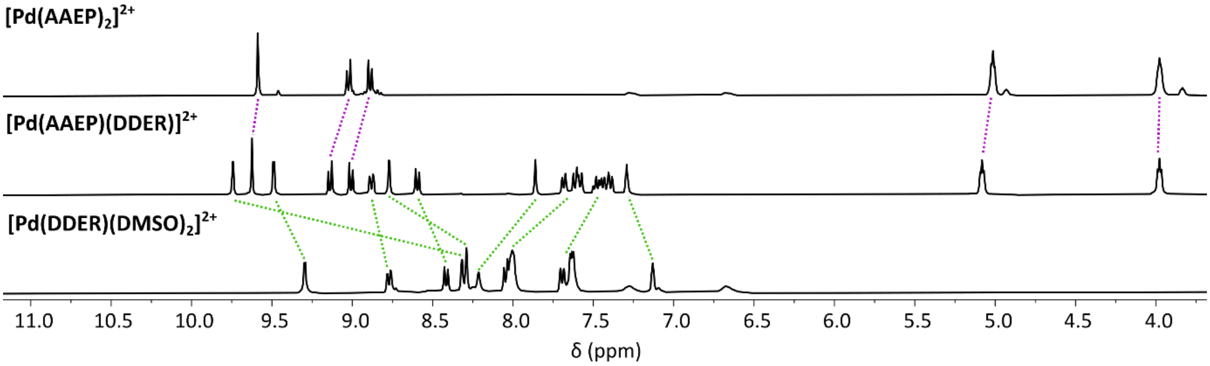

Figure S91. Partial stacked ^{1}H NMR ([D$_6$]DMSO, 400 MHz, 298 K) spectra of **[Pd(AAEP)$_2$]$^{2+}$, [Pd(AAEP)(DDER)]$^{2+}$**, and **Pd(DDER)(DMSO)$_2$]$^{2+}$.**

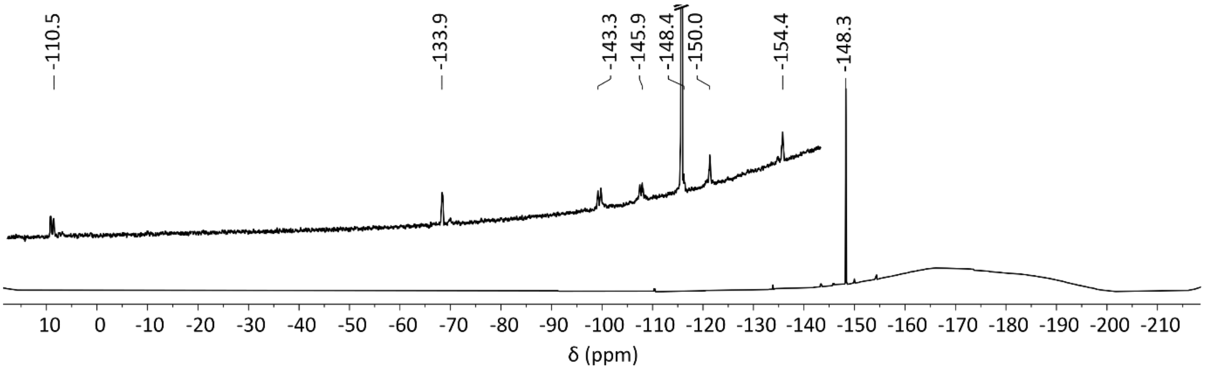

Figure S92. ^{19}F NMR ([D$_6$]DMSO, 376 MHz, 298 K) spectrum of **[Pd(AAEP)(DDER)](BF$_4$)$_2$.**

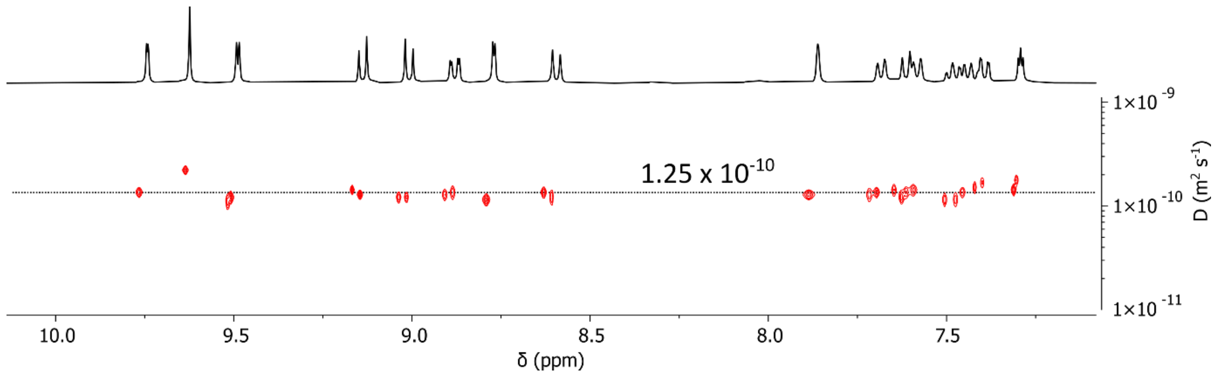

Figure S93. Partial DOSY NMR ([D$_6$]DMSO, 400 MHz, 298 K) spectrum of **[Pd(AAEP)(DDER)]$^{2+}$.**

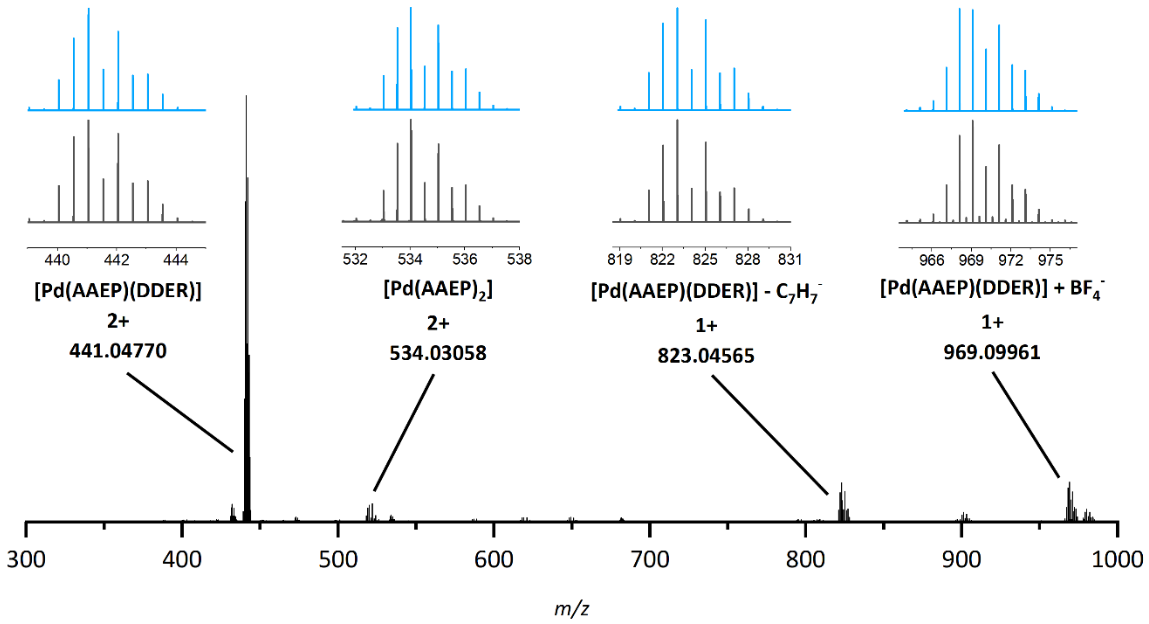

Figure S94. HR Nanospray-MS(+) spectrum of **[Pd(AAEP)(DDER)](BF$_4$)$_2$.** Blue trace: calculated; black trace: found.

2.5.3. Combining $[Pd(AAER)_2](BF_4)_2$ and $[Pd(DDEP)](BF_4)_2$

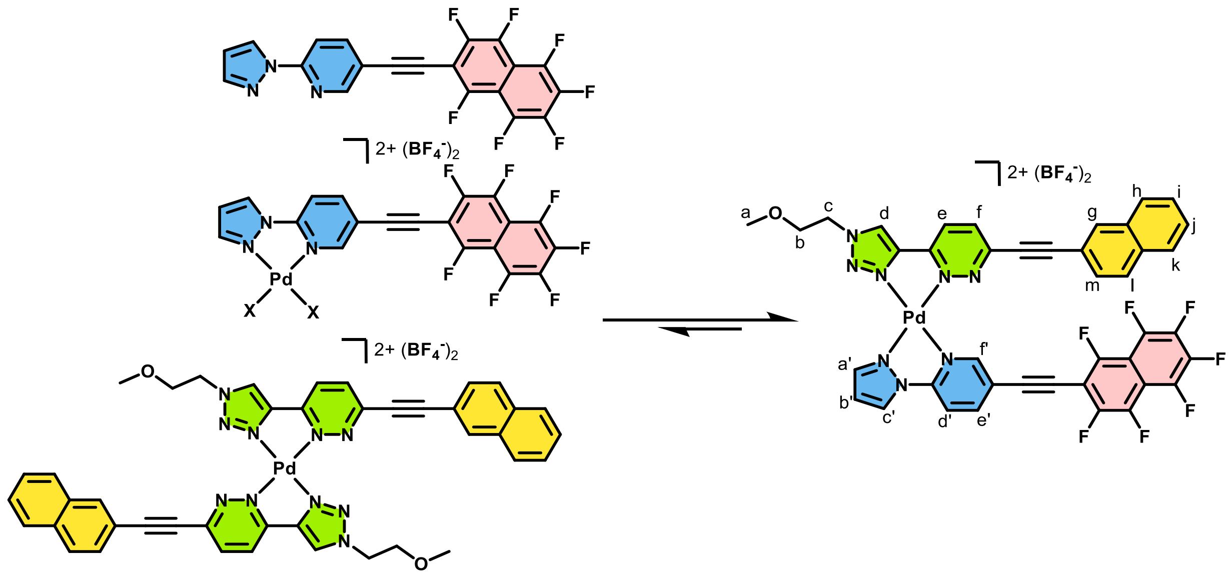

Stock solutions (4.5 mM in [D6]DMSO) of **$[Pd(AAER)_2]^{2+}$** and **$[Pd(DDEP)(DMSO)_2]^{2+}$** (at L:M ratio 2:1) were combined in 1:1 ratio to 1.88 mM (600 uL) and the mixture analysed by ^{1}H NMR spectroscopy and ESI-MS spectrometry. For **$Pd(AAER)(DDEP)(BF_4)_2$**: ^{1}H NMR (400 MHz, [D6]DMSO, 298 K) δ: 9.91 (d, J = 2.0 Hz, 1H, H_{2d}), 9.60 (s, 1H, H_{1d}), 9.53 (d, J = 3.2 Hz, 1H, H_{2c}), 9.06 (d, J = 8.7 Hz, 1H, H_{1e}), 9.01 (dd, J = 8.7, 2.0 Hz, 1H, H_{2f}), 8.87 (d, J = 8.8 Hz, 1H, H_{1f}), 8.80 (d, J = 2.4 Hz, 1H, H_{2a}), 8.66 (d, J = 8.7 Hz, 1H, H_{2e}), 8.11 (d, J = 1.5 Hz, 1H, H_{1g}), 7.67–7.60 (m, 3H, $H_{1h,1k,1l}$), 7.54 (dd, J = 8.4, 1.7 Hz, 1H, H_m), 7.48–7.39 (m, 2H, $H_{1i,1j}$), 7.32 (t, J = 2.8 Hz, 1H, H_{2b}), 5.07 (t, J = 4.7 Hz, 2H, H_{1c}), 3.98 (t, J = 5.0 Hz, 2H, H_{1b}), 3.36 (s, 3H, H_{1a}). ^{19}F NMR (376 MHz, $CDCl_3$, 298 K) δ: -111.9 (dd, J = 66.4, 17.0 Hz), -134.1 (d, J = 18.7 Hz), –134.9 (dt, J = 67.1, 17.0 Hz), -146.2 (dt, J = 74.4, 16.9 Hz), -148.3 (s), -148.7 (dt, J = 57.2, 18.6 Hz), -151.2 (t, J = 20.0 Hz), -154.8 (t, J = 18.5 Hz). HR ESI-MS (DMSO) m/z = 441.0477 $[M]^{2+}$ (calcd for $C_{41}H_{23}F_7N_8OPd$, 441.0461); 823.0455 $[M-C_7H_7]^+$ (calcd for $C_{38}H_{26}F_7N_8Pd$, 823.0429); 969.0991 $[M+BF_4]^+$ (calcd for $C_{41}H_{23}F_7N_8OPdBF_4$, 969.0962). D ($\times$ 10^{-10} m^2 s^{-1}, 400 MHz, [D6]DMSO, 298 K, [Pd(II)] = 1.88 mM) = 1.22.

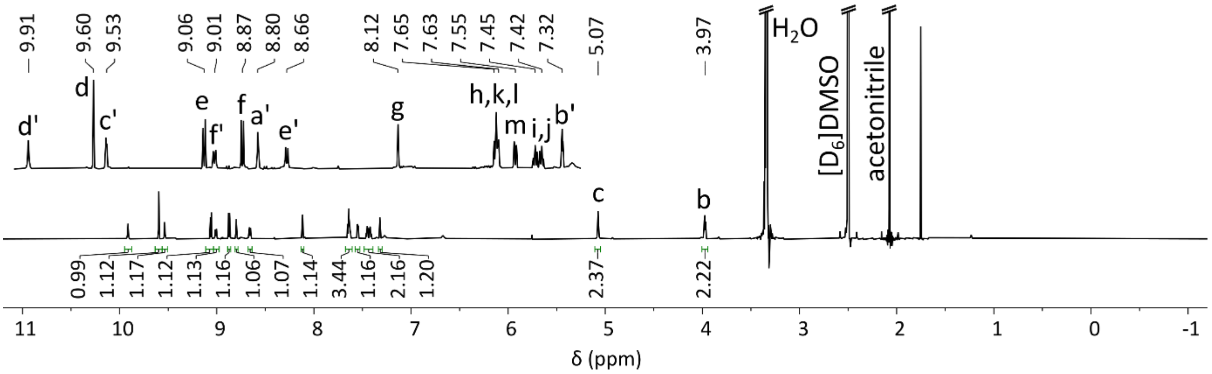

Figure S95. ^{1}H NMR ([D6]DMSO, 400 MHz, 298 K) spectrum of **$[Pd(AAER)(DDER)]^{2+}$**.

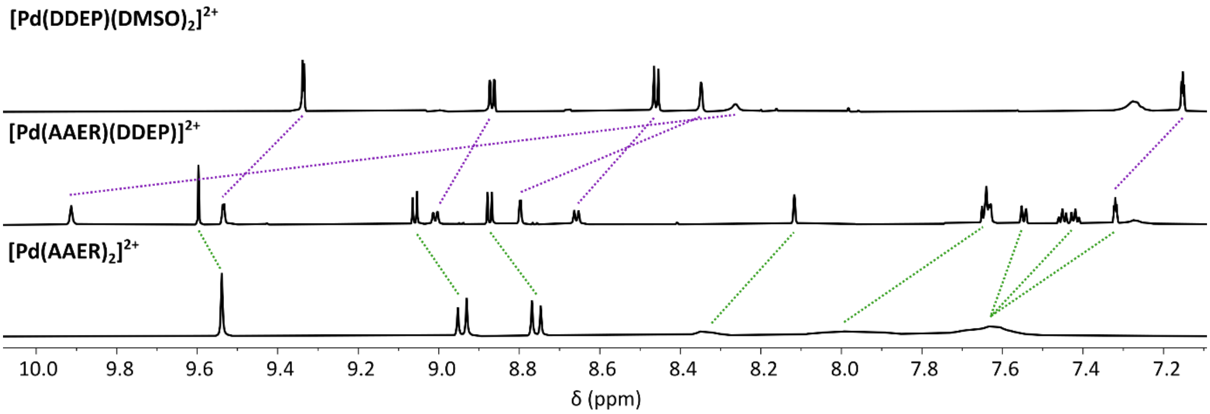

Figure S96. Partial stacked ^{1}H NMR ([D$_6$]DMSO, 400 MHz, 298 K) spectra of **[Pd(DDEP)(DMSO)$_2$]$^{2+}$, [Pd(AAER)(DDEP)]$^{2+}$,** and **Pd(AAER)$_2$]$^{2+}$.**

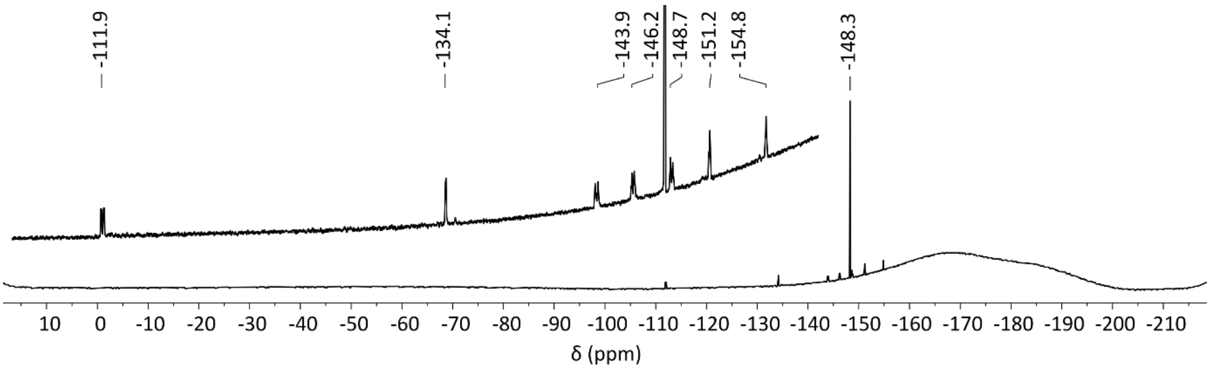

Figure S97. ^{19}F NMR ([D$_6$]DMSO, 376 MHz, 298 K) spectrum of **[Pd(AAER)(DDER)](BF$_4$)$_2$.**

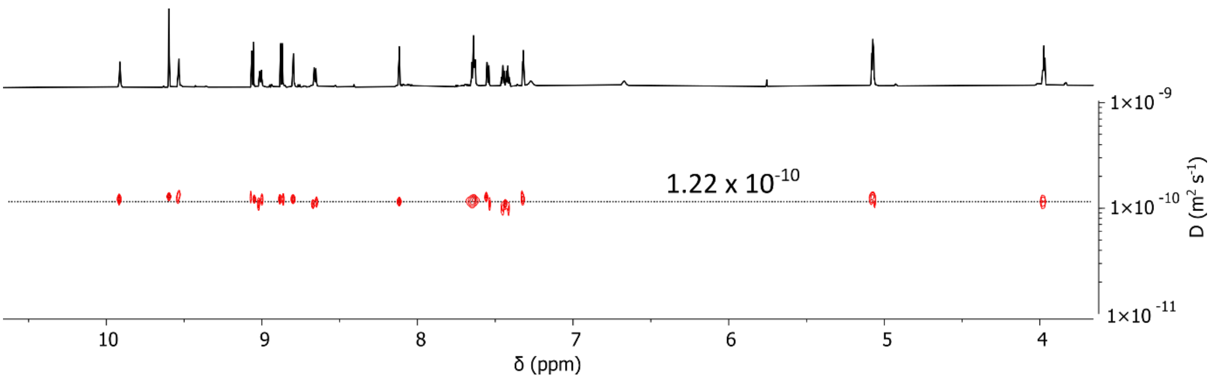

Figure S98. Partial DOSY NMR ([D$_6$]DMSO, 400 MHz, 298 K) spectrum of **[Pd(AAER)(DDEP)](BF$_4$)$_2$.**

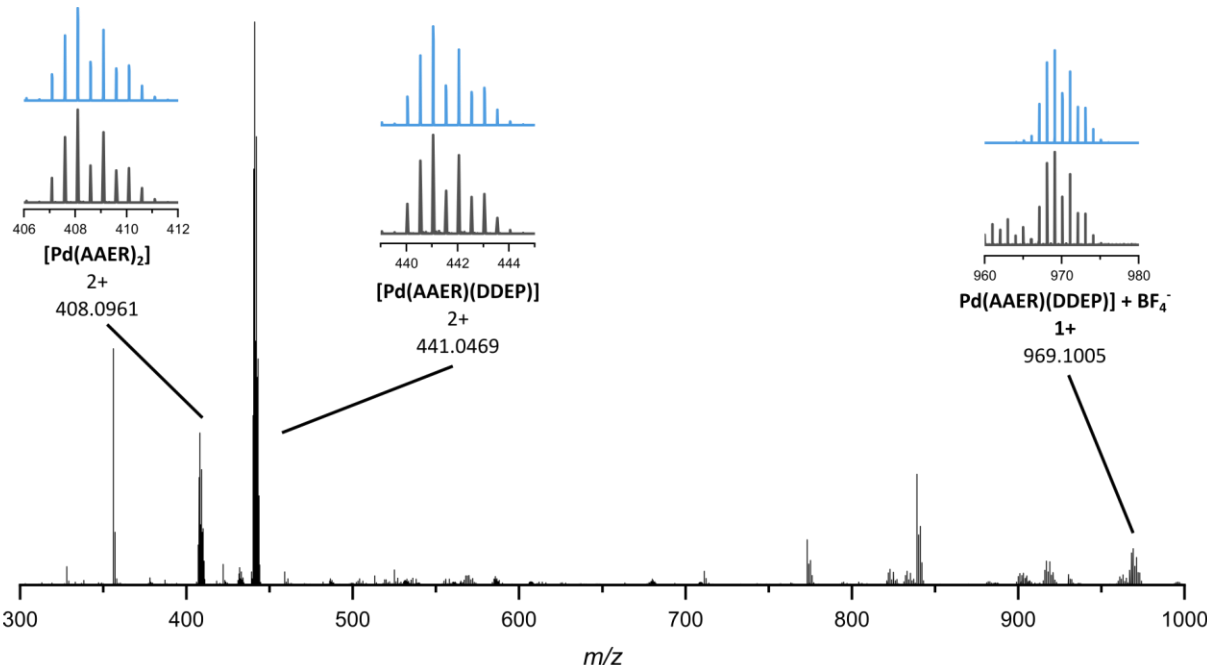

Figure S99. HR-ESI-MS(+) spectrum of **[Pd(AAER)(DDEP)](BF$_4$)$_2$.** Blue trace: calculated; black trace: found.

2.5.4. Combining [Pd(DAER)$_2$](BF$_4$)$_2$ and [Pd(ADER)$_2$](BF$_4$)$_2$

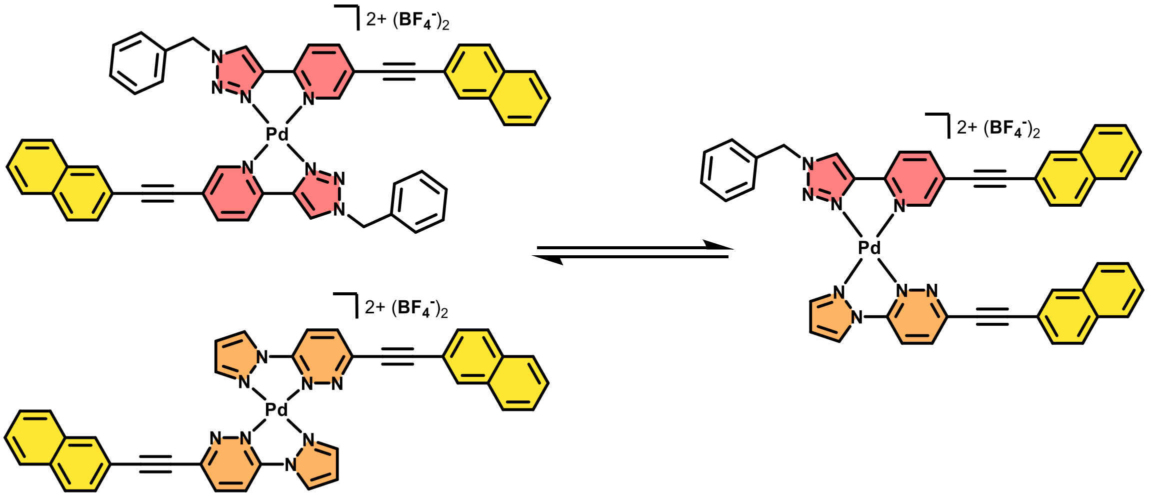

Stock solutions (7.5 mM in [D$_6$]DMSO) of **[Pd(ADER)$_2$]$^{2+}$** and **[Pd(DAER]$^{2+}$** were combined in 1:1 ratio to 1.88 mM and the mixture analysed by ^{1}H NMR spectroscopy and ESI-MS spectrometry. A reliable diffusion coefficient could not be determined from the DOSY NMR data. HR ESI-MS (DMSO/MeCN) *m/z* = 349.0523 [Pd(DAER)$_2$]$^{2+}$ (calcd for $C_{38}H_{24}N_8Pd$, 349.0592); 394.0751 [Pd(DAER)(ADER)]$^{2+}$ (calcd for $C_{45}H_{32}N_8Pd$, 394.0809); 439.0970 [Pd(ADER)$_2$]$^{2+}$ (calcd for $C_{52}H_{36}N_8Pd$, 439.1058).

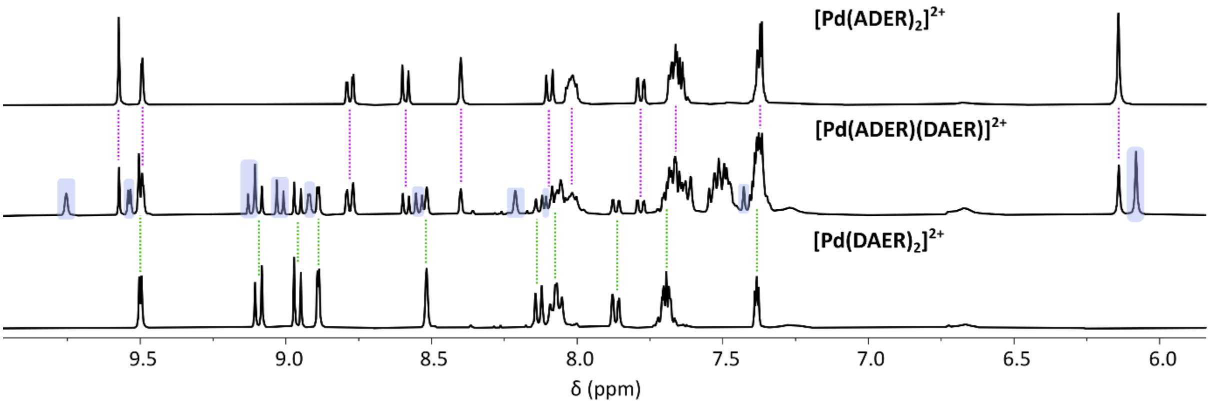

Figure S100. Partial stacked ^{1}H NMR ([D$_6$]DMSO, 400 MHz, 298 K) spectra of **$[Pd(ADER)_2]^{2+}$, $[Pd(ADER)(DAER)]^{2+}$,** and **$Pd(DAER)_2]^{2+}$**. Blue highlights the new species **$[Pd(ADER)(DAER)]^{2+}$.**

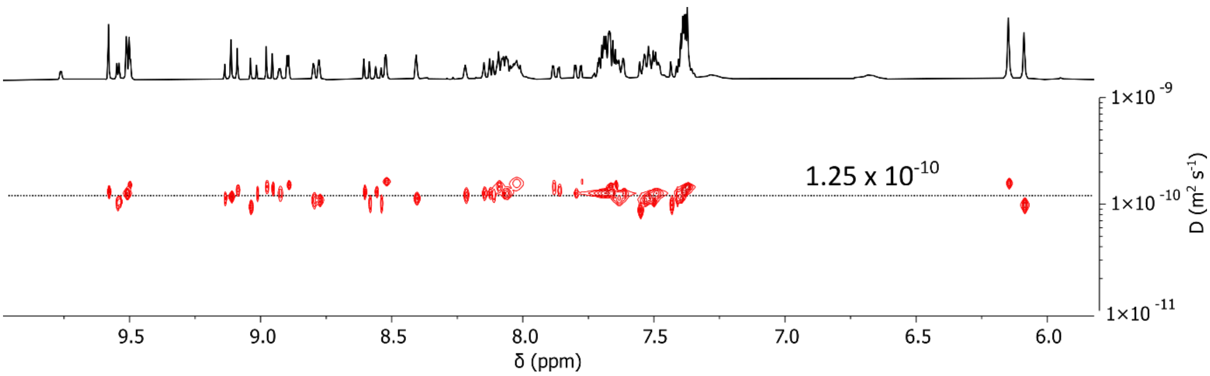

Figure S101. Partial DOSY NMR ([D$_6$]DMSO, 400 MHz, 298 K) spectrum of the mixture of **$[Pd(ADER)_2]^{2+}$, $[Pd(ADER)(DAER)]^{2+}$,** and **$Pd(DAER)_2]^{2+}$.**

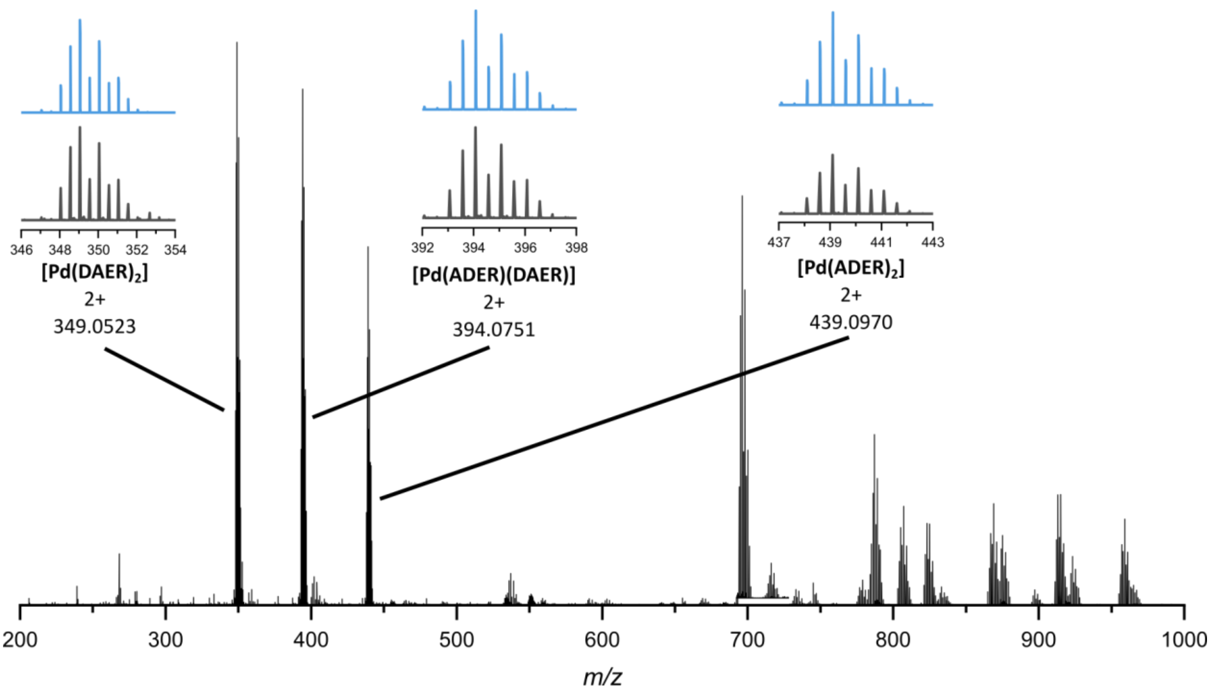

Figure S102. HR Nanospray-MS(+) spectrum of **$[Pd(ADER)(DAER)](BF_4)_2$.** Blue trace: calculated; black trace: found.

2.5.5. Combining [Pd(AAER)$_2$](BF$_4$)$_2$ and [Pd(AAEP)$_2$](BF$_4$)$_2$

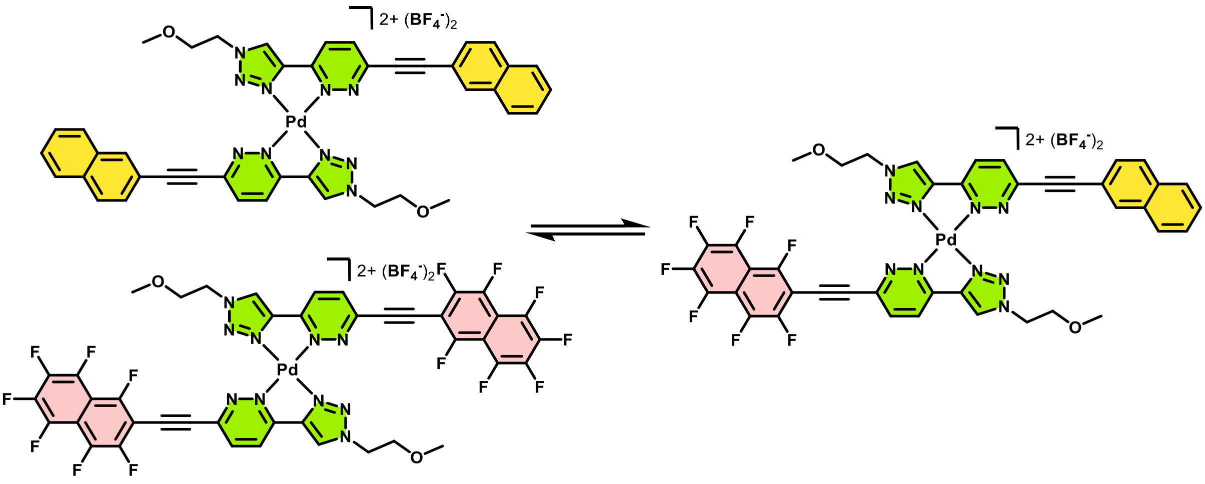

Stock solutions (7.5 mM in [D$_6$]DMSO) of **[Pd(AAER)$_2$]$^{2+}$** and **[Pd(AAEP)]$^{2+}$** were combined in 1:1 ratio to 1.88 mM and the mixture analysed by ^{1}H NMR spectroscopy and ESI-MS spectrometry. HR ESI-MS (DMSO/MeCN) *m/z* = 408.0963 [Pd(AAER)$_2$]$^{2+}$ (calcd for $C_{38}H_{24}N_8Pd$, 349.0592); 394.0750 [Pd(DAER)(ADER)]$^{2+}$ (calcd for $C_{42}H_{34}N_{10}O_2Pd$, 408.0958); 471.0636 [Pd(AAER)(AAEP)]$^{2+}$ (calcd for $C_{42}H_{27}F_7N_{10}O_2Pd$, 471.0621); 534.0307 [Pd(AAEP)$_2$]$^{2+}$ (calcd for $C_{42}H_{20}F_{14}N_{10}O_2Pd$, 534.0291).

A new complex appears in the ^{1}H NMR spectrum, and is presumably **[Pd(AAER)(AAEP)]$^{2+}$**, as this is the major species seen by ESI-MS. The naphthalene resonances remain broad, but do demonstrate upfield shifting, as seen in other heteroleptic complexes with ER···EP interactions. The ratio of **[Pd(AAER)$_2$]$^{2+}$**, **[Pd(AAEP)]$^{2+}$** and **[Pd(AAER)(AAEP)]$^{2+}$** cannot be measured due to signal overlap, but is estimated at 1:1:4 (cf. statistical ratio of 1:1:2).

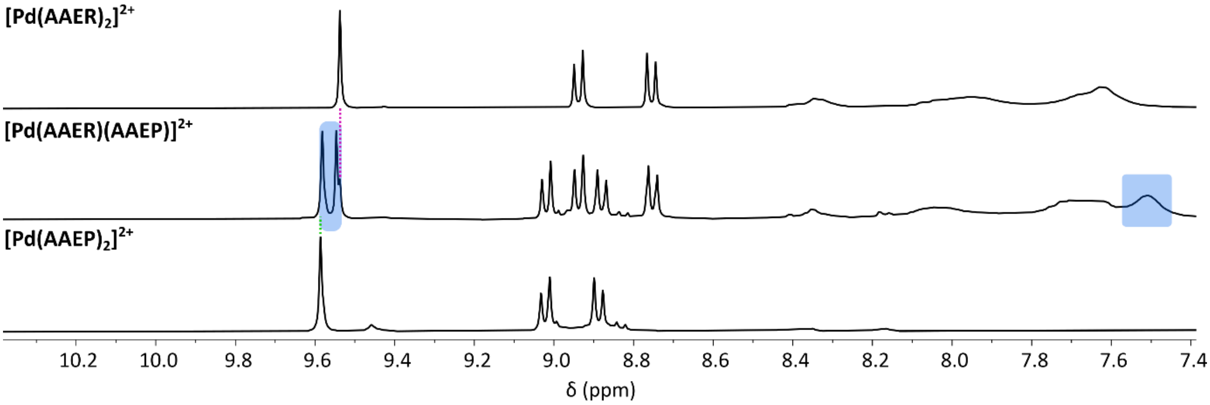

Figure S103. Partial stacked ^{1}H NMR ([D$_6$]DMSO, 400 MHz, 298 K) spectra of **[Pd(AAER)$_2$]$^{2+}$, [Pd(AAER)$_2$]$^{2+}$** and **[Pd(AAEP)$_2$]$^{2+}$** in a 1:1 ratio, and **Pd(DAER)$_2$]$^{2+}$.** Blue highlights possible new species.

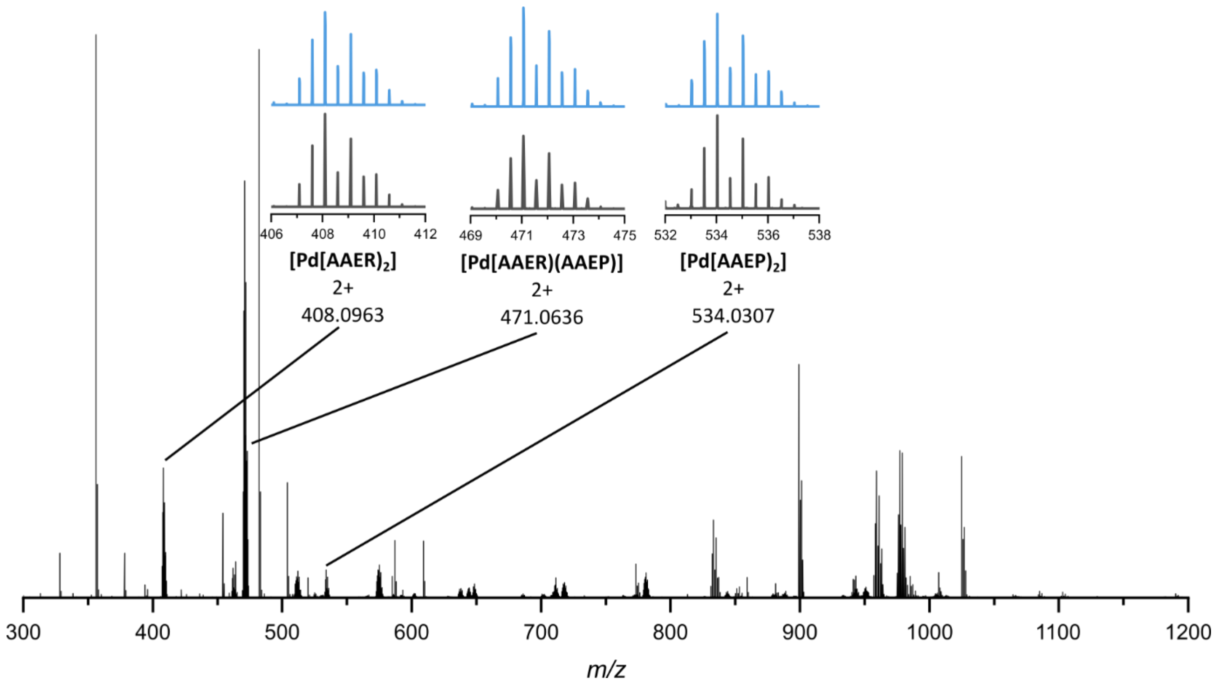

Figure S104. HR-ESI-MS(+) spectrum of **[Pd(AAER)(AAEP)](BF_4)$_2$.** Blue trace: calculated; black trace: found.

2.6. Scrambling Experiments

2.6.1. General

Pairs of the heteroleptic complexes prepared above (1.88 mM, [D$_6$]DMSO) were combined to a concentration of 0.94 mM of each species and the ^{1}H, ^{19}F NMR and mass-spectra recorded.

2.6.2. Combining [Pd(AAER)(DDEP)](BF$_4$)$_2$ and [Pd(AAEP)(DDER)](BF$_4$)$_2$

The major species [**Pd(AAER)(DDEP)]$^{2+}$** and **[Pd(AAEP)(DDER)]$^{2+}$** were observed.

HR ESI-MS (DMSO) *m/z* = 394.0828 [Pd(ADER)(DAER)]$^{2+}$ (calcd for $C_{45}H_{30}N_8Pd$, 394.0817); 412.0264 [Pd(ADER)(DAER)]$^{2+}$ (calcd for $C_{38}H_{17}F_7N_8Pd$, 412.0251); 457.0481 [Pd(ADEP)(DAER)]$^{2+}$, Pd(ADER)(DAEP)]$^{2+}$ (calcd for $C_{45}H_{23}F_7N_8Pd$, 457.0487); 502.0742 [Pd(ADER)(ADEP)]$^{2+}$ (calcd for $C_{52}H_{29}F_7N_8Pd$, 502.0722); 520.0177 [Pd(ADEP)(DAEP)]$^{2+}$ (calcd for $C_{45}H_{16}F_{14}N_8Pd$, 520.0157); 823.0420 [M-C_7H_7]$^+$ (calcd for $C_{38}H_{16}F_7N_8Pd$, 823.0429).

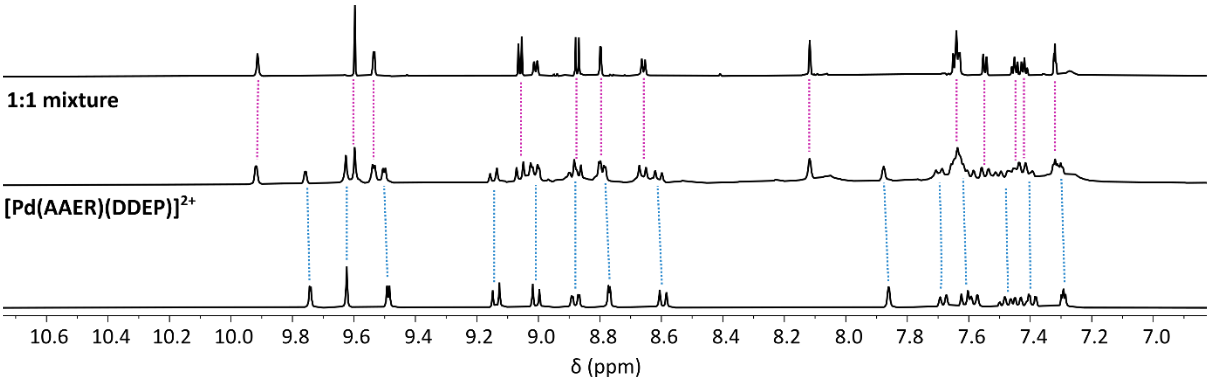

Figure S105. Partial stacked ^{1}H NMR ([D$_6$]DMSO, 400 MHz, 298K) spectra of **[Pd(AAEP)(DDER)]$^{2+}$**, a 1:1 mixture**,** and **Pd(AAER)(DDEP)]$^{2+}$**.

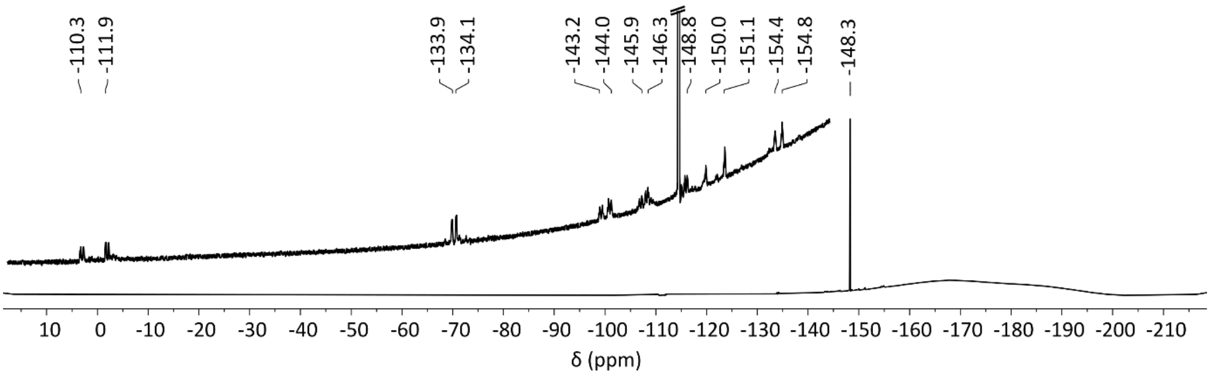

Figure S106. ^{19}F NMR ([D$_6$]DMSO, 400 MHz, 298K) of a 1:1 mixture of **[Pd(AAEP)(DDER)]$^{2+}$** and **Pd(AAER)(DDEP)]$^{2+}$.**

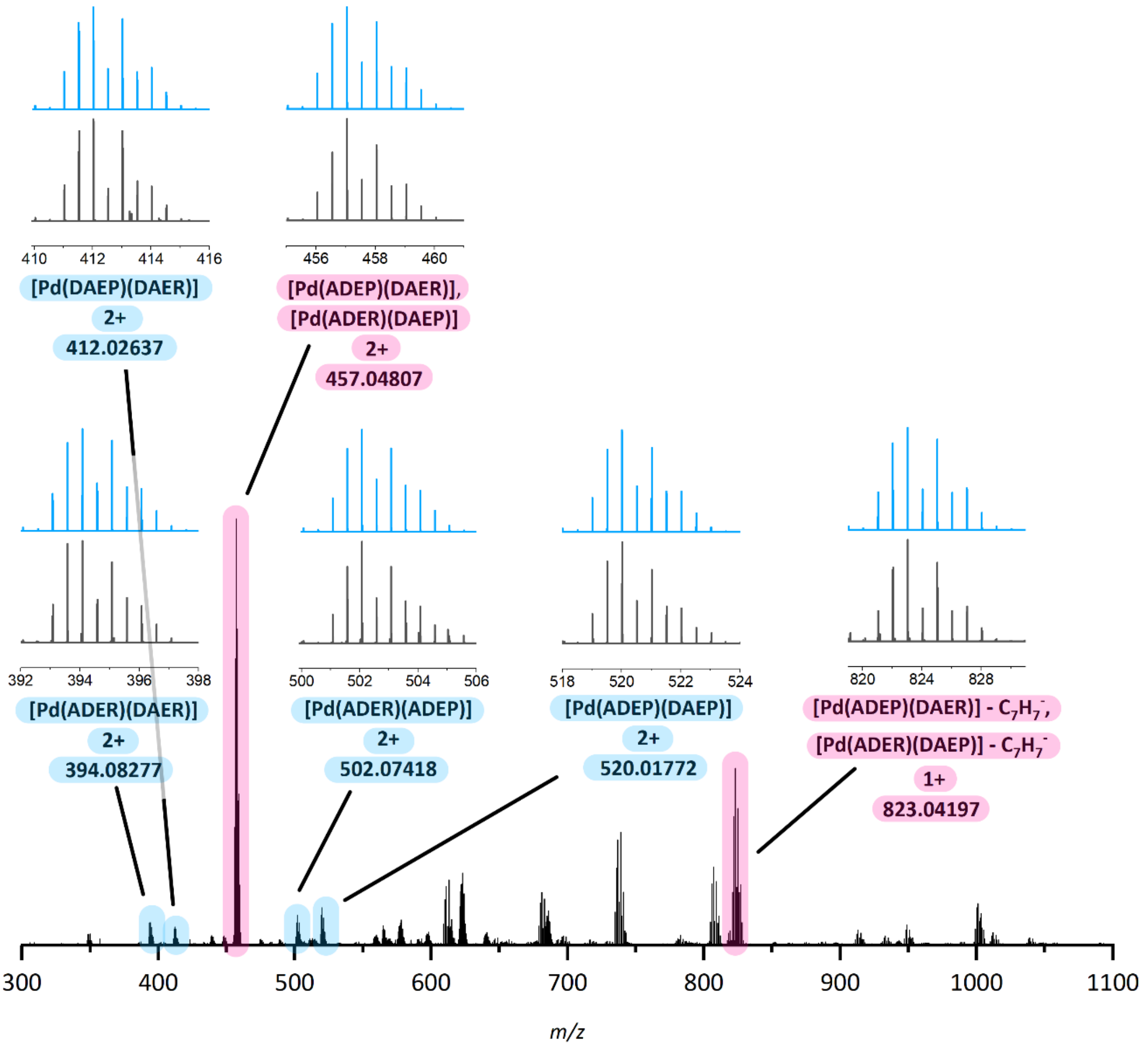

Figure S107. HR Nanospray-MS(+) spectrum of a 1:1 mixture of **[Pd(AAEP)(DDER)]$^{2+}$** and **Pd(AAER)(DDEP)]$^{2+}$.** Blue trace: calculated; black trace: found.

2.6.3. Combining [Pd(AAER)(DDEP)]$(BF_4)_2$ and [Pd(ADEP)(DAER)]$(BF_4)_2$

The major species **[Pd(AAER)(DDEP)]$^{2+}$** and **[Pd(ADEP)(DAER)]$^{2+}$** were observed. New minor species (<10%; highlighted in pink) are attributed to scrambled species **[Pd(AAER)(ADEP)]$^{2+}$** which is present in the ESI-MS spectrum.

HR ESI-MS (DMSO) *m/z* = 378.5771 [Pd(AAER)(DAER)]$^{2+}$ (calcd for $C_{40}H_{29}N_9OPd$, 378.5767); 441.0475 [Pd(AAER)(DDEP)]$^{2+}$ (calcd for $C_{41}H_{23}F_7N_8OPd$, 441.0461); 457.0481 [Pd(ADEP)(DAER)]$^{2+}$ (calcd for $C_{45}H_{23}F_7N_8Pd$, 457.0487); 486.5689 [Pd(AAER)(ADEP)]$^{2+}$ (calcd for $C_{47}H_{28}F_7N_9OPd$, 486.56725).

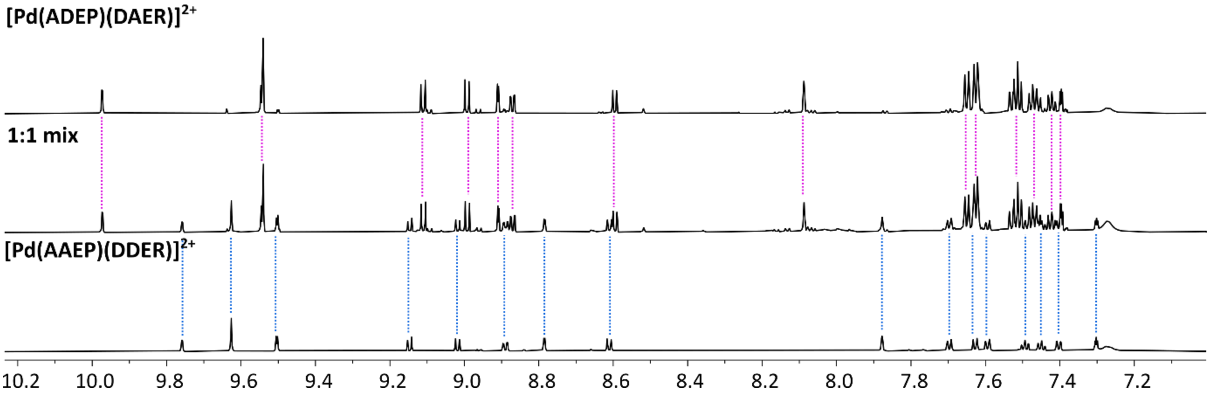

Figure S108. Partial stacked ^{1}H NMR ([D$_6$]DMSO, 400 MHz, 298K) spectra of **[Pd(AAER)(DDEP)]$^{2+}$**, a 1:1 mixture, and **Pd(ADEP)(DAER)]$^{2+}$**.

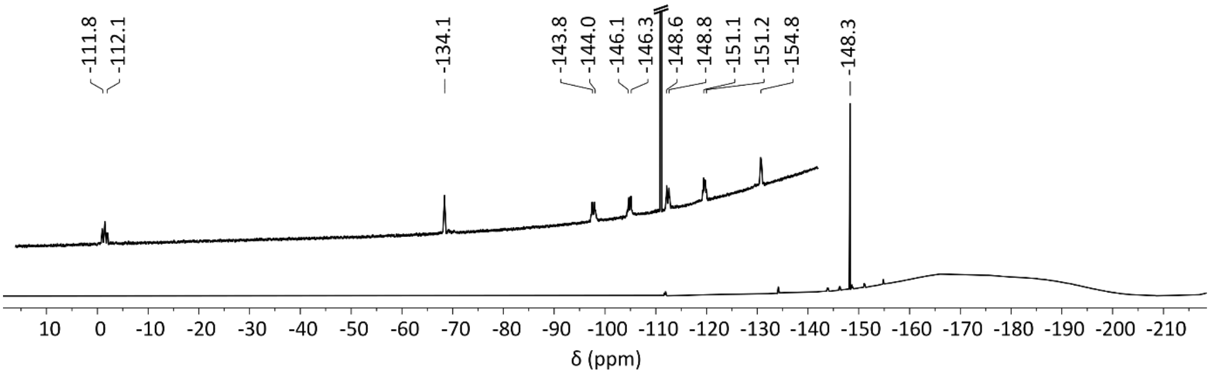

Figure S109. ^{19}F NMR ([D$_6$]DMSO, 400 MHz, 298K) of a 1:1 mixture of **[Pd(AAER)(DDEP)]$^{2+}$** and **Pd(ADEP)(DAER)]$^{2+}$**.

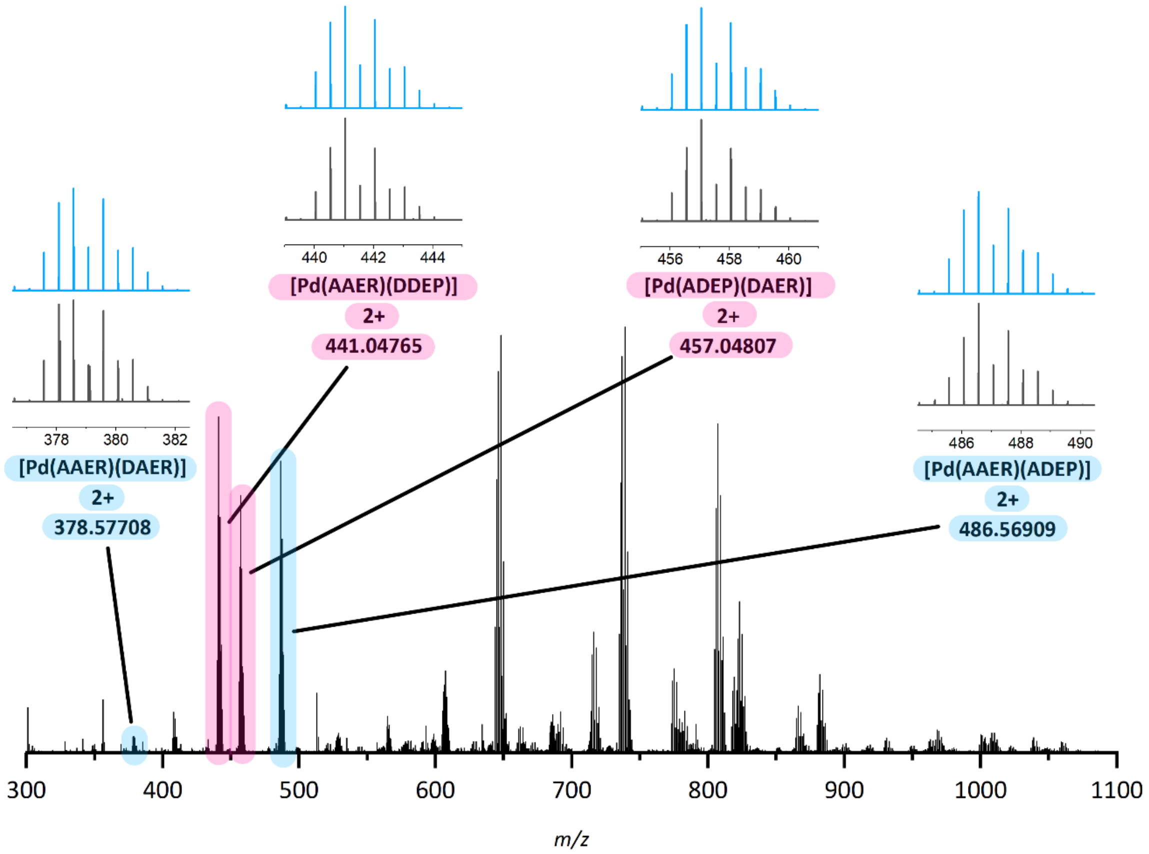

Figure S110. HR Nanospray-MS(+) spectrum of a 1:1 mixture of **[Pd(AAER)(DDEP)]$^{2+}$** and **Pd(ADEP)(DAER)]$^{2+}$**. Blue trace: calculated; black trace: found.

2.6.4. Combining [Pd(AAEP)(DDER)](BF_4)$_2$ and [Pd(ADEP)(DAER)](BF_4)$_2$

The major species **[Pd(AAEP)(DDER)]$^{2+}$** and **[Pd(ADEP)(DAER)]$^{2+}$** were observed.

HR ESI-MS (DMSO) *m/z* = 441.0475 [Pd(AAEP)(DDER)]$^{2+}$ (calcd for $C_{41}H_{23}F_7N_8OPd$, 441.0461); 457.0481 [Pd(ADEP)(DAER)]$^{2+}$ (calcd for $C_{45}H_{23}F_7N_8Pd$, 457.0487); 549.5361 [Pd(AAEP)(DAEP)]$^{2+}$ (calcd for $C_{47}H_{21}F_{14}N_9OPd$, 549.5335).

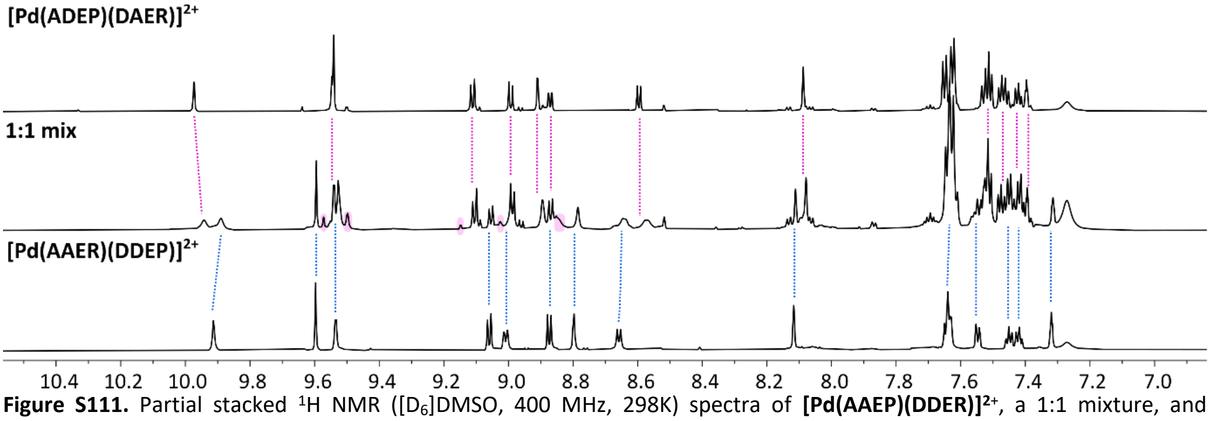

Figure S111. Partial stacked ^1H NMR ([D$_6$]DMSO, 400 MHz, 298K) spectra of **[Pd(AAEP)(DDER)]$^{2+}$**, a 1:1 mixture, and **Pd(ADEP)(DAER)]$^{2+}$.** Pink highlights a new, minor species.

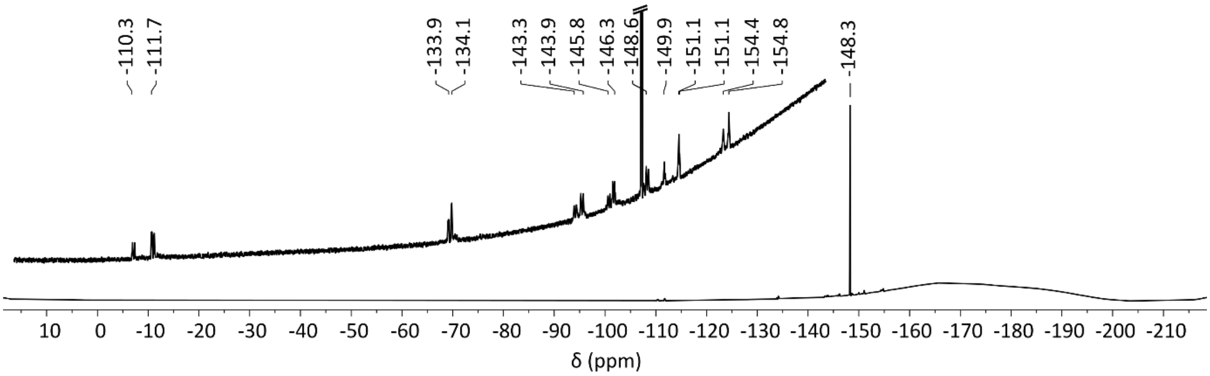

Figure S112. ^{19}F NMR ([D$_6$]DMSO, 400 MHz, 298K) of a 1:1 mixture of **[Pd(AAEP)(DDER)]$^{2+}$** and **Pd(ADEP)(DAER)]$^{2+}$.**

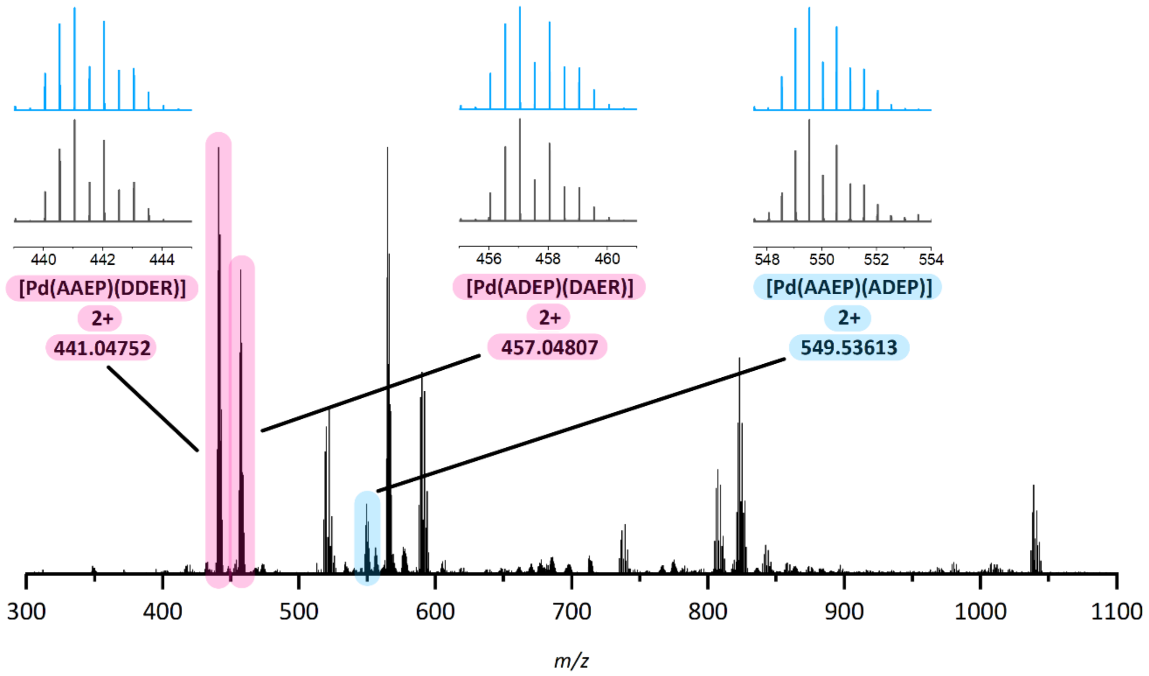

Figure S113. HR Nanospray-MS(+) spectrum of a 1:1 mixture of **[Pd(AAEP)(DDER)]$^{2+}$** and **Pd(ADEP)(DAER)]$^{2+}$.** Blue trace: calculated; black trace: found.

2.6.5. Combining all homoleptic species

Stock solutions of the seven homoleptic species were combined in an equimolar ratio (0.47 mM) in [D$_6$]DMSO and the ^{1}H NMR spectrum recorded. The ^{1}H spectrum showed the three heteroleptic species and [Pd(**ADER**)$_2$]$^{2+}$. No meaningful amounts of scrambled species are observed. Minor changes in chemical shift upon combination are attributed to intermolecular interactions in solution. Scrambled species are seen by ESI-MS as minor components.

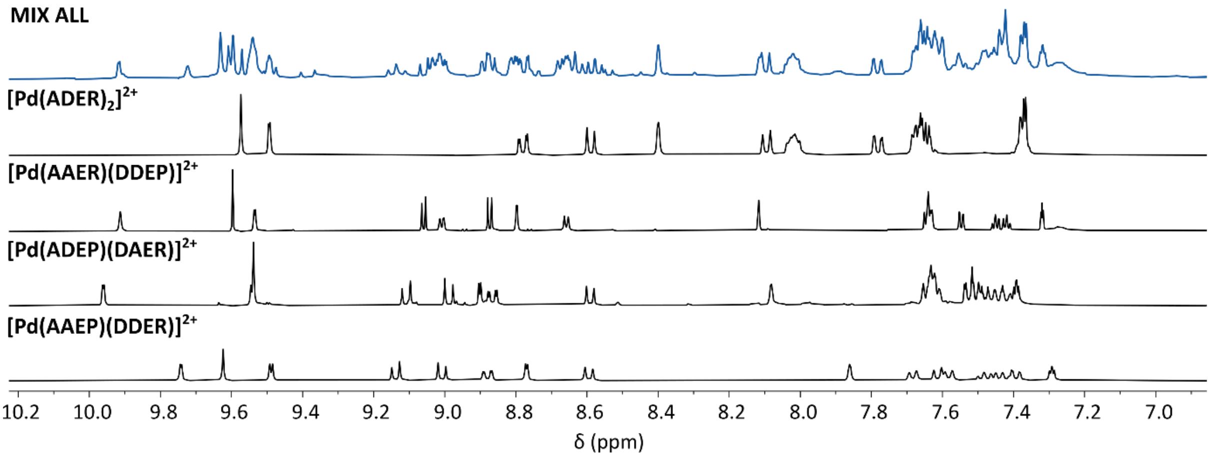

Figure S114. Partial stacked ^{1}H NMR ([D$_6$]DMSO, 400 MHz, 298K) spectra of the combination of all seven homoleptic species (top, blue), **[Pd(ADER)$_2$]$^{2+}$**, and the three heteroleptic species.

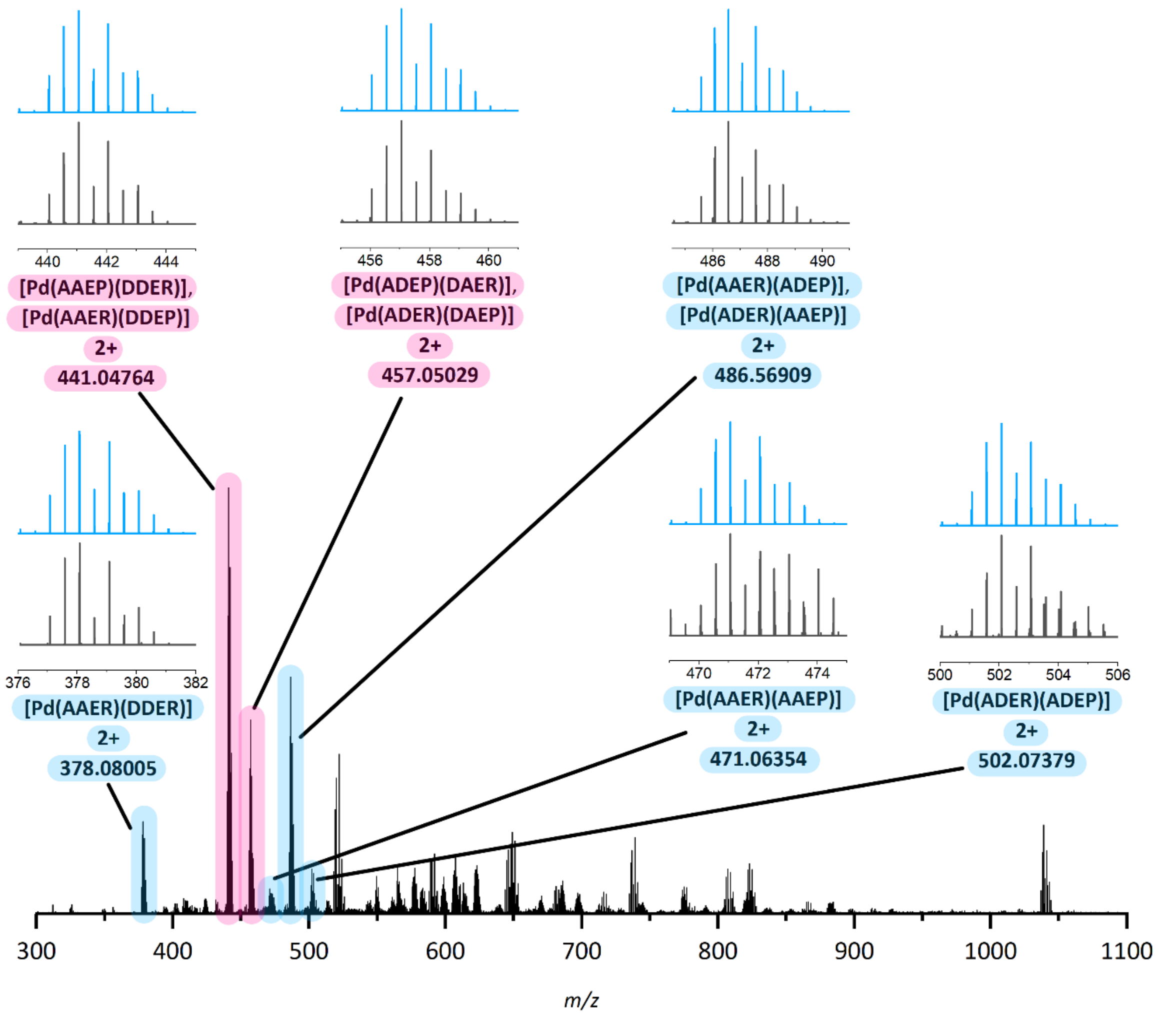

Figure S115. HR Nanospray-MS(+) spectrum of a mixture of the seven homoleptic species. Blue trace: calculated; black trace: found.

Additional Pd(II) was added in 0.25 eq. aliquots (based on total Pd(II) in sample). The heteroleptic species remained largely unchanged, while the homoleptic species **$[Pd(ADER)_2]^{2+}$** shifted to the formation of **$[Pd(ADER)(DMSO)_2]^{2+}$**. Growing signals attributed to **$[Pd(ADER)(DMSO)_2]^{2+}$** are highlighted in pink.

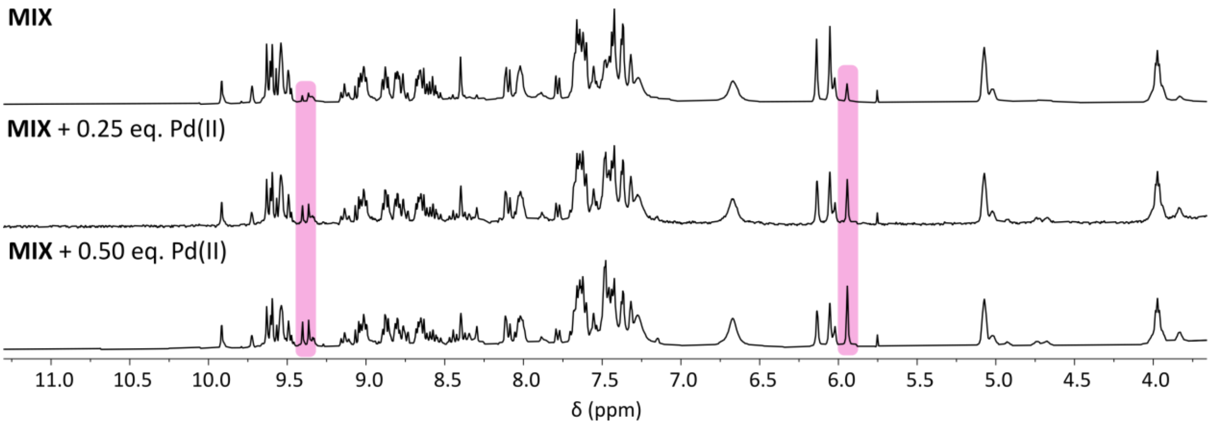

Figure S116. Partial stacked ^{1}H NMR ([D$_6$]DMSO, 400 MHz, 298K) spectra of the mixture of the seven homoleptic species, with the sequential addition of Pd(II). Pink highlights growing **$[Pd(ADER)(DMSO)_2]^{2+}$.**

3. Calculations

3.1. General

To investigate potential changes in energy between HT or HH arrangements, we constructed the initial idealised representation of the isomers without counter anions using *Spartan24*.[72]

These structures were then used as the starting geometries for the GFN2-xTB molecular dynamics (MD) calculations. All simulations were conducted using quantum mechanical extended tight binding method GFN2-xTB 6.4.0,[66] as implemented in the *xtb* code, with an initial "loose" optimization [loose: E_{conv} (energy convergence) = 5×10^{-5} E_{h}; G_{conv} (gradient convergence) = 4×10^{-3} $E_{\mathrm{h}}\cdot\alpha^{-1}$; accuracy (for integral cutoffs and SCF criteria) = 2.00] and the solvent DMSO was represented with an implicit solvation model (ALPB, as implemented in the xTB code).[73] Lebedev grid level used was "normal" with 230 grid points (as implemented in the xTB code).[74] The simulations were conducted in the NVT ensemble with the system temperature being maintained at 298 K. All bonds were constrained using the SHAKE algorithm.[75] Each simulation was run for 1000 ps, with trajectory output every 50 fs, with a propagation time step of 4 fs. Energies were obtained from averaging the energy from each structure over the course of the simulation.

To further understand the steric and electronic contributions to the stability of the xTB optimised complexes, geometries HH and HT were then optimised using density functional theory (DFT) calculations, which were performed using the ORCA program version 6.0.1.[76] Structures were fully optimized using a slightly modified r^{2}SCAN-3c composite method[67, 68] with C and H atoms treated by the def2-SVP basis set and all other atoms (N, O, Pd) treated by the def2-TZVP basis set.[77] The Def2-ECP effective core potential was used for Pd[78] and dispersion interactions treated using the D4 approach.[79] Structures were optimised with tight convergence criteria on both the geometry and self-consistent field (SCF) cycles using this modified basis. The conductor-like polarizable continuum model (CPCM) was used to treat the structures in a DMSO environment.

3.2. Calculated energies

Table S4. Summary of calculated energies using XTB and DFT.

	XTB			DFT		
**complex**	**major isomer**	***ΔG* (kJ mol^{-1})^{a}**	**ratio HT:HH**	**major isomer**	***ΔG* (kJ mol^{-1})^{a}**	**ratio HT:HH**
**$[Pd(ADER)_2]^{2+}$**	HT	−25.1	>99:1	-	-	-
**$[Pd(DAER)_2]^{2+}$**	HT	−10.2	2:98	-	-	-
**$[Pd(AAER)_2]^{2+}$**	HH	−10.1	2:98	HH	−16.7	<1:99
**$[Pd(AAEP)_2]^{2+}$**	HT	−6.1	92:8	-	-	-
**$[Pd(DDER)_2]^{2+}$**	HT	−3.0	77:23	-	-	-
**$[Pd(AA\text{-}H)_2]^{2+}$**	HT	−5.2	89:11	HT	−3.45	80:20
**$[Pd(DD\text{-}H)_2]^{2+}$**	HT	−2.4	72:28	-	-	-
**$[Pd(AA\text{-}TMS)_2]^{2+}$**	HT	−15.2	>99:1	-	-	-
**$[Pd(AD\text{-}TMS)(DA\text{-}TMS)]^{2+}$**	HH	−6.0	8:92	-	-	-
**$[Pd(AA\text{-}TMS)(DD\text{-}TMS)]^{2+}$**	HT	−9.5	2:98	-	-	-

^{a}*ΔG* reported for the formation of the major isomer.

3.3. Molecular dynamics simulations

Below are given key data extracted from the molecular dynamics simulations.

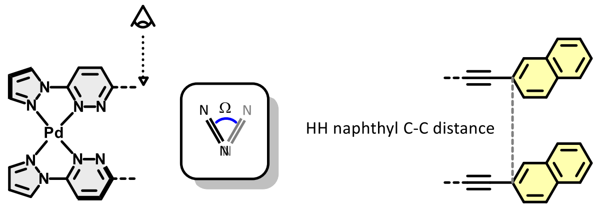

Figure S117. Key parameters investigated during simulations: dihedral angle (Ω) and napthyl C-C distance.

3.3.1. $[Pd(ADER)_2]^{2+}$ and $[Pd(DAER)_2]^{2+}$

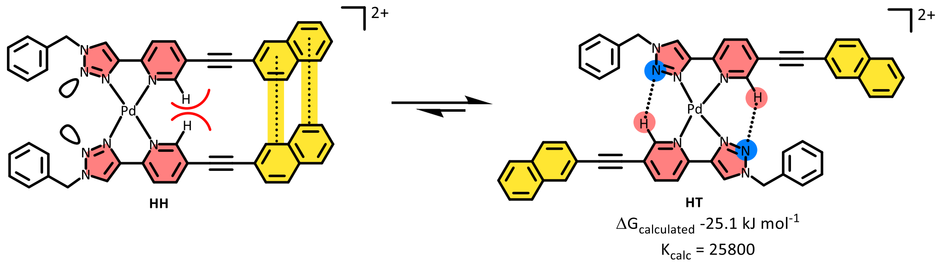

Figure S118. Equilibrium between HH and HT $[Pd(\mathbf{ADER})_2]^{2+}$, with ΔG and K shown under the computationally energetically preferred orientation.

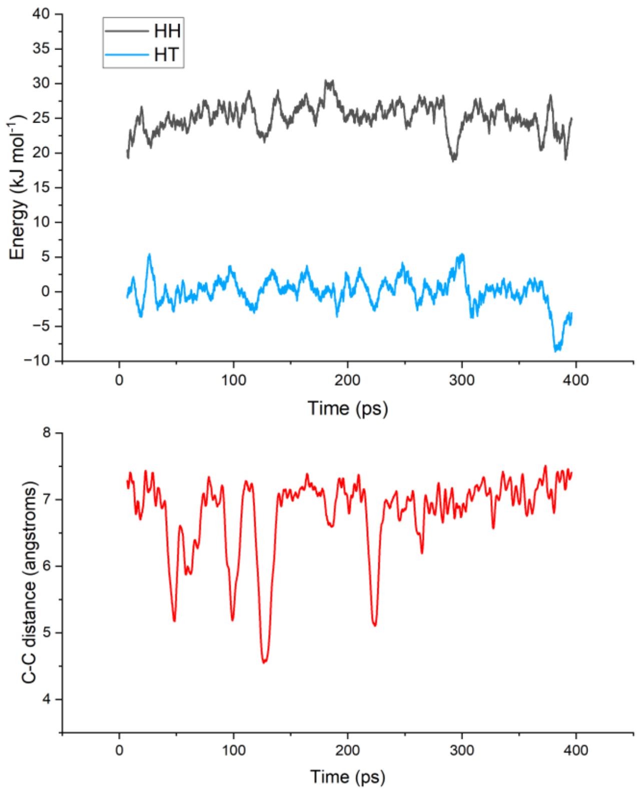

Figure S119. *Top:* relative energies between HH and HT isomers of $[Pd(\mathbf{ADER})_2]^{2+}$, *bottom:* C-C distance between naphthyl units for the HH isomer.

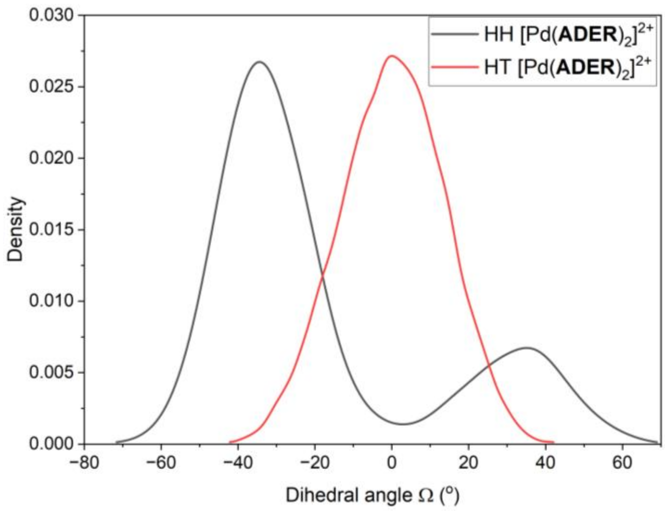

Figure S120. *Top:* Dihedral angles between HH and HT isomers of $[Pd(\mathbf{ADER})_2]^{2+}$.

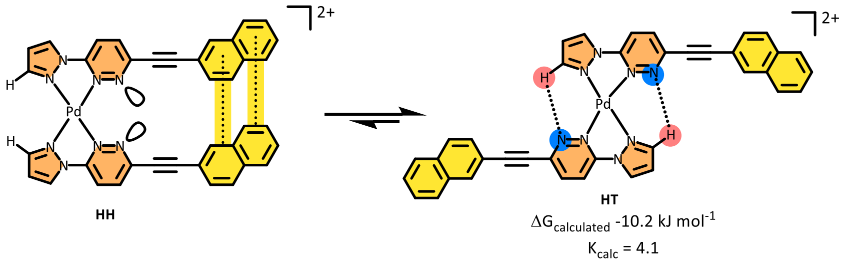

Figure S121 Equilibrium between HH and HT $[Pd(\mathbf{DAER})_2]^{2+}$, with ΔG and K shown under the computationally energetically preferred orientation.

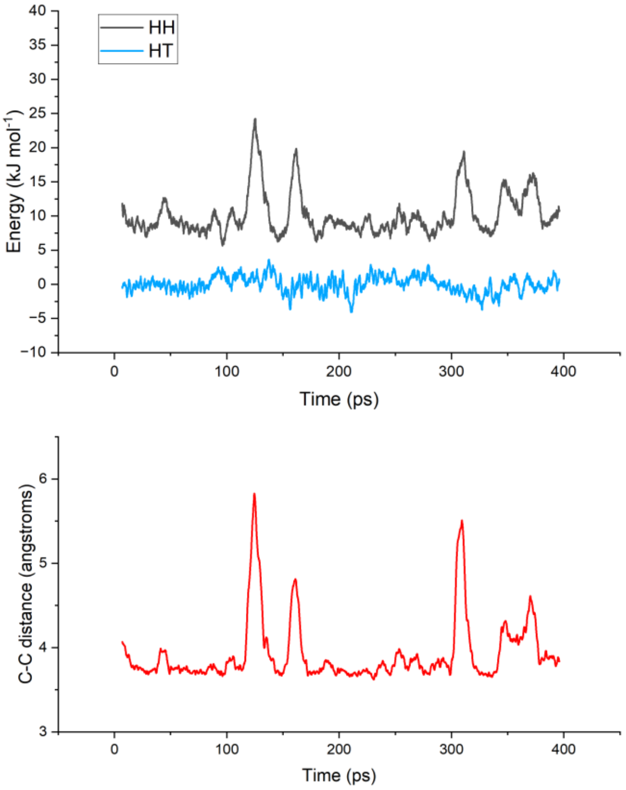

Figure S122. *Top:* relative energies between HH and HT isomers of [Pd(**DAER**)$_2$]$^{2+}$, *bottom:* C-C distance between naphthyl units for the HH isomer.

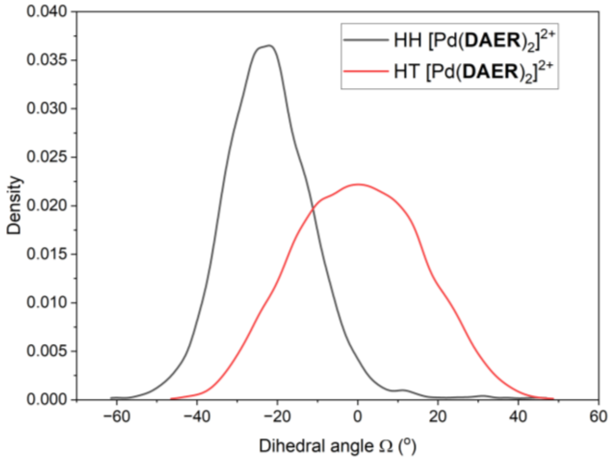

Figure S123. *Top:* Dihedral angles between HH and HT isomers of [Pd(**DAER**)$_2$]$^{2+}$.

3.3.2. [Pd(AAER)$_2$]$^{2+}$

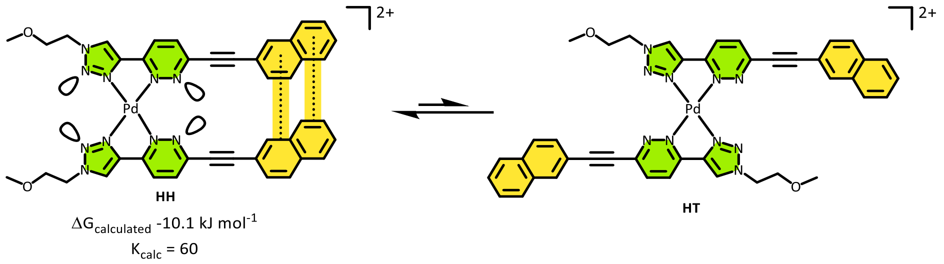

Figure S124. Equilibrium between HH and HT **[Pd(AAER)$_2$]$^{2+}$,** with ΔG and K shown under the computationally energetically preferred orientation.

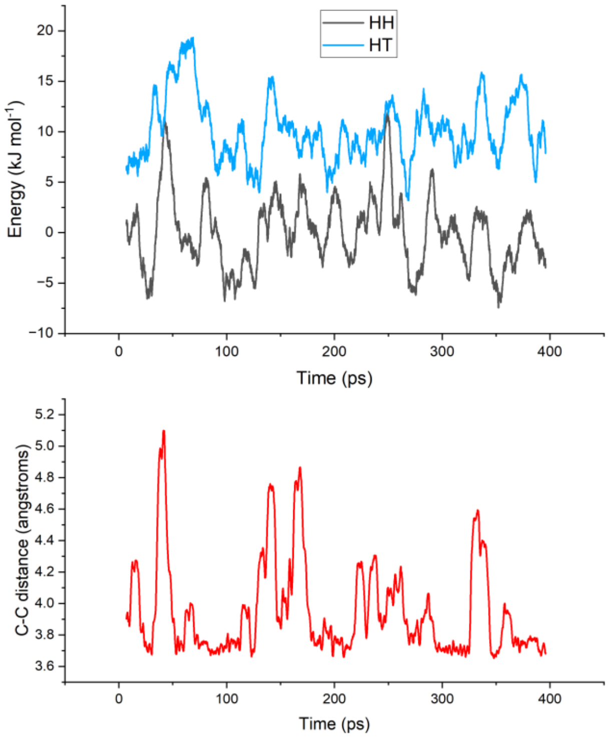

Figure S125. *Top:* relative energies between HH and HT isomers of **[Pd(AAER)$_2$]$^{2+}$**, *bottom:* C-C distance between naphthyl units for the HH isomer.

3.3.3. [Pd(AAEP)$_2$]$^{2+}$

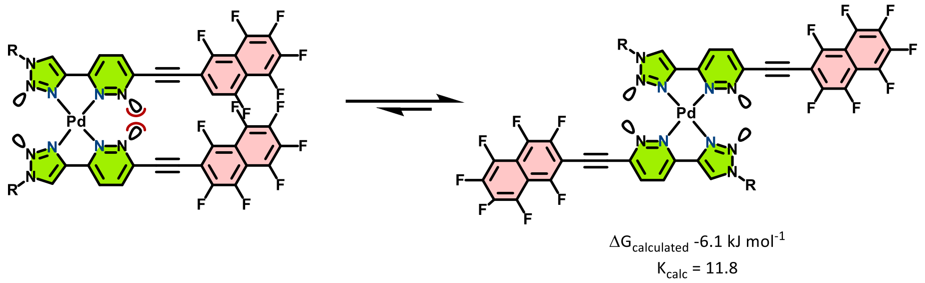

Figure S126. Equilibrium between HH and HT **[Pd(AAEP)$_2$]$^{2+}$,** with ΔG and K shown under the computationally energetically preferred orientation.

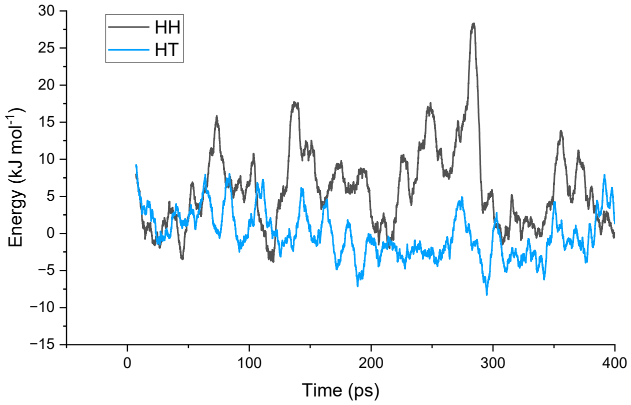

Figure S127. Relative energies between HH and HT **[Pd(AAEP)$_2$]$^{2+}$.**

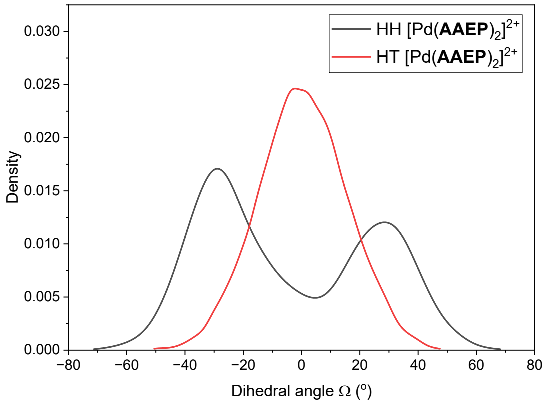

Figure S128. *Top:* Dihedral angles between HH and HT **[Pd(AAEP)$_2$]$^{2+}$.**

3.3.4. [Pd(AA-H)$_2$]$^{2+}$ and [Pd(DD-H)$_2$]$^{2+}$

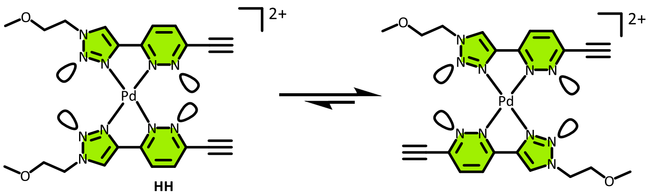

ΔG$_{calculated}$ -5.2 kJ mol^{-1}

K$_{calc}$ = 8.2

Figure S129. Equilibrium between HH and HT **[Pd(AA-H)$_2$]$^{2+}$,** with ΔG and K shown under the computationally energetically preferred orientation.

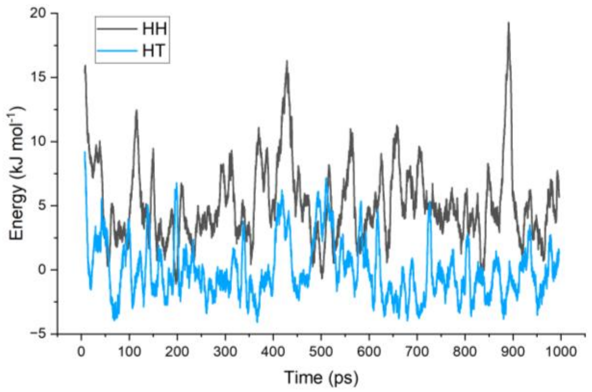

Figure S130. Relative energies between HH and HT isomers of **[Pd(AA-H)$_2$]$^{2+}$**.

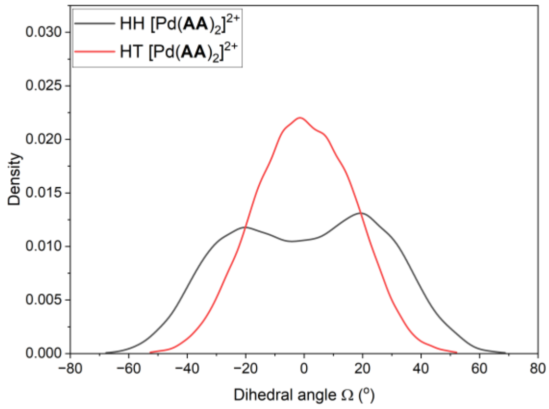

Figure S131. *Top:* Dihedral angles between HH and HT isomers of **[Pd(AA-H)$_2$]$^{2+}$.**

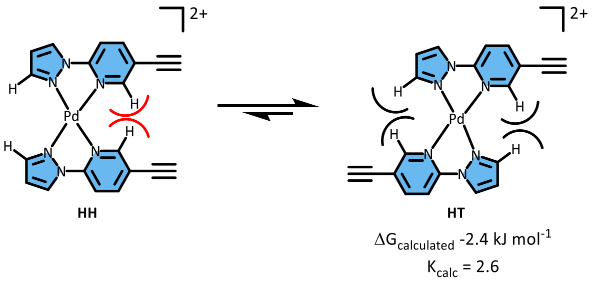

Figure S132. Equilibrium between HH and HT **[Pd(DD-H)$_2$]$^{2+}$,** with ΔG and K shown under the computationally energetically preferred orientation.

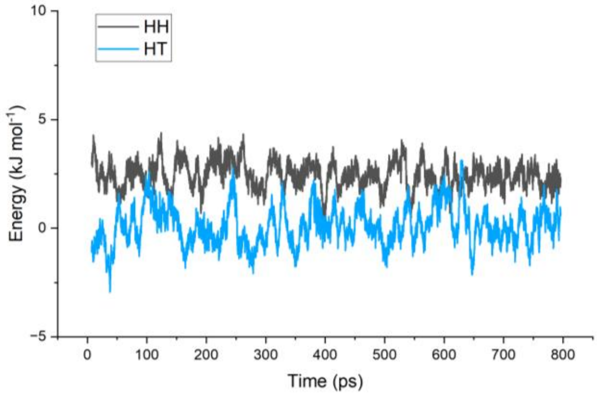

Figure S133. Relative energies between HH and HT isomers of **[Pd(DD-H)$_2$]$^{2+}$.**

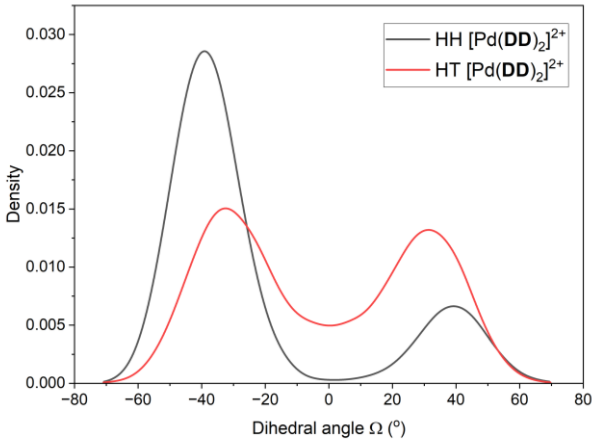

Figure S134. *Top:* Dihedral angles between HH and HT isomers of **[Pd(DD-H)$_2$]$^{2+}$.**

3.3.5. [Pd(AD-TMS)(DA-TMS)]$^{2+}$

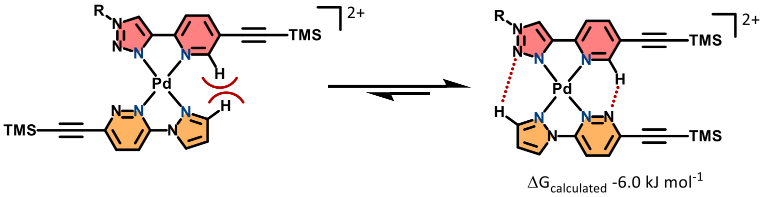

Figure S135. Equilibrium between HH and HT **[Pd(AD-TMS)(DA-TMS)]$^{2+}$,** with ΔG and K shown under the computationally energetically preferred orientation.

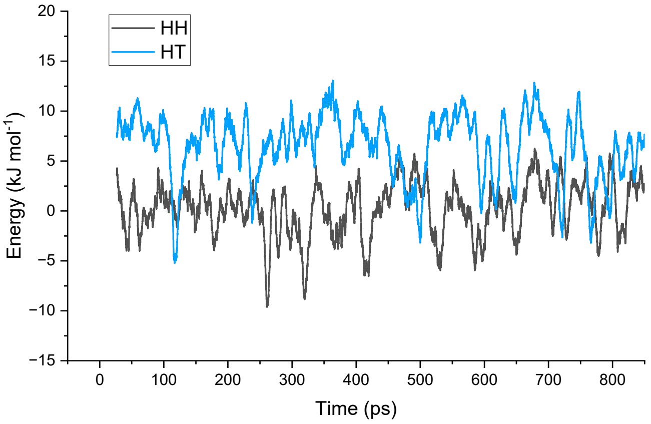

Figure S136. Relative energies between HH and HT **[Pd(AD-TMS)(DA-TMS)]$^{2+}$.**

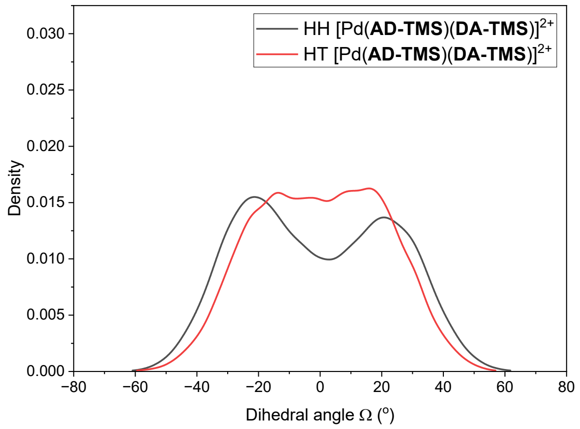

Figure S137. *Top:* Dihedral angles between HH and HT **[Pd(AD-TMS)(DA-TMS)]$^{2+}$.**

4. Crystallography

4.1. Experimental data

Single crystal data were collected using mirror-monochromated Mo Kα radiation on an Agilent XCalibur diffractometer or mirror-monochromated Cu Kα radiation on an Agilent SuperNova diffractometer at 150 K. Raw frame data (including data reduction, interframe scaling, unit cell refinement and absorption corrections) were processed using CrysAlis Pro.[80] All structures were solved by direct methods using SHELXT2014[81, 82] and refined against all F2 data using SHELXL2014[81] and the OLEX2[83] interface. The data are summarised in Table S5. Some complexes were crystallised from mixtures containing anthracene.

Table S5. Summary of crystallographic data.

	**$[Pd(AAER)_2](BF_4^-)_2$**	**$[Pd(ADEP)_2](BF_4^-)_2$**	**$[Pd(DA\text{-}TMS)_2](BF_4^-)_2{\cdot}C_{14}H_{10}$**
**CCDC No.**	**2475052**	**2475053**	**2475054**
Formula	$C_{46}H_{46}B_2F_8N_{10}O_4PdS_2$	$C_{52}H_{22}B_2F_{22}N_8Pd$	$C_{38}H_{38}B_2F_8N_8PdSi_2$
M	1147.07	1304.79	942.96
T (K)	150	150	150
Crystal system	monoclinic	orthorhombic	triclinic
Space group	P21/*c*	P212121	*P*-1
a (Å)	12.4056(2)	8.4701(2)	6.8443(3)
b (Å)	20.8325(2)	14.9586(3)	15.3947(7)
c (Å)	10.1165(1)	39.163(1)	20.0455(10)
α (°)	90	90	99.335(4)
β (°)	106.822(1)	90	95.050(4)
γ (°)	90	90	96.880(4)
V (Å^3)	2502.63(5)	4962.0(2)	2068.82(17)
Z [*Z′*]	2 [1]	4 [1]	2 [1]
Crystal description	orange block	orange block	orange block
Crystal size (mm^3)	0.4 × 0.1 × 0.1	0.2 × 0.1 × 0.02	0.2 × 0.05 × 0.04
μ (mm^{-1})	4.501	4.217	4.837
$2\vartheta_{full}$, $2\vartheta_{max}$ (°)	134.00, 136.49	134.00, 136.46	95.80, 95.80
$N_{measured\ refl}$	23147	15828	16295
$N_{independent\ refl}$ [R_{int}]	4562 [0.0379]	8319 [0.0482]	3818 [0.0727]
$N_{observed\ refl}$ [$I > 2\sigma(I)$]	3962	7860	3337
$N_{parameters}$	334	812	538
$N_{restraints}$	0	80	276
R [$I > 2\sigma(I)$]	0.0421	0.0638	0.0666
wR [all data]	0.1162	0.1524	0.1706
GOF	1.052	1.147	1.068
$\Delta\rho_{max}$, $\Delta\rho_{min}$ (e Å^{-3})	1.100, –0.551	1.016, –1.178	1.055, –1.111
PLATON SQUEEZE	-	-	-
(per unit cell)	-	-	-

Table S5. (*cont.*) Summary of crystallographic data.

	**$[Pd(AD\text{-}TMS)_2](BF_4^-)_2{\cdot}C_{14}H_{10}$**
**CCDC No.**	**2475055**
Formula	$C_{52}H_{50}B_2F_8N_8PdSi_2$
M	1123.20
T (K)	150
Crystal system	triclinic
Space group	*P*-1
a (Å)	13.2675(5)
b (Å)	17.5108(6)
c (Å)	20.1995(9)
α (°)	108.535(4)
β (°)	102.462(3)
γ (°)	104.400(3)
V (Å^3)	4081.5(3)
Z [*Z′*]	3 [1.5]
Crystal description	orange block
Crystal size (mm^3)	0.2 × 0.1 × 0.02
μ (mm^{-1})	0.455
$2\vartheta_{full}$, $2\vartheta_{max}$ (°)	50.00, 58.47
$N_{measured\ refl}$	39807
$N_{independent\ refl}$ [R_{int}]	17831 [0.0593]
$N_{observed\ refl}$ [$I > 2\sigma(I)$]	10995
$N_{parameters}$	1060
$N_{restraints}$	148
R [$I > 2\sigma(I)$]	0.0800
wR [all data]	0.2546
GOF	1.056
$\Delta\rho_{max}$, $\Delta\rho_{min}$ (e Å^{-3})	2.563, –1.028
PLATON SQUEEZE	-
(per unit cell)	-

4.2. ORTEP diagrams

[Pd(AAER)₂](BF₄⁻)₂: the main molecule, solvent and counterions were well behaved and required no restraints.

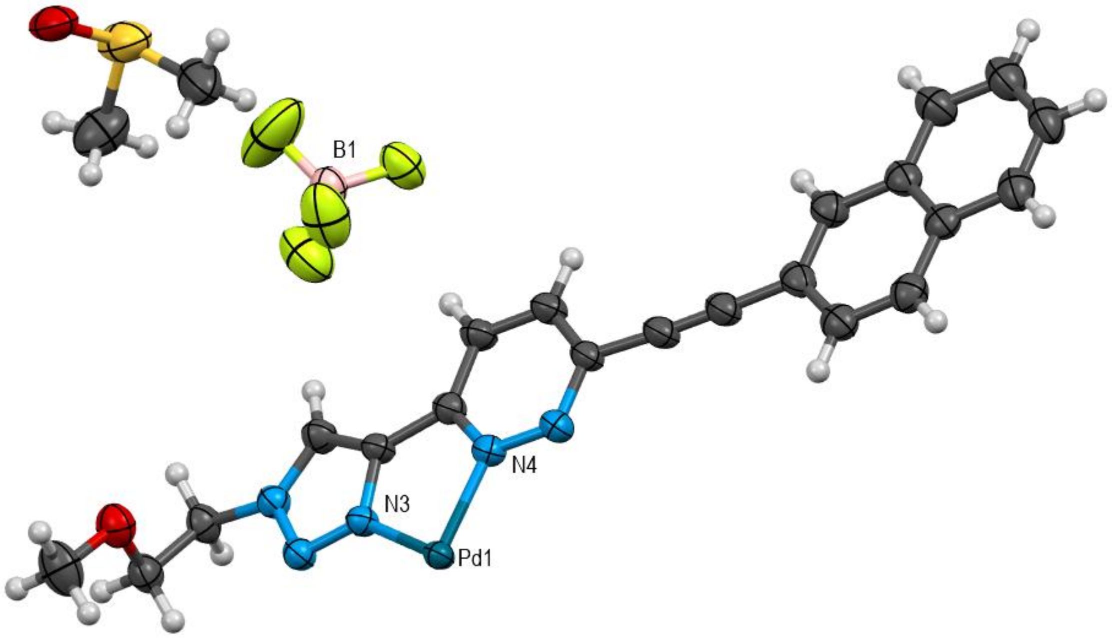

Figure S138. ORTEP diagram of the ASU of **$[Pd(AAER)_2](BF_4)_2$**·DMSO. Ellipsoids are shown to the 50% probability level.

$[Pd(ADEP)_2](BF_4^-)_2$: the main molecule was well behaved and required no restraints. One BF_4^- counterion was disordered over two sites and was modelled using ADP and geometric restraints.

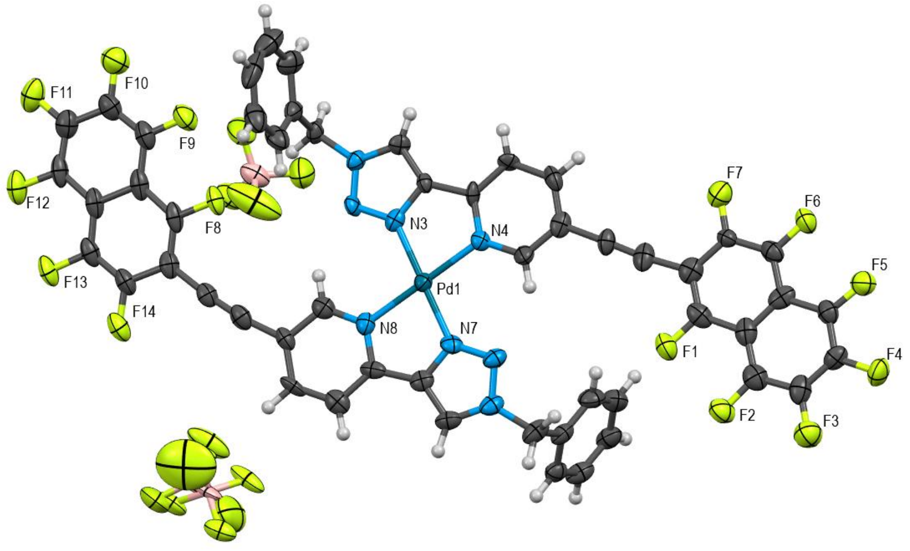

Figure S139. ORTEP diagram of the ASU of **$[Pd(ADEP)_2](BF_4)_2$**. Ellipsoids are shown to the 50% probability level.

[Pd(DA-TMS)$_2$](BF$_4^-$)$_2$·C$_{14}$H$_{10}$: the main molecule was modelled using ADP restraints. Anthracene and counterions were well behaved.

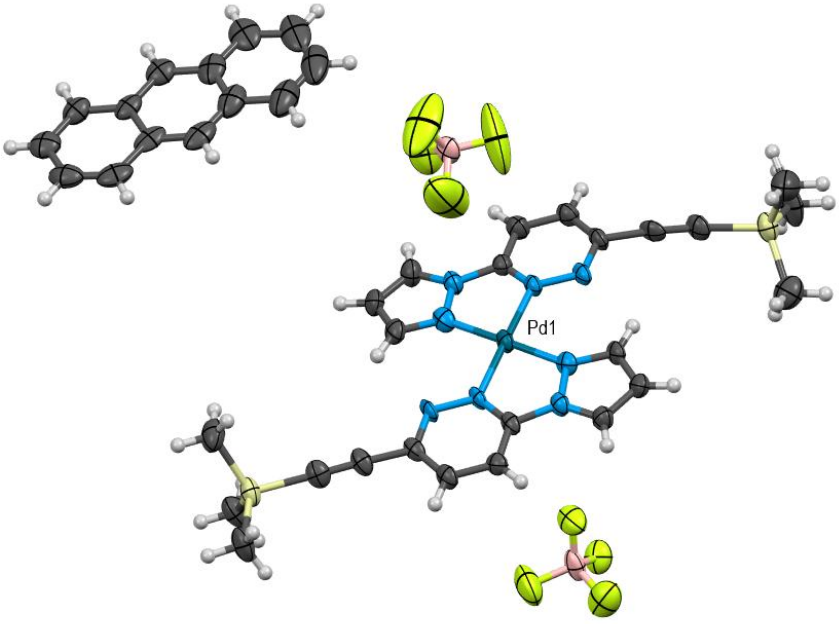

Figure S140. ORTEP diagram of the ASU of **[Pd(ADEP)$_2$](BF$_4$)$_2$**·anthracene. Ellipsoids are shown to the 50% probability level.

[Pd(AD-TMS)$_2$](BF$_4^-$)$_2$·C$_{14}$H$_{10}$: the main molecule was well behaved and required no restraints. One molecule of anthracene is likely disordered over an inversion centre, and was modelled using geometric and ADP restraints.

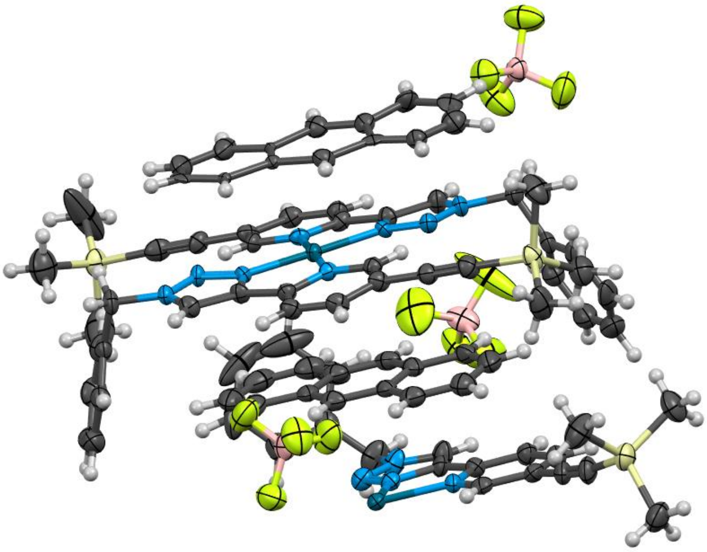

Figure S141. ORTEP diagram of the ASU of **[Pd(DAER)$_2$](BF$_4$)$_2$**·anthracene. Ellipsoids are shown to the 50% probability level.

5. References

[66] C. Bannwarth, S. Ehlert, S. Grimme, *J. Chem. Theory Comput.* **2019**, *15*, 1652-1671.

[67] S. Grimme, A. Hansen, S. Ehlert, J.-M. Mewes, *J. Chem. Phys.* **2021**, *154*.

[68] M. Bursch, J. M. Mewes, A. Hansen, S. Grimme, *Angew. Chem.* **2022**, *134*, e202205735.

[69] B. Happ, G. M. Pavlov, E. Altuntas, C. Friebe, M. D. Hager, A. Winter, H. Görls, W. Günther, U. S. Schubert, *Chem. Asian J.* **2011**, *6*, 873-880.

[70] C. Bianchini, G. Giambastiani, I. G. Rios, A. Meli, W. Oberhauser, L. Sorace, A. Toti, *Organometallics* **2007**, *26*, 5066-5078.

[71] D. Yang, J. L. Greenfield, T. K. Ronson, L. K. S. von Krbek, L. Yu, J. R. Nitschke, *J. Am. Chem. Soc.* **2020**, *142*, 19856-19861.

[72] *Spartan24*, Wavefunction Inc. **2025**.

[73] G. Sigalov, A. Fenley, A. Onufriev, *J. Chem. Phys.* **2006**, *124*.

[74] V. I. Lebedev, *Sib. Math. J.* **1977**, *18*, 99-107.

[75] J.-P. Ryckaert, G. Ciccotti, H. J. C. Berendsen, *J. Comput. Phys.* **1977**, *23*, 327-341.

[76] F. Neese, *WIREs Comput. Mol. Sci.*, **2022**.

[77] F. Weigend, R. Ahlrichs, *Phys. Chem. Chem. Phys.* **2005**, *7*, 3297-3305.

[78] D. Andrae, U. Häußermann, M. Dolg, H. Stoll, H. Preuß, *Theor. Chim. Acta* **1990**, *77*, 123-141.

[79] E. Caldeweyher, J.-M. Mewes, S. Ehlert, S. Grimme, *Phys. Chem. Chem. Phys.* **2020**, *22*, 8499-8512.

[80] *CrysAlisPro*, Agilent Technologies (Yarnton, Oxfordshire, UK), **2019**.

[81] G. M. Sheldrick, *Acta Crystallogr. Sect. A: Found. Crystallogr.* **2015**, *71*, 3-8.

[82] G. M. Sheldrick, *Acta Crystallogr. Sect. A: Found. Crystallogr.* **2008**, *64*, 112-122.

[83] O. V. Dolomanov, L. J. Bourhis, R. J. Gildea, J. A. K. Howard, H. Puschmann, *J. Appl. Crystallogr.* **2009**, *42*, 339-341.
